# Large-scale Pan-cancer Cell Line Screening Identifies Actionable and Effective Drug Combinations

**DOI:** 10.1158/2159-8290.CD-23-0388

**Published:** 2024-03-08

**Authors:** Azadeh C. Bashi, Elizabeth A. Coker, Krishna C. Bulusu, Patricia Jaaks, Claire Crafter, Howard Lightfoot, Marta Milo, Katrina McCarten, David F. Jenkins, Dieudonne van der Meer, James T. Lynch, Syd Barthorpe, Courtney L. Andersen, Simon T. Barry, Alexandra Beck, Justin Cidado, Jacob A. Gordon, Caitlin Hall, James Hall, Iman Mali, Tatiana Mironenko, Kevin Mongeon, James Morris, Laura Richardson, Paul D. Smith, Omid Tavana, Charlotte Tolley, Frances Thomas, Brandon S. Willis, Wanjuan Yang, Mark J. O'Connor, Ultan McDermott, Susan E. Critchlow, Lisa Drew, Stephen E. Fawell, Jerome T. Mettetal, Mathew J. Garnett

**Affiliations:** 1 Oncology R&D, AstraZeneca, Cambridge, United Kingdom.; 2 Wellcome Sanger Institute, Cambridge, United Kingdom.; 3 Oncology R&D, AstraZeneca, Waltham, Massachusetts.

## Abstract

Oncology drug combinations can improve therapeutic responses and increase treatment options for patients. The number of possible combinations is vast and responses can be context-specific. Systematic screens can identify clinically relevant, actionable combinations in defined patient subtypes. We present data for 109 anticancer drug combinations from AstraZeneca's oncology small molecule portfolio screened in 755 pan-cancer cell lines. Combinations were screened in a 7 × 7 concentration matrix, with more than 4 million measurements of sensitivity, producing an exceptionally data-rich resource. We implement a new approach using combination Emax (viability effect) and highest single agent (HSA) to assess combination benefit. We designed a clinical translatability workflow to identify combinations with clearly defined patient populations, rationale for tolerability based on tumor type and combination-specific “emergent” biomarkers, and exposures relevant to clinical doses. We describe three actionable combinations in defined cancer types, confirmed *in vitro* and *in vivo*, with a focus on hematologic cancers and apoptotic targets.

**Significance:**

We present the largest cancer drug combination screen published to date with 7 × 7 concentration response matrices for 109 combinations in more than 750 cell lines, complemented by multi-omics predictors of response and identification of “emergent” combination biomarkers. We prioritize hits to optimize clinical translatability, and experimentally validate novel combination hypotheses.

*This article is featured in Selected Articles from This Issue, p. 695*

## Teaser

A screen of 109 anticancer drugs in 755 pan-cancer cell lines, accompanied by a prioritization framework, was conducted, and three examples of high-priority hits that offered a combination benefit were described and confirmed both in *vitro* and *in vivo*.

## INTRODUCTION

Many anticancer agents have limited single agent activity in the clinic, making drug combinations an important treatment strategy. The first successful combination chemotherapy, introduced more than 50 years ago, consisted of a cocktail of four drugs (cyclophosphamide, vincristine, procarbazine, and prednisone) and resulted in durable clinical responses in patients with Hodgkin lymphoma (1, 2). These chemotherapy combinations were often determined empirically in the clinic using existing monotherapy treatments. The recent advent of molecularly targeted agents has led to the development of more rationally designed combinations. Inhibiting multiple nodes in either the same or parallel signaling pathways can help tackle problems such as pathway redundancy, feedback reactivation, and tumor heterogeneity, all of which can contribute to reduced efficacy and disease progression (3). There are, however, several challenges that need to be addressed when identifying efficacious drug combinations. First, obtaining deep pharmacologic profiles of available targeted and chemotherapeutic agents is a complex and resource-intensive task. Second, handling the scale of this data generation and analyses toward identifying “actionable” combinations are difficult. Finally, clinically translatable combinations that deliver patient benefits are rare.

Employment of several criteria into portfolio decisions has resulted in higher success rate during discovery and development of novel drugs (4, 5), including considerations of the target (efficacy), safety, patient population, exposure, and commercial opportunity. Like single-agent drugs, candidate drug combinations require demonstration of activity in a patient population of unmet clinical need, an efficacy profile similar or superior to existing treatments, confidence in the tolerability profile, and knowledge of the exposures required for activity. When possible, application of these principles to design and analysis of *in vitro* combination screens could increase the likelihood of gathering this crucial understanding early in the drug development process. Specifically, comprehensive analysis of multi-omics data for cell line panels can identify potential biomarkers that could lead to patient stratification in the clinic. Avoiding combinations that are broadly active across models in *in vitro* screens can exclude combinations that may also be active in cells without genetic alteration, which thus could have activity in healthy tissues, limiting tolerability. Finally, the design of dose–response surfaces covering a matrix of concentrations relevant to clinical exposures can help inform the right doses for development.

Several groups, including ourselves (6–8), have published unbiased combination screens in cancer cell lines. These have generally focused on assessing large numbers of combination pairs in relatively small cell panels. For example, we published a pan-cancer study as part of the DREAM combination prediction challenge that included >11,500 experiments across 910 combinations in 85 cell lines (6); O'Neil and colleagues published >22,000 experiments across 583 combinations in 39 cell lines (9); and the NCI-ALMANAC study included >5,000 combinations in 60 cell lines of the NCI-60 panel (10). We also recently described screening subsets of 2,025 combinations across 125 cell lines for three cancer types (7). For all these studies, in addition to screening relatively few cell lines, drug combinations were tested using either a limited or a subset of a full concentration matrix, thereby limiting the ability to comprehensively examine the most relevant range. Here, to extend our knowledge of effective drug combinations beyond previous studies, we screened 109 drug combinations using a 7 × 7 concentration matrix across 755 cell lines and developed an end-to-end framework that led to the identification and validation of clinically actionable drug combinations.

## RESULTS

### More than 68,000 Combination: Cell Line Pairs Screened in 41 Cancer Types

**Figure 1.**
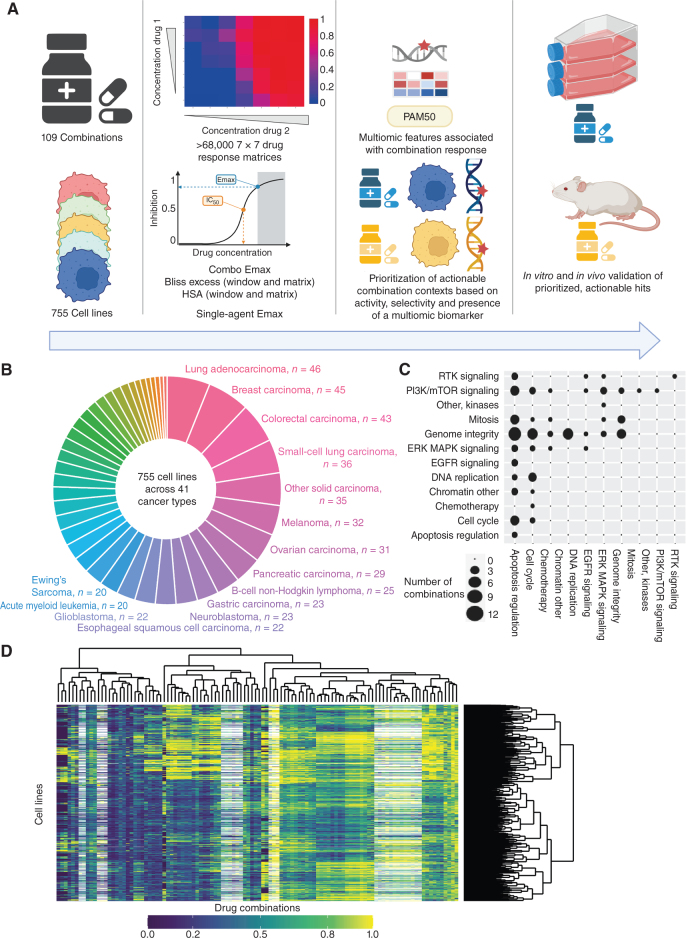
Dose response matrix combination screening landscape. **A,** Schematic of screen and analysis. Created with BioRender.com. **B,** Overview of cell line cancer types. **C,** Drug combinations screened grouped by drug target pathways. **D,** Combo Emax for 755 cell lines screened with 109 combinations. White represents combination/cell line pairs not screened.

We screened a diverse and molecularly characterized panel of 755 cancer cell lines, covering 41 cancer types (ref. 11; Fig. 1A and B; Supplementary Table S1). Against these cell lines, we screened 109 unique combinations of 37 individual drugs and investigational agents, with the majority coming from the AstraZeneca portfolio covering diverse targets and mechanisms of action with a particular focus on compounds targeting genome integrity, apoptosis, and the cell cycle, which had potential for broad activity in a pan-tumor panel and which were of interest for clinical development (Fig. 1C; Supplementary Table S2). Overall, this included 68,816 combination:cell line pairs: 82 combinations were screened in 755 cell lines and additional 27 combinations screened against a half cell line panel of 376 cell lines. To enable in-depth investigations of drug combination responses, we included a high coverage of chosen pathways, including combinations of drugs targeting apoptosis with genome integrity (Fig. 1C).

Drugs were screened in a 7 × 7 combination matrix over a 1,000-fold concentration range of each drug chosen to cover IC_50_ values reported in previous single-agent drug screens (12, 13). The concentrations were informed by clinical relevance, including clinically achievable *C*_max_ (maximum achievable concentration within the body). This design led to a wide range of combination Emax (combo Emax) values (second highest viability effect) obtained across the screen (Fig. 1D). As a control for screen quality, plates had low coefficient of variation, robust dynamic range as measured by Z-factor, and high correlation between control replicate screens (Supplementary Fig. S1A–S1C; see Methods). Furthermore, there was a high correlation when comparing single-agent IC_50_ values for six overlapping drugs screened using the same experimental platform (Supplementary Fig. S1D; ref. 13), the GDSC2 (Genomics of Drug Sensitivity in Cancer 2) dataset (Pearson *R* = 0.855; 735 common cell lines). Comparisons with the GDSC1 and PRISM monotherapy datasets had good [GDSC1 (12); Pearson *R* = 0.753; 699 common cell lines for two drugs] to moderate [PRISM (14); Pearson *R* = 0.513; 346 common cell lines for 13 drugs] correlations, with the lower correlations likely reflecting the use of different experimental platforms and protocols (Supplementary Fig. S1E and S1F). These results support the robustness of the screen.

We used multiple estimates of single agent and combination activity. These include two single-agent IC_50_ values, the two single-agent maximum viability reductions (single-agent Emax; Supplementary Fig. S1G), combination maximum viability reduction (combo Emax, the second highest activity level seen in the matrix; Supplementary Fig. S1H), and synergy scores, according to either the Bliss model (15) or highest single agent (HSA; ref. 16). HSA metric identifies drug combinations if the response is greater than either single agent alone. We report “matrix” synergy scores averaged across all 49 wells of the combination matrix. In addition, we report “window” synergy scores calculated across all 25 possible 3 × 3 submatrices of the 7 × 7 matrix and report the synergy score for the 3 × 3 “window” with the largest synergy score (Supplementary Fig. S1I). The window synergy score is useful where synergy is concentration dependent. For example, IST-MES1 mesothelioma cell line treated with AZD5991 (MCL1 inhibitor) + AZ-3202 (BCL-Xli; also known as compound 15; ref. 17) had higher Bliss synergy excess for the window (0.823) versus matrix (0.180; Supplementary Fig. S1J). Overall, 52.3% of combination:cell line pairs had a negative Bliss matrix excess and positive Bliss window excess, indicating that synergy was frequently observed within a narrow range of tested concentrations. Bliss excess was highly correlated with HSA excess (*R* = 0.924; Supplementary Fig. S1K). In contrast, there was poor correlation between combo Emax with either HSA or Bliss synergy, consistent with some combination activity being driven by single-agent activity (Supplementary Fig. S1L and S1M). Similarly, single-agent Emax weakly correlates with combo Emax (Supplementary Fig. S1N), and single-agent activity poorly correlates with synergy (Supplementary Fig. S1O).

Fitted and raw data are available through Figshare and the GDSC Combination website (https://gdsc-combinations.depmap.sanger.ac.uk/), where data can be visualized and explored at the screen, cancer type, combination, and cell line combination. Altogether, these data are a rich resource and show the value of acquiring multiple estimates of single-drug and combination activity across a matrix of concentrations.

### Prioritization Based on Combination Activity and Tumor Subtypes Specificity

**Figure 2.**
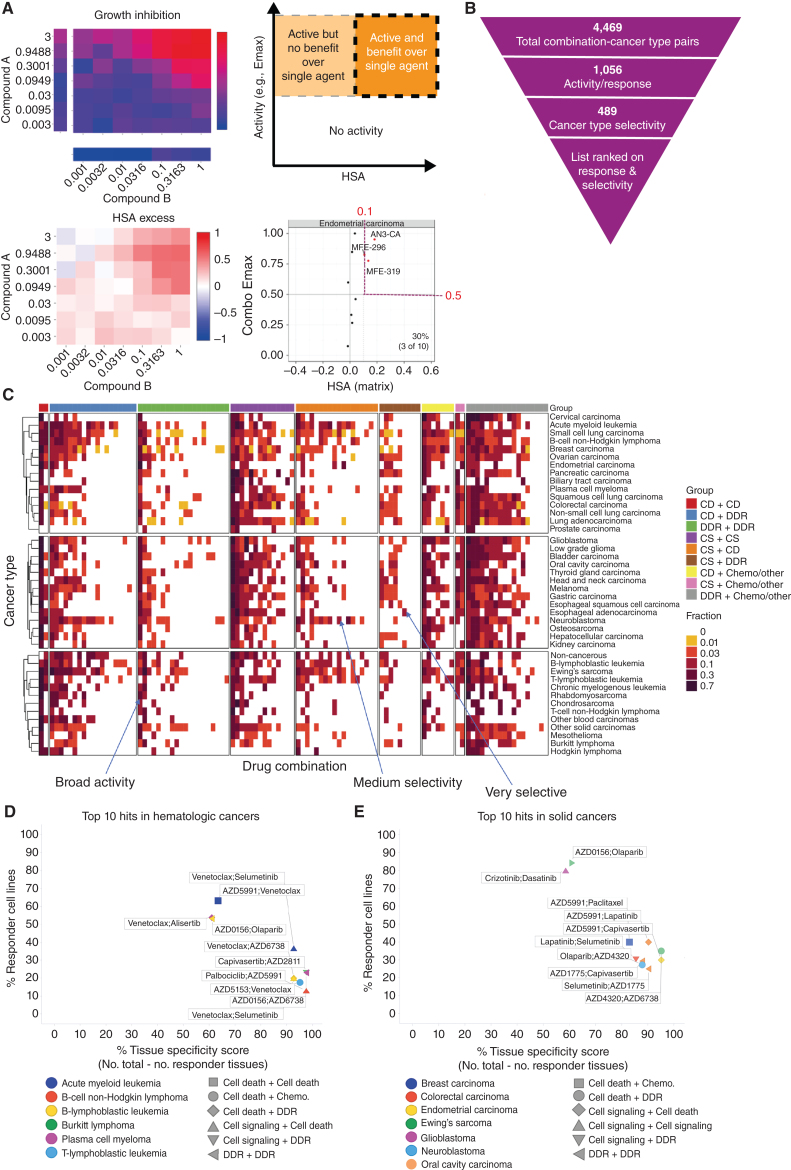
Shortlisting for active and selective combinations. **A,** Growth inhibition (Emax) and HSA matrix plots were generated for each combination in every cell line. Combo Emax and HSA were used to identify active combinations with benefit over single agent. **B,** Combinations were filtered on the basis of their activity and selectivity in the tested cancer types. **C,** Activity of each combination tested in this screen in 41 cancer types. The fraction of cell lines where the combinations are active is indicated and combinations are grouped by category. **D** and **E,** Top 10 hits in hematologic cancers (**D**) and solid tumors (**E**). Percentage of responder cell lines for each combination in each cancer type plotted versus cancer-type specificity scores. Each color represents a cancer type and combination categories are represented by different shapes. CD, cell death; DDR, DNA damage response; CS, cell signaling; chemo, chemotherapeutic agents.

From more than 68,000 combination:cell line pairs tested, we aimed to identify candidate combinations with the greatest potential to be taken forward into clinical development. Therefore, we sought to prioritize combinations with strong activity specifically focused within particular tumor subtypes. As a first step, we identified combination:cell line pairs with high activity (combo Emax > 0.5) and combination benefit/synergy beyond single-agent activity (HSA > 0.1; Fig. 2A). We have previously screened nine combinations with a limited concentration matrix in up to 114 breast, colon, and pancreatic cancer cell lines, representing 4,790 overlapping combination:cell line pairs (7). In support of our screening results, the classification of high activity and synergy beyond single-agent activity agreed with the classification of synergy/nonsynergy for 65.4% of combination:cell line pairs (Supplementary Fig. S2A). More examples of active combinations were identified in this study, supporting the value of our approach incorporating a 7 × 7 concentration matrix design and HSA metric.

We next identified combinations where at least 10% of cell lines tested within a specific cancer type fulfilled these criteria, reducing the number of combination:cancer type pairs by 76% from 4,469 to 1,056 (Fig. 2B; Supplementary Tables S3 and S4). The minimum threshold of 10% was chosen to allow a relatively small number of models to highlight a potential combination, while maintaining a strong enough signal to support clinical actionability. Six combinations showed no combination benefit (HSA < 0.1, combo Emax < 0.5) in the 755 cell lines tested, including the MCL1 inhibitor AZD5991 combined with either the DNAPK inhibitor AZD7648 or cMET inhibitor savolitinib; ATM inhibitor AZD1390 combined with either the MEK inhibitor selumetinib, the EGFR inhibitor gefitinib, or the AKT inhibitor capivasertib; and the PARP inhibitor olaparib combined with the BRD4 inhibitor AZD5153. Combination benefit was most enriched in combination:cell line pairs where the combination targeted ERK/MAPK and PI3K/MTOR signaling, or dual targeting of the cell cycle (Supplementary Fig. S2B).

The majority of the active combinations were active in multiple cancer types. Specifically, nineteen combinations were active in more than 50% of cancer types (Supplementary Table S5). Of these, five involved combinations targeting proteins that have a protein–protein/functional interaction from STRING database (prexasertib + AZD1775, SRA737 + AZD1775, AZD5991 + AZ3202, trametinib + taselisib, dasatinib + trametinib), and three combinations target synthetic lethal pairs of genes as defined by SynLethDB (AZD5153 + selumetinib, trametinib + taselisib, AZD5991 + AZ-3202; Supplementary Fig. S2C). Broadly active combinations may be more likely to be active in normal tissues, thereby limiting their therapeutic window and potential for clinical development. For example, the combination of the MEK inhibitor, selumetinib, and the AKT inhibitor, capivasertib, had activity in 22 of 41 cancer types. Despite strong preclinical activity here and in other studies, the overlapping clinical toxicities of selumetinib (inhibitor of MEK: MEKi) combined with MK2206 (AKTi) was found to prevent sufficient dose escalation to achieve the desired level of target inhibition, and clinical activity was not observed (18). However, more recent AKT inhibitors, such as capivasertib or ipatasertib, may have a broader therapeutic window on account of an ATP-competitive mode of action, whereas MK2206 was an allosteric inhibitor. To maximize the therapeutic window of selected combinations, an additional filtering step was therefore applied to select combinations with high activity (HSA > 0.1 and combo Emax > 0.5) in less than 50% of cancer types. This reduced the number of combination:cancer type pairs to 489 (Fig. 2B and C; Supplementary Table S6).

As a final step of prioritization, drug combinations were ranked on the basis of their activity (% responders in a cancer type) and cancer type selectivity (cancer type specificity score). The cancer type specificity score was calculated by subtracting the number of cancer types showing sensitivity to an individual drug combination (at least 10% responder cell lines) from the total number of cancer types. Activity and cancer-type selectivity were given equal weights and scores were given as a sum of percentage responders in that particular cancer type and the cancer type specificity score. Combination:cancer type pairs tested in less than 10 cell lines were excluded to prevent small sample sizes biasing the analysis, leading to a list of 99 combination:cancer type pairs in hematologic cancers (Supplementary Table S7) and 252 combination:cancer type pairs in solid tumors. The top 100 combination:cancer type pairs are shown in Supplementary Table S8. This systematic approach informed our prioritized shortlist for prospective validation (see validation section below).

The top scoring combination in hematologic cancers was selumetinib (MEKi) + venetoclax (BCL2i) in acute myeloid leukemia (AML; response in 36% of AML cell lines), which also had above 10% activity in B-Lymphoblastic Leukemia (Fig. 2D; Supplementary Tables S6 and S7). Another highly ranked combination also included venetoclax, now with a cell death agent AZD5991 (MCL1i), with 63% responder cell lines in AML, but less selectivity across cancer types (active in 15 cancer types). For solid tumors, crizotinib + dasatinib in low-grade glioma (87% responders; active in 16 cancer types) and AZD0156 (ATM/ATRi) plus olaparib (PARPi) in Ewing's sarcoma (84% responders; active in 16 cancer types) were high scoring (Fig. 2E; Supplementary Table S8). Several combination:cancer type pairs that have previously been shown to drive combination activity were identified, including AZD5991 (MCLi) + venetoclax (BCL2i) that was active in AML cell lines, and has been tested in a phase II trial in patients with AML (NCT03218683), providing support that our screen and prioritization process is capable of identifying clinically relevant combinations in specific cancer types.

To evaluate the impact of our scoring thresholds on prioritized combinations, we investigated alternative thresholds for percentage response and cancer type specificity. Higher response thresholds (from more than 10% of cell lines to 25%, 50%, or 75%) led to a drop in the number of top hits (120, 14, and 3 respectively) by excluding combinations which are most cell line selective in their activity (Supplementary Tables S9 and S10). There was no change in the top 15 hits when the percentage response threshold was increased to 25% (Supplementary Table S9). Changing the cancer type specificity threshold (from less than 50% of cancer types to either less than 25% or 75%) also altered the number of combinations (223 and 727, respectively), either requiring combinations to be highly cancer type specific, or including widely active combinations which are less likely to be clinically tolerable.

With respect to the frequency of active combinations by cancer type, AML had the highest number of active combinations within the top hematologic cancer hits (25 combinations), followed by chronic myelogenous leukemia (17 combinations) and B lymphoblastic leukemia (15 combinations; Supplementary Table S7). In solid tumors, the highest number of active combinations within the top 100 hits were in Ewing's sarcoma (16 combinations), followed by head and neck (9 combinations), and small-cell lung carcinoma (8 combinations; Supplementary Table S8).

To gain mechanistic insights into the top ranked combinations, we assigned combinations into nine categories based on the mechanism of action of the two constituent drugs (Supplementary Table S11). Out of the top 100 drug combination:cancer type pairs in solid tumors, 19 hits belonged to the “cell death” plus “cell signaling.” However, in hematologic cancer top hits, the highest number of combinations were the ones targeting “cell death” plus “DNA damage response (DDR)” pathways (31 hits). In contrast, combinations from the “cell signaling” plus “chemotherapeutic agent” category were overall rare (2 hits for hematologic cancer and 2 hits for solid tumors; Supplementary Tables S7, S8, and S12). Out of the top ten drug combinations in hematologic cancer, 7 included at least one compound targeting the “cell death” pathway (Fig. 2D). This finding is in agreement with the fact that apoptosis/cell death pathways are frequently dysregulated in hematologic cancers leading to efficacy of cell death agents in these tumors (19). Overall, our prioritization approach enriched for combinations that are selectively active in subsets of cell lines and by tumor type, increasing the probability of identifying combinations with a clinically manageable tolerability profile.

### Multiomics Analysis Identifies Biomarkers of Combination Response

**Figure 3.**
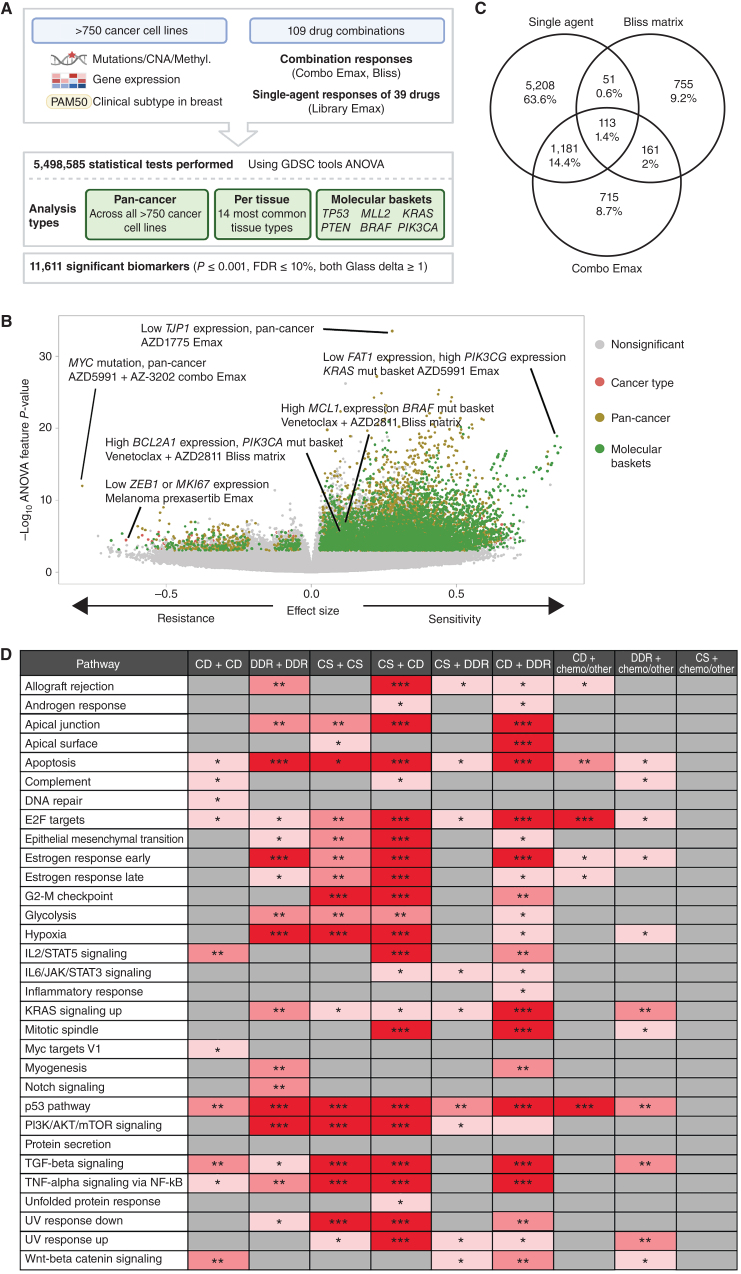
Multi-omics biomarkers of combination activity. **A,** Schematic of biomarker pipeline including molecular features incorporated and analyses performed. Created with BioRender.com. **B,** Volcano plot of biomarkers from all analyses. Statistically significant associations are colored by analysis type, nonsignificant biomarkers are colored gray. **C,** Venn diagrams of the biomarkers from different inputs leading to the identification of emergent biomarkers. Note that single-agent biomarkers may be duplicated for the multiple combinations in which the single agent has been screened: the Venn diagram depicts unique single-agent biomarker associations only. **D,** Significant enriched pathways for emergent biomarkers in each drug combination category based on *P*_adj_ values. * 0.05 < *P* < 0.01, ** 0.01 < *P* < 0.001, *** *P* < 0.001. CD, cell death; CS, cell signaling; chemo, chemotherapeutic agents.

We leveraged the large number of cell lines screened to understand how molecular context affects drug combination response. Using GDSCtools ANOVA (20), we performed 5.4 million statistical tests to identify statistically significant associations between drug response metrics and multi-omics features (Fig. 3A; Supplementary Table S13). This included curated molecular features previously associated with single-agent drug response (somatic mutations, copy-number alterations (CNA), and DNA methylation; *n* = 1,073; ref. 13); additional molecular features curated from public datasets (see Methods; *n* = 586); a curated set of binarized gene expression features (*n* = 1,344; refs. 21, 22); and PAM50 status for breast cancer cell lines (*n* = 9; refs. 23, 24). Associations were identified with five response metrics, including single agent (compound 1 Emax, compound 2 Emax) and combination responses (combo Emax, Bliss matrix, Bliss window). Bliss was chosen over HSA as the synergy metric for biomarker identification because it is a more stringent measure of drug combination response and biomarkers of Bliss scores are more likely to identify drug interactions.

We identified significant associations for 21 different subgroups of cell lines. This included pan-cancer across the entire cell line panel; per cancer type for the 14 most common cancer types in our panel (>19 cell lines); and 6 molecular “baskets” representing cancer type–agnostic cell line subpanels of the six most frequently mutated genes [*TP53* (*n* = 477 cell lines), *KRAS* (*n* = 107), *MLL2/KMT2D* (*n* = 81), *PTEN* (*n* = 72), *PIK3CA* (*n* = 80), and *BRAF* (*n* = 61)]. Overall, we identified 11,611 statistically significant associations (*P* ≤ 0.001, FDR ≤ 10%, and positive and negative Glass delta ≥1; Fig. 3A and B). This included 6,911 nonunique single-agent and 4,700 combination biomarkers, representing at least one significant association for every combination tested (combo Emax associations, *n* = 2,170; Bliss matrix, *n* = 1,080; Bliss window, *n* = 1,450). Cancer type–specific ANOVAs and molecular basket ANOVAs identified 803 and 4,388 context-specific biomarkers, respectively, in addition to those found in the pan-cancer setting, confirming the benefit of considering sensitivity biomarkers in specific molecular contexts (Supplementary Figure S3A and S3B; ref. 7).

A subset of biomarker associations were linked to the target of one or both of the drugs in a combination. For example, elevated expression of *PIK3CG* was associated with a greater Bliss window synergy score for AZD8186 (PI3Kβi) + palbociclib (CDK4/6i) in the *KRAS* molecular basket (Supplementary Fig. S3C). Elevated expression of *BIM* (BCL2L11) was significantly associated with higher AZD4320 (BCL2i,BCL-XLi) and venetoclax (BCL2i) single-agent Emax values in the *KRAS* molecular basket (Supplementary Fig. S3D and S3E), and elevated *BCL2* expression was associated with higher venetoclax (BCL2i) Emax in the *TP53*-mutant basket (Supplementary Fig. S3F). In many instances, combination biomarkers were also associated with the single-agent activity of a constituent drug. Specifically, 59.5% (6,911 of 11,611) of combination biomarkers were also biomarkers for at least one of the two monotherapies in that combination. This has been observed previously; for example, *BRAF* mutation is a predictor of dabrafenib monotherapy activity in multiple cancer types and of response to dabrafenib-containing combinations in colon cancer (25–27).

We reasoned biomarkers specifically associated with combinatorial activity, and not with single-agent activity of the individual constituent drugs, so called “emergent” combination biomarkers, would be of particular interest because they are more likely to capture properties arising from drug–drug interactions. By excluding monotherapy-driven markers for each compound, we identified 14% of biomarkers (1,631 of 11,611: 755 Bliss matrix only, 161 Bliss matrix and combo Emax, 715 combo Emax only; Fig. 3C) as “emergent” (Supplementary Table S13). Considering the top 100 most statistically significant emergent biomarkers (Supplementary Table S14), these involved ten unique combinations, seven of which included BCL2 or MCL1 inhibitors that target cell death pathways (Supplementary Tables S15 and S16 includes emergent biomarkers). To gain further insights into their properties, emergent biomarkers were grouped by signaling pathway prior to performing pathway enrichment analysis using EnrichR comparing single-agent and emergent biomarkers for each mechanistic category of combination (ref. 28; Fig. 3D; Supplementary Fig. S3G). Apoptosis, P53, and E2F pathway were among the most enriched pathways across the combination categories. A smaller number of pathways were enriched in categories including chemotherapeutic agents, with no signaling pathway being enriched for the emergent biomarkers in the “cell signaling” (non-DDR/cell death) plus chemotherapy category. Chemotherapeutics were broadly active in our screen, likely explaining why highly predictive markers of response were not observed. We hypothesize that these emergent biomarkers represent predictors of drug–drug interactions that cannot be readily identified from single-agent activity alone, and thus highlight the potential utility of combination screens for biomarker identification over using monotherapy biomarkers alone.

### Combination Validation

We identified active and cancer-type selective combinations using our prioritization framework. A subset of the top scoring combinations are exemplified here and were validated *in vitro* and *in vivo* based on AstraZeneca portfolio interest, prior knowledge, and to illustrate different types of therapeutic opportunities including new combinations and repurposing.

#### AZD5991 Plus Venetoclax in AML

The second ranked combination in hematologic cancers was venetoclax (BCL2i) + AZD5991 (MCLi) in acute myeloid leukemia (AML). For 13 of 19 AML cell lines, the combination was active (HSA > 0.1 and combo Emax > 0.5; Supplementary Fig. S4A and S4B). The combination was also active in other hematologic cancers including Hodgkin lymphoma (42.9%, 3 of 7 cell lines), B-lymphoblastic leukemia (40%, 6 of 15 cell lines) and B cell non-Hodgkin lymphoma (36%, 9 of 25 cell lines), as well for some solid tumors such as small cell lung carcinoma (47.2%, 17 of 36 cell lines) and Ewing's sarcoma (45%, 9 of 20 cell lines; Supplementary Fig. S4C). From our biomarker analysis, cell-cycle and DNA repair pathways genes (e.g., *BRCA2, WEE1, CDC25A*) were associated with combo Emax, and downregulation of the nucleotide excision repair protein ERCC1 was associated with Bliss synergy, providing a putative mechanistic link between combination activity and DDR and cell cycle–related pathways, as previously reported (refs. 29–31; Supplementary Fig. S4D; Supplementary Table S17). Importantly, this combination was selective, with 40% (17/41) of tumor types having a response rate >10% and only 19% (8/41) of tumor types having a response rate >25%. In comparison, the combination of AZD5991 with another cell death target, the Bcl-xL inhibitor (AZ-3202), has poor selectivity with a >25% response rate in 90% (38/41) of tumor types (Supplementary Fig. S5A). The combination of AZD5991 + venetoclax was under clinical investigation in a phase I/IIa trial in patients with refractory or relapsed hematologic malignancies but was recently terminated for undisclosed reasons (NCT03218683).

#### Selumetinib Plus Venetoclax or AZD5991 in AML

**Figure 4.**
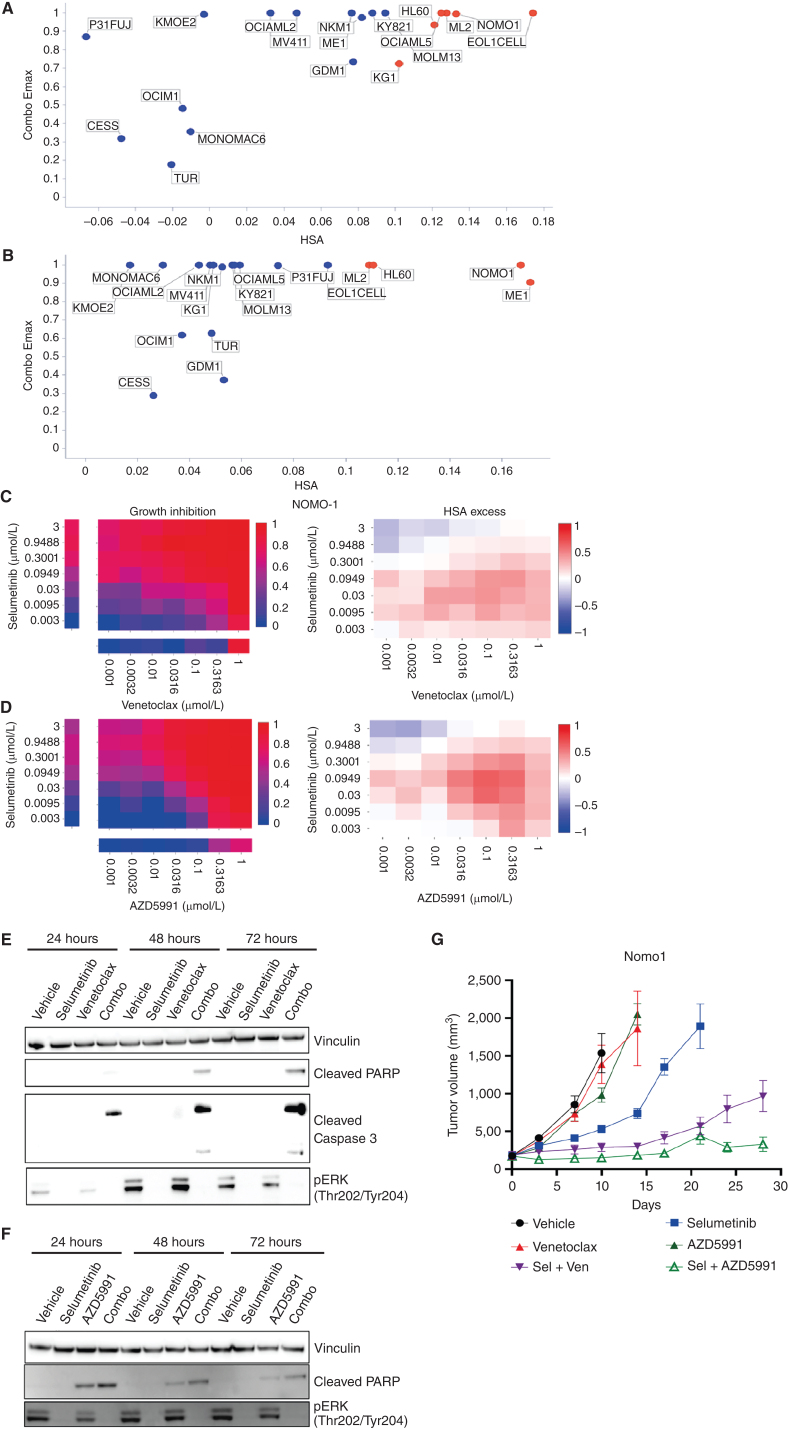
Combination activity of selumetinib plus venetoclax or AZD5991 in AML. **A** and **B,** Combo Emax versus HSA scores in 19 AML cell lines exposed to selumetinib combined with (a) venetoclax or (b) AZD5991. **C** and **D,** NOMO1 growth inhibition and HSA excess to the combination of selumetinib with (c) venetoclax or (d) AZD5991. **E** and **F,** Western blot analysis for apoptosis markers in NOMO1 cells following time course treatment with selumetinib (300 nmol/L) combined with (e) venetoclax (300 nmol/L) or (f) AZD5991 (100 nmol/L). **G,** Tumor growth in NOMO1 xenografts treated with selumetinib, AZD5991, or venetoclax alone or in combination for 28 days (*n* = 5 each arm). Control and monotherapy experimental arms were halted once the maximum permitted tumor volume (2,000 cm^3^) was reached. Data are plotted as mean tumor volume ± SEM.

Two further effective and specific combinations in AML were selumetinib (MEK1/2i) combined with venetoclax (BCL2i) or AZD5991 (MCLi; Supplementary Table S7). Both combinations also had activity in other hematologic cancers and solid tumors (Supplementary Fig. S6A and S6B). Of the nineteen AML cell lines included in the screen, six (EoL-1-cell, ML-2, OCI-AML5, NOMO-1, KG-1, HL-60) had strong combination activity when selumetinib was combined with venetoclax and four cell lines (ME-1, ML-2, NOMO-1, HL-60) when combined with AZD5991 (Fig. 4A–D). These cell lines predominantly harbored alterations in MAPK pathway family members including OCI-AML5 (*SOS1*^N2337Y^, *NF1*^K1385R^), ML-2 (*KRAS*^A146T^), HL-60 (*NRAS*^Q61L^), and Nomo-1 (*KRAS*^G13D^). Consistent with these findings, among the significant biomarkers for venetoclax and selumetinib were proteins involved in ERK-MAPK signaling including EGF and SOS1, as well as members of the BCL2 family (PMAIP1; Supplementary Figure S7A and S7B; Supplementary Table S17).

To validate these two combinations *in vitro*, we assessed MAPK pathway inhibition and induction of apoptosis in the sensitive Nomo-1 cell line. Treatment with selumetinib completely inhibited phospho-ERK (pERK) levels tested for up to 72 hours. Neither venetoclax nor AZD5991 altered pERK levels (Fig. 4E and F). When selumetinib was combined with venetoclax or AZD5991, pERK suppression was maintained and induction of cleaved PARP and cleaved caspase 3 was observed as early as 24 hours, increasing at 72 hours. AZD5991 alone caused a weak induction of cleaved PARP which was enhanced by the combination. Combination benefit was also achieved by combining selumetinib with an alternative selective MCL1 inhibitor, tapotoclax, or selective BCL2 inhibitor, S55746 (both currently under clinical investigation) in NOMO1, HL60, and ML2 cell lines. Similarly, an alternative MEK1/2 inhibitor trametinib was active in combination with the four MCL1 and BCL2 inhibitors tested (Supplementary Figures S8A–S8C and S9A–S9C). Together, these results confirm on-target combination activity induces suppression of MAPK signaling and increased apoptosis.

We next evaluated the *in vivo* activity of the combinations using subcutaneous Nomo-1 xenograft models. Neither venetoclax (100 mg/kg oral daily) nor AZD5991 (two intravenous doses of 30 mg/kg given two hours apart once weekly) alone caused any significant tumor growth inhibition when dosed as a monotherapy (Fig. 4G). While selumetinib monotherapy (10 mg/kg oral twice daily, 8 hours apart) led to 63% tumor growth inhibition (TGI) at day 10, tumors eventually grew out. Notably, combining selumetinib with venetoclax or AZD5991 markedly reduced tumor growth. Tumors treated with the selumetinib + venetoclax combination only reached a mean tumor volume of 963 mm^3^ after 28 days. The combination of selumetinib with AZD5991 was even more pronounced (88% TGI at day 10) and the mean tumor volume had not exceeded 400 mm^3^ by day 28. Collectively, these results confirm the *in vitro* and *in vivo* efficacy of these combinations in the setting of AML.

Venetoclax monotherapy in AML is only modestly active and significant benefit comes from addition of a second agent such as decitabine or cytarabine. Selumetinib has modest clinical activity as a monotherapy in patients with AML (32). Given that the MAPK pathway is activated in about 70% of patients with AML due to mutations in upstream key proteins including RAS and FLT3 (33), and recent studies which show that further mutations in MAPK can arise from use of venetoclax or targeted therapies like gilteritinib (FLT3i), the use of a MEK inhibitor like selumetinib as a combination partner has strong rationale (34). Furthermore, the activity of selumetinib combined with AZD5991 also suggests an alternative partner in patients where BCL2 inhibition is insufficient to remove the antiapoptotic blockade, and combination with an MCL1 inhibitor may be a good choice.

#### AZD2811 Plus Venetoclax in DLBCL

An additional highly ranked combination was the aurora kinase B inhibitor (AurkB) AZD2811 + venetoclax in B-cell non-Hodgkin lymphoma (NHL). The active pharmaceutical ingredient in AZD2811 (AZD1152) has previously undergone clinical evaluation for diffuse large B-cell lymphoma (DLBCL), and the combination activity of aurora kinase B inhibitors and BH3 mimetics has been investigated in solid and hematologic malignancies (35). AZD2811 + venetoclax has efficacy in *TP53*-mutant and wild-type AML *in vitro* and *in vivo* models, and overcame venetoclax resistance in *TP53* models (36). However, despite these preclinical and clinical signals, the combination of aurora kinase inhibitors with BCL2 inhibitors has not been reported to be active in DLBCL.

**Figure 5.**
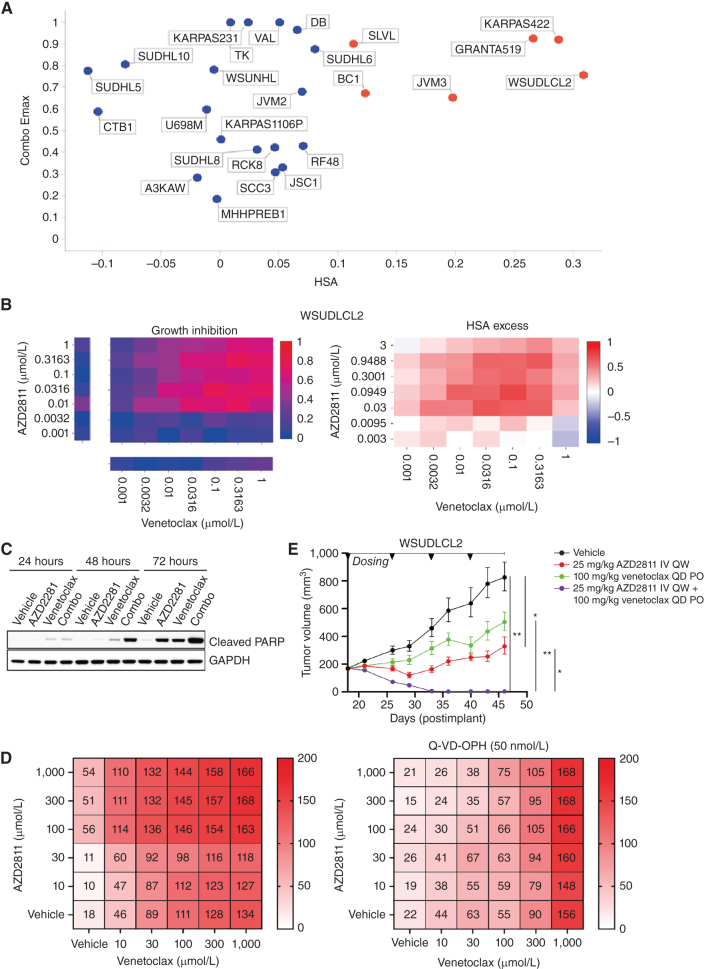
AZD2811 plus venetoclax combination in DLBCL. **A,** Combo Emax versus HSA in 25 B-cell NHL cell lines including 11 DLBCL cell lines. Cell lines with high combination activity (combo Emax > 0.5 and HSA > 0.1) are in red. **B,** Growth inhibition and HSA excess matrices in DLBCL cell line WSUDLCL2. **C,** Western blot analysis for cleaved PARP in WSUDLCL2 cells treated with AZD2811 or venetoclax alone or in combination. **D,** Matrix plots indicating combination activity (measured by growth inhibition) in WSUDLCL2 cells pretreated with pan caspase inhibitor Q-VD-OPH and exposed to AZD2811 combined with venetoclax for 72 hours. Matrix values represent cell viability normalized to day 0 on the scale of 0 to 200 (value < 100 = percentage of growth inhibition, value > 100 = cell death). **E,** Tumor growth in WSUDLCL2 xenografts treated with AZD2811 or venetoclax alone or in combination for 46 days (*n* = 6 per group, * 0.05 < *P* < 0.01, ** 0.01 < *P* < 0.001). Data are plotted as mean tumor volume ± SEM. PO, orally; QD, every day; QW, every week.

In our screen, 6 of the 25 B-Cell NHL cell lines had strong combination activity (HSA > 0.1 and combo Emax > 0.5), including 2 DLBCL cell lines (WSU-DLCL2 and KARPAS_422; Fig. 5A and B). Combination activity was also seen in AML (5 of 19 cell lines), Ewing's sarcoma (4 of 20 cell lines), plasma cell myeloma (3 of 13 cell lines), and small-cell lung carcinoma (9 of 36 cell lines; Supplementary Fig. S10A). In support of our screening results, combinations with alternative compounds targeting aurora kinase (danusertib) and BCL2 (S55748) had combination benefit in DLBCL models WSU-DLCL2 and KARPAS422 (Supplementary Fig. S11A and S11B). Except for upregulation of *CCNB1*, *MCL1*, and *BCL2A1* gene expression in the *KRAS*, *BRAF*, and *PIK3CA* baskets, respectively, other significant biomarkers were noncanonical to the cell-cycle and cell death pathways that are the targets of the drugs (Supplementary Fig. S12A; Supplementary Table S17).

One of the responsive DLBCL cell lines, WSU-DLCL2, was selected for further *in vitro* and *in vivo* validation. The combination of venetoclax plus AZD2811 led to a time-dependent induction of apoptosis compared with either single agent alone (Fig. 5C), and combination activity was suppressed by pretreatment of cells with the pan-caspase inhibitor Q-VD-OPH (50 nmol/L; QVD; Fig. 5D). In addition, *in vivo* antitumor activity of AZD2811 combined with venetoclax was assessed in mice bearing WSU-DLCL2luc xenografts. Once weekly intravenous administration of 25 mg/kg AZD2811 resulted in a statistically significant tumor growth inhibition (TGI) of 74%, while daily 100 mg/kg venetoclax resulted in 49% TGI but failed to reach statistical significance (Fig. 5E). While both monotherapies were unable to prevent progressive tumor growth, the combination of AZD2811 and venetoclax drove striking activity, leading to tumor regression resulting in statistically significant complete regression (98% regression) by the third week of dosing. Together, these studies support the *in vitro* and *in vivo* activity of venetoclax + AZD2811 in the setting of DLBCL.

#### Capivasertib (AZD5363) Plus AZD5991 in Endometrial Cancer

**Figure 6.**
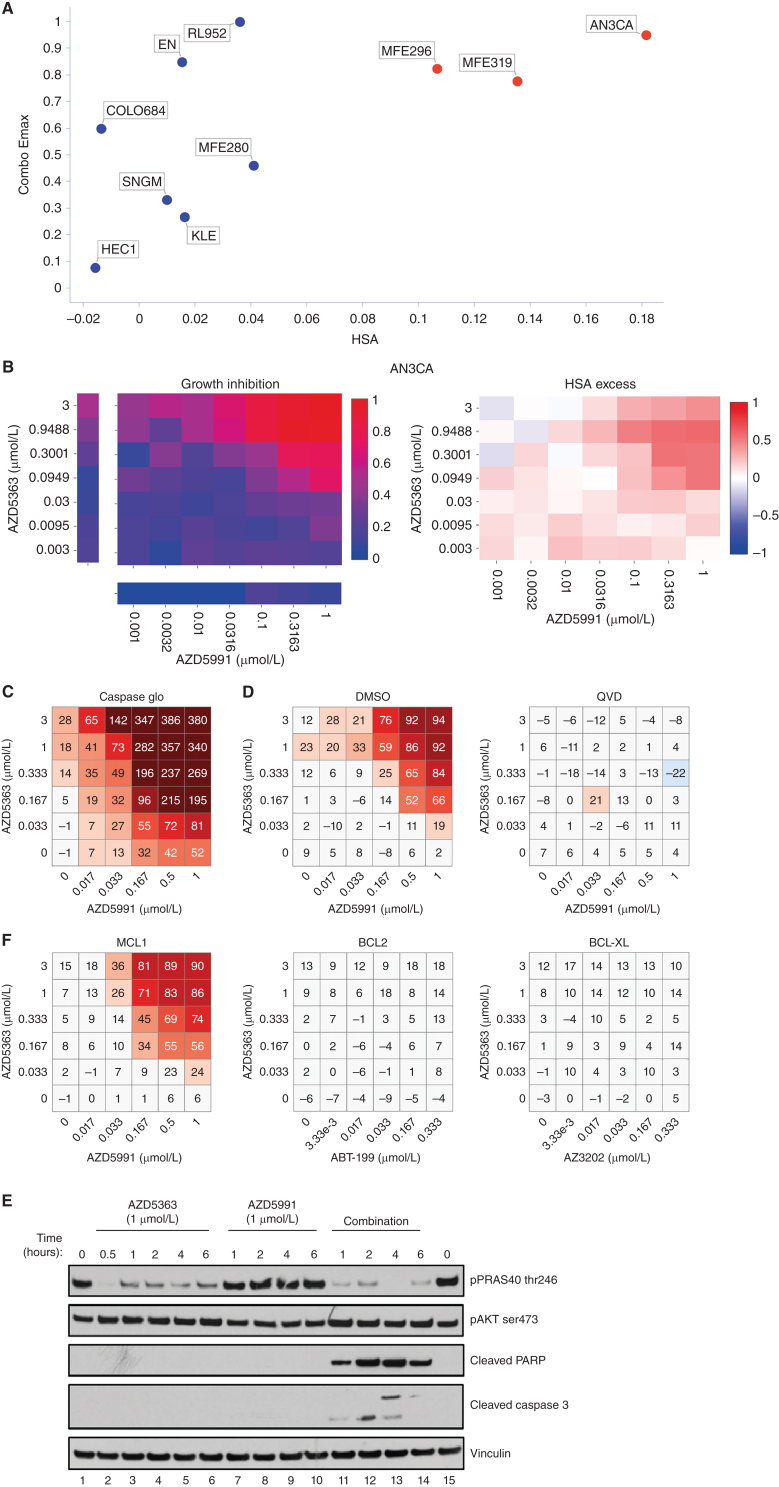
Capivasertib (AZD5363) plus AZD5991 combination activity in endometrial cell lines. **A,** Screening results of combo Emax versus HSA in endometrial cell lines treated with AZD5363 plus AZD5991. Cell lines with high combination activity are in red. **B,** Representative growth inhibition and HSA excess matrix plots in endometrial AN3CA cells. **C,** Matrix plot measuring apoptosis with AZD5991 and AZD5363 at indicated doses for 6 hours in AN3-CA cells. **D,** Matrix plots showing viability for AN3-CA cells pretreated with DMSO or QVD (caspase inhibitor) for 16 hours prior to the combination for 6 hours. **E,** Western blot analysis in AN3-CA cells treated with AZD5363 (1 μmol/L), AZD5991 (500 nmol/L), or in combination at indicated times. **F,** Matrix plots showing viability in AN3-CA cells treated with AZD5991 or venetoclax (ABT-199 -BCL2 inhibitor), AZD4320 or AZ3202 (BCL-XL inhibitors) with AZD5363 at indicated doses for 6 hours.

The combination of the AKT inhibitor capivasertib (AZD5363) with the MCL1 inhibitor AZD5991 was one of the most selective combinations, active in only 2 of 41 cancer types (Supplementary Fig. S12B). The greatest responses were in endometrial cancer with 3 of the 10 endometrial cell lines showing strong combination activity (Fig. 6A and B). Our biomarker analysis identified three significant associations involving DDR pathway genes (upregulation of *BRCA2, RAD51* and downregulation of *ERCC1*), and upregulation of genes which directly or indirectly activate AKT (e.g., *CDC25A* in the *TP53* basket and *RHOA* in the *PTEN* basket) were associated with combo Emax and Bliss score (Supplementary Table S17).

We chose two responder cell lines (AN3-CA and MFE-296) and two nonresponder cell lines (HEC1 and MFE-280) for further validation. Both cell lines sensitive to the combination have *PTEN* mutations and had elevated baseline levels of phosphorylated AKT and PRAS40 (Supplementary Fig. S13A). Selective combination activity was confirmed in responsive and non-responsive lines, and notably became apparent as early as 3 hours, before either compound had single-agent activity (Supplementary Fig. S13B and S13C). The combination of capivasertib and AZD5991 led to apoptosis (Fig. 6C–E) as evidenced by induction of cleaved PARP and cleaved caspase 3 as early as 1 hour, as well as a marked induction of caspases (Fig. 6C). Pretreatment with the pan-caspase inhibitor QVD blunted apoptosis (Fig. 6D).

To evaluate the on-target mechanism of action of the combination, we tested alternative compounds with similar target specificity. Combination activity was specific to MCL1 inhibition as both AZD5991 and tapotoclax (an alternative MCL1 inhibitor) showed combination benefit with AKT inhibition in responder cells (Supplementary Fig. S14A and S14B), whereas, the BCL2 inhibitor venetoclax (ABT-199) or a BCL-XL–selective inhibitor AZ-3202 did not (Fig. 6F). The combination effect with AZD5991 also occurred with the AKT inhibitors MK2206 and ipatasertib, as well as AZD8186 (PI3Kβ/δ), and to a lesser degree BYL719 (PI3Kα), but not the mTOR1 inhibitor rapamycin (Supplementary Figs. S14A and S14B and S15). In addition, genetic knockdown of MCL1 in AN3-CA and MFE-296 cells caused a shift and reduction in the IC_50_ of AZD5363 and ipatasertib (Supplementary Fig. S16A–S16D). Taken together, these results show marked combination activity through dual targeting the PI3K–AKT pathway and MCL1 signaling axes in the setting of endometrial cancer.

## DISCUSSION

In this study, 755 genomically characterized cell lines from 41 cancer types were screened with 109 drug combinations using a 7 × 7 concentration matrix to generate more than 4 million individual sensitivity measurements, of which more than 2.3 million describe combination response. Previous studies have been limited to a maximum of 125 cell lines (7) and consequently lack the same diversity of molecular backgrounds and cancer types, which are known to impact treatment response. Furthermore, the use of full dose matrices uniquely provides an opportunity to identify effective combinations across a range of clinically relevant concentrations with enhanced sensitivity compared with previous studies which have adopted a partial matrix approach (6–8, 37). We anticipate that this dataset will be a rich resource and contribute to datasets available for these cancer cell lines as part of a Cancer Dependency Map (38).

Future analyses investigating combinations not prioritized in this study may yield additional actionable combinations, and the data availability will enable this. For example, combinations with activity limited to a small number of cell lines could have utility for a subset of patients if highly predictive markers could be identified. Similarly, combinations which were highly active across multiple tumor types, and so likely to be less tumor cell selective, may be tolerable for patients through the use of fractionated and alternative dosing schedules.

We capitalized on the availability of multi-omics data across all cell lines to not only identify biomarkers within a molecular basket (i.e., clinically relevant genotypes), but also markers of monotherapy and combination response. We report emergent combination biomarkers that could not be readily explained by markers of response to the individual drugs. Such biomarkers, subject to validation, could provide insights into novel biology and signaling pathways driving combination efficacy. Future work incorporating newly available “omics” data such as proteomics (39, 40) may yield additional markers for combination opportunities and enable precision medicine approaches.

Our approach is designed to optimize preclinical interpretation with a focus on actionability. Rather than simply selecting combinations which elicited the greatest synergy, we shortlisted combinations that were highly active, more effective than monotherapy alone, and cancer-type selective. Several of the identified combination hits have already undergone clinical and preclinical development in the same cancer type as identified in our screen, such as the combination of the AZD5991 (MCL1i) + venetoclax (BCL2i) in AML (41). A factor driving our selection of combinations for experimental follow-up was having a rationale for at least one of the agents in the indication. For example, selumetinib (MEKi) + AZD5991 (MCL1i) in AML cell lines, which builds on reports showing combination activity in colorectal and melanoma cell models (30). The combination of AZD2811 (AurkBi) + venetoclax (BCL2i) was shown here to be active in DLBCL cell lines. This combination has activity in AML preclinical models (36). In addition, the combination of capivasertib (AKTi) + AZD5991 (MCL1i) has shown activity in breast cancer models (42) and was identified here as an active combination in endometrial lines. The PI3K/AKT/mTOR signaling pathway is frequently altered in endometrial cancer, therefore capivasertib + AZD5991 could represent an active and potent combination in this cancer type that currently lacks effective treatments. For multiple combinations we confirmed comparable activity using alternative inhibitors to the same targets, indicating that “on-target” combination activity can be achieved with inhibitors in addition to the specific molecules tested here.

Future work should seek to refine and extend the current study. Focused screens in healthy or primary cells, albeit technically challenging, could control for potential combination toxicity. Similarly, tumor xenograft studies should be used to assess *in vivo* activity and tolerability. The inclusion of tumor stroma into screens could inform how the tumor microenvironment modulates combination response, and reveal new active combinations that target tumor cell–stroma signaling. A longer duration of combination exposure to cells might identify combinations that are dependent on cell division, and screening in 3D cultures could reveal combinations dependent on cell–cell interactions and 3D structure. Furthermore, many current clinically effective oncology drug combinations work through targeting tumor heterogeneity, a concept called independent action (43). The large heterogeneous panel of cell lines used here should enable analyses for independent drug action. Beyond the specific combinations identified, we anticipate that the experimental and analytic approach taken here will facilitate the interpretation of future drug combination studies. The richness of the full matrix design should enable data-driven approaches to better model combinatorial drug responses and to guide more efficient experimental designs based on optimized matrices, for example, through subsampling the matrix or discontinuous dosing gradients (7, 44). Furthermore, our study will be of interest to the fields of machine learning and computational biology, and as such complements previously published drug combination studies (6, 9, 37, 45–47).

In conclusion, this study provides a rich resource and identifies actionable combinations as a starting point toward achieving the goal of developing rational combinations to improve treatment options for patients.

## METHODS

### Cell Lines

The majority of cell lines were sourced commercially from repositories and cell banks. To facilitate high throughput screening all cell lines were maintained and screened in one of two media types; DMEM/F12 or RPMI supplemented with 10% FBS, penicillin–streptomycin and sodium pyruvate. All cell line stocks used for screening were tested for *Mycoplasma* contamination prior to banking using both a PCR (EZ-PCR Mycoplasma Detection Kit, Biological Industries) and a biochemical test (MycoAlert, Lonza). Cultures testing positive using either method were removed from the collection.

To prevent cross-contamination or misidentification, all banked cryovials of cell lines were analyzed using a panel of 94 single-nucleotide polymorphisms (SNP; ref. 12; Fluidigm, 96.96 Dynamic Array IFC). The data obtained were compared against a set of reference SNP profiles that have been authenticated by short tandem repeat (STR) back to a published reference (typically the supplying repository). Where a published reference STR profile was not available, the reference SNP profile is required to be unique within the collection/dataset. A minimum of 75% of SNPs is required to match the reference profile for a sample to be positively authenticated.

In addition, cell line underwent authentication via STR profiling at CellBank Australia in 2022. STR loci were amplified using the PowerPlex 16HS System (Promega) and the data were analyzed using GeneMapper ID software (ThermoFisher). The models were typically maintained for less than a month between thawing and being screened. The cell line stocks were authenticated using SNP and STR profiling. Details of cell lines are in Supplementary Table S1 and provided on the Cell Model Passport database (11).

### Compounds

Compounds were sourced from commercial vendors or supplied by pharmaceutical collaborators. The purity of all compound supplied by AstraZeneca compound management was >85% as determined by UV analysis of LC/MS chromatograms at 254 nm and substantiated using the total absorption chromatogram (TAC). DMSO solubilized compounds were stored at room temperature in a low humidity (<12%), low oxygen (<2.5%) environment. Details of compounds and drug combinations are in Supplementary Table S2. We included three compounds in our screen outside the AstraZeneca portfolio that have not yet completed clinical trials that are available for purchase from vendors: SCH7729 (48), prexasertib (49), and SG3199 (50).

### Screening

Cells were transferred into 1,536 microwell plates within 7.5 μL of the appropriate media. The seeding density of each cell line was optimized to ensure they remained in the growth phase throughout the duration of the assay. Assay plates were then incubated at 37°C in a humidified atmosphere at 5% CO_2_ for 24 hours prior to dosing with the compounds. Final DMSO concentrations were typically 0.2% and the duration of drug treatment was 72 hours. Cell viability was measured using CellTiter Glo 2.0 (Promega), 2.5 μL was added to each well, plates incubated for 10 minutes and quantification performed using a luminescence microplate reader.

### Controls

Each assay plate contains widely distributed controls wells including, two sets of negative controls *n* = 155 (wells receiving either no treatment or those treated with DMSO only), positive controls *n* = 32 (wells treated with either MG-132 or Staurosporine), and blank wells *n* = 28 (media only, no cells). To ensure high quality data, we used quality control metrics of the screen: 1,536 microtiter screening plates passing coefficient of variation (CV; threshold: CV ≤ 0.17985, median: 0.1228, range: 0.1252; Supplementary Fig. S1A) and Z-factor (threshold: Z-factor ≥ 0.3, median: 0.498 and range: 0.54945 for both positive controls; Supplementary Fig. S1B) thresholds.

### Quality Control

Strict quality controls were applied to each assay plate and across the screen. An assay plate is required to have a negative control coefficient of variation (CV) below 0.18 which is calculated using the DMSO-treated wells (NC-1).




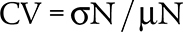




With σN the SD of the negative controls and μN the mean of the negative controls.

The effect of DMSO on cell viability is also assessed using the untreated and DMSO-treated negative control wells. The DMSO concentration in the negative control wells is equivalent to that of the combination treatment wells (0.2%). Plates are required to have an NC-0/NC-1 ratio of between 0.8 and 1.2 calculated using the mean of each negative control. Z-factors are calculated using the negative control (NC-1) and each positive control (PC1, PC2, & B). Where cell lines are sensitive to a positive control (NC-1/PC ratio ≥ 4), the Z-factor is required to be above 0.3 (a small proportion of lines ∼5% have a lower threshold of 0.2).




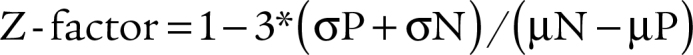




With σN and σP, the SD of the negative and positive controls, and μN and μP the mean of the negative and positive controls, respectively. Across all plates in a screen the mean and median Z-factors will be >0.4.

A subset of seven cell lines (A375, HT-29, PC-14, U-2-OS, SW620, C32, and MHH-ES-1) are screened in technical triplicate on six occasions. This generates 18 replicates for every compound across each of the seven lines, provided all plates meet quality controls and enables reproducibility to be investigated. Correlations between single-agent and combination and synergy metrics for replicated cell lines are shown in Supplementary Fig. S1C.

In addition, we compare the response of each drug across all the technical and biological replicates for the seven replicate cell lines to identify any systematic error or inconsistency. Drugs were flagged as failing QC when they demonstrate the following: either significant inconsistency across two or more dose points, or the behavior is observed in two or more of the replicate lines. Compounds meeting these criteria were failed and removed from the screen.

### Curve Fitting and Drug Responses

Fluorescent intensity measurements of drug-treated wells (CellTiter Glo assay) were normalized to a cell growth inhibition scale between a maximum of 1 (mean of blank wells) and a minimum of 0 (mean of DMSO control wells). Dose responses on this scale for individual library drugs are fitted to a two-parameter logistic curve using a nonlinear mixed effects model (51). The fitted response at the highest screened dose is reported as the single-agent Emax. Combination treatments are normalized but not fitted. As a precaution against outlying results, the combo Emax is the second highest reported inhibition value for a given 7 × 7 matrix. Results are in Supplementary Table S18.

### Synergy Measurements

Synergy of combinations is measured using two metrics, Bliss excess (15) and HSA (16, 52). For Bliss excess, the single-agent activities of drug A and drug B must be expressed as a probability between 0 and 1 (0 ≤ *E_A_* ≤ 1 and 0 ≤ *E_B_* ≤ 1). The observed effect of the combination is also expressed as a probability: (0 ≤ *E_AB_* ≤ 1). This means that the expected Bliss additive effect can be expressed as *E_A_* + *E_B_*(1 − *E_A_*) = *E_A_* + *E_B_* − *E_A_E_B_*. A positive “excess” over the expected Bliss additive effect defines a synergistic response. For HSA, a combination of drug A and drug B is classified as synergistic if the effect of the combination is larger than the effect of either drug A alone or drug B alone, whichever is larger: a positive HSA value therefore indicates synergy. Both metrics are reported as either the highest Bliss/HSA value found across the entire 7 × 7 dose matrix (“Bliss matrix”, “HSA matrix”), or as the highest value measured across the 25 possible 3 × 3 submatrices, or “windows”, across the 7 × 7 dose matrix (“Bliss window,” “HSA window). Synergy metrics are calculated in this way to provide global and local views of synergy to enable identification of local, dose-specific maxima of synergy that may be “canceled out” when considering the full dose matrix. During the course of screening, it was decided that the top two highest concentrations for the wee1 inhibitor AZD1775 were too high to give biologically relevant results, and so for these combinations the 7 × 7 matrix was cut down to 5 × 5, removing the two highest doses of both drugs for AZD1775-containing combinations only. Results are in Supplementary Table S18.

### Biomarkers

GDSCTools (20) was used to perform ANOVA biomarker discovery using single-agent and combo Emax, and Bliss matrix as inputs. Significance cutoffs of *P* ≤ 0.001 and FDR ≤ 10% and both Glass deltas ≥1 were applied to filter results. Biomarkers were identified in either pan-cancer, within common cancer types, or within specific genomic “basket” (common genotypes: TP53, KRAS, PIK3CA, MLL2, PTEN, BRAF) settings by subsetting the cell lines used for each ANOVA using information on cancer type from Cell Model Passports (11, 13), or information on mutational status in the multi-omics binary event matrix (13). 5,498,585 tests were performed, of which 11,611 passed significance thresholds. Results are in Supplementary Table S13.

### Biomarker Features

A multi-omics binary event matrix (“MOBEM”) of mutational, gene fusion, CNA, and methylation features (number of features = 1,073) previously found to be informative for predicting single-agent drug response in cell lines (13) were used as a feature dataset for biomarker discovery (the “Sanger MOBEMs”). This was supplemented with additional binary genomic and molecular biology features (number of features = 586, the “AZ MOBEMs”) curated from public datasets. To identify CRISPR gene dependencies that are significantly associated with clinically relevant molecular alterations, we implemented a framework that integrates TCGA (The Cancer Genome Atlas; RRID:SCR_003193) data with DepMap (Cancer Dependency Map Portal; RRID:SCR_017655) annotations. These alterations are either recurrently mutated genes (indicated by the gene name) or recurrent chromosomal regions that are lost or gained (indicated as gain:cna or loss:cna and a gene name where a known cancer gene is present in that chromosomal segment). All clinically recurrent mutations and CNAs identified in each tumor type are mapped on to >1,000 cancer cell lines. We defined whether a gene was an oncogene or a tumor suppressor gene using the OncoKB database (53) .Copy-number regions in cell lines were defined as “gain” or “loss” if log_2_(Segment_Mean) >1 or ≤1, respectively, for that region. We defined for all common TCGA tumor types (The Cancer Genome Atlas; RRID:SCR_003193):

(i) Driver-mutated cancer genes (CGs) specific for each tumor type [refs. 54, 55; TCGA (RRID:SCR_003193)]
(ii) Recurrent copy-number regions amplified or deleted per tumor type (56)
(iii) ER expression status (breast cancer) and ERBB2 (HER2) expression
(iv) Microsatellite instability (MSI; ref. 57)

For ER/ERBB2 status, we used expression for ER and ERBB2 as defined by the CCLE team at the Broad Institute. For cell lines with RNA-sequencing (RNA-seq) data, they used a probabilistic model to classify the status. The classification was consistent in both RNA-seq and reverse-phase protein analysis, and with the previous knowledge. This classification was equivalent to log_2_(RPKM+1) > 1.5. For cell lines for which they did not have RNA-seq data, the status from published data was used.

In addition, previously published RNA-seq gene expression data (21) was filtered to a panel of 672 genes representing known targets of the drugs used in the screen and their family members, genes encoding receptor tyrosine kinases, genes associated with the DNA damage superpathway (58), plus genes known to be clinically relevant in the oncology clinic (22) and genes annotated as mutated in the MOBEM. The gene expression dataset was then binarized across the relevant cell line panel subsets by a Z score ≥2 equating “GeneX_up” and a Z score ≤−2 equating to GeneX_down (number of features = 1,344). In addition, binarized PAM50 status (number of features = 9; refs. 23, 24) was also used as a biomarker feature for breast carcinoma cell lines.

### Protein Interaction and Synthetic Lethality Assessment

Protein interaction maps were generated in STRINGdb (59) with the following “source” filters applied–Experiments, Databases, Gene Fusions. Each edge captures confidence in the interaction with the minimum threshold of 0.4. Synthetic lethality assessment of all broadly active combination targets was performed using SynLethDB 2.0 (60).

### Enrichment Assessment of Synergistic Pathways Over Random

All synergistic combinations with targets and pathways (Supplementary Table S18) and implemented the threshold 0.1 HSA, 0.5 Emax to define efficacious combinations (“n”). Next, we calculated the total number of combinations per pathway using the full data matrix represented in Fig. 1C (“Nc”). To assess randomness within the combination–pathway relationship, we generated random numbers (from 1-n) and assigned them to each pathway combination. This was performed by bootstrapping 10-fold with an upper limit of “n” and calculating average for each pathway combo category (“nb”). The number of pathway combinations for only synergistic combinations per category (“nc”). Ratio of pathway combinations with synergistic combinations versus total number of combinations. “Es = nc/Nc” for each pathway category - Es (enrichment for synergy). Pathway combinations with weight from bootstrap “Er = nb/Nc” for each pathway category - Er (enrichment by random). Enriched for synergy over random if Es > Er. Code for this analysis is included in the publication's Github repository.

### Additional Cell Culture

WSU-DLCL2 cells were maintained in RPMI1640 (Gibco) supplemented with 10% (v:v) heat-inactivated FBS (Sigma Aldrich, catalog no. F4135), 2 mmol/L l-Glutamine, and 50 U/mL penicillin–streptomycin.

NOMO1 cells were obtained from DSMZ and maintained in RPMI1640 (Gibco) supplemented with 10% FBS and 5% l-Glutamine.

AN3-CA cells were obtained from ATCC and maintained with DMEM supplemented with 1% FBS and 1% l-Glutamine. HEC1 cells were obtained from ATCC and maintained with McCoy 5a medium modified supplemented with 10% FBS. MFE-280 cells were obtained by European Collection of Animal Cell Cultures and MFE-296 cells were obtained from DSMZ. Both cells were maintained in minimum essential medium with 10% FBS and 1% l-Glutamine. All cells were incubated at 37°C under 5% CO_2_. All cell lines were authenticated and tested negative for *Mycoplasma* contamination.

### Drug Treatments and Cell Assays

For AZD2811 in combination with venetoclax studies, cells were seeded at 0.5E6 cells/mL in culture medium containing either 50 μmol/L Q-VD-OPH (Cayman Chemical; item no. 15260) pan-caspase inhibitor or vehicle 16 hours prior to dosing with compounds. Compounds were solubilized in DMSO at a stock concentration of 10 mmol/L and diluted in sterile PBS to a 10X solution. Falcon 96-well White Flat Bottom plates (Corning; catalog no. 353296) were seeded with 10X compounds and cells were added on top for a final assay volume of 100 μL/well. Staurosporine (5 μmol/L; Sigma Aldrich; catalog no. S5921) was used as a positive control for cell death. After 72 hours, 50 μL of CellTiter Glo (Promega; catalog no. G7572) was added on top of the cells, the plates were shaken for 2 minutes, and then left to incubate protected from light at room temperature for 30 minutes before reading luminescence on the Synergy Neo2 (BioTek) plate reader.

The Caspase-Glo-3/7 time course assay was conducted similarly to the 3-day growth assay, except that 100 μL/well of Caspase-Glo-3/7 (Promega; catalog no. G8090) was added onto the cells at the time point. Separate plates were used for each time point.

For the AKT inhibitor capivasertib in combination with the MCL1 inhibitor AZD5991 studies, cells were seeded overnight on white opaque plates (384w; Corning) at 2,500–5,000 cells per well in a 30 μL volume. Combinations were dosed using 5-point half-log dilutions at indicated doses using an Echo 555 acoustic liquid dispenser (Labcyte). Cell viability was measured at indicated time points after drug incubation using CellTiter-Glo (Promega). The percentage of viability was calculated by normalizing drug-induced luminescent measurements to a negative control (DMSO only). For pretreatment studies, DMSO or QVD was added to the media when cells were seeded; 16 hours later the combination was added and cell viability was measured at indicated time points.

For measurement of caspase activation, cells were incubated with compounds for 6 hours followed by addition of Caspase-Glo 3/7 (Promega) following the manufacturer-supplied protocol. The percentage of caspase activation was calculated by normalizing drug-induced luminescent measurements to maximum (100% mixture of 0.5 mmol/L AZD5991 and 0.5 mmol/L AZD4320 inhibitors) and minimum (DMSO only) controls.

For drug treatments used for light microscopy or harvesting protein, cells were seeded overnight in 6-well plates (Corning) at 30%–80% confluency. Compounds were manually added at indicated doses and harvested at indicated time points.

For the cell viability 6 × 6 drug combination matrix studies, 1,000–2,000 cells were seeded in 384-well black plates (Greiner Bio-One Ltd, #781090) and incubated overnight at 37°C, 5% CO_2_. Cells were dosed using an Echo 555 acoustic liquid dispenser (Labcyte). Cell viability was measured at the time of dosing (day 0) and 72 hours after drug incubation using CellTiter-Glo (Promega) according to the manufacturer's instructions. Cell viability values were normalized to day 0 and the day 3 DMSO and were analyzed using Genedata Screener to generate heat maps and calculate the HSA synergy score.

For siRNA experiments, AN3-CA (30 nmol/L siRNA) and MFE-296 (40 nmol/L siRNA) with 9,000 cells per 96 well) were reverse transfected using Lipofectamine RNAimax and siRNAs of a nontargeting pool (Dharmacon, D-001810–10–05) or a MCL1 pool (Dharmacon, L-004501–00–0005). After 18 hours, cells were treated with a drug dose range of 0.001 to 10 μmol/L. Seventy-four hours later, viability was measured with CTG and signal normalized to the DMSO control.

### Western Blot Analysis

Cells were collected and centrifuged before the pellet was lysed in a cold RIPPA buffer (Thermo Fisher Scientific, 89901) supplemented with HALT protease and phosphatase inhibitor cocktail (Thermo Fisher Scientific, catalog no. 78440). Samples were prepared with NuPAGE LDS Sample Buffer (4X; Thermo Fisher Scientific, catalog no. NP0007) and boiled at 95°C for 5 minutes. Protein samples were quantified using Pierce BCA Protein Assay Kit (Thermo Fisher Scientific, 23225) according to the manufacturer's instructions before equal amounts of proteins were loaded and separated on to 4%–12% NuPAGE or Bolt Bis-Tris protein gels, transferred to nitrocellulose membranes, and blocked with 5% (wt/vol) nonfat dry milk in TBST [20 mmol/L Tris-HCl (pH 7.6), 137 mmol/L NaCl, 0.1% Tween-20]. Membranes were probed with indicated primary antibodies overnight at 4°C. Horseradish peroxidase–conjugated secondary antibodies [Cell Signaling Technology (CST), 7074; 1:2,000] were diluted in 5% (wt/vol) nonfat dry milk in TBST and detected on autoradiographic films or using G:Box gel doc system (Syngene) or Amersham ImageQuant 800 imager (Cytiva) after incubating with the ECL or SuperSignal West Dura reagents (Pierce).

### Antibodies

The following antibodies were used in this study: pPRAS40(T246) catalog number CST 2997; pAKT(S473) CST9271; total AKT CST 4691; cleaved PARP CST 9542; cleaved caspase 3 CST 9664; vinculin Sigma V9131; GAPDH CST 2118; pERK CST 9101; BIM CST 2933; β-Tubulin CST 2146; MCL1 CST 5453.

### Xenograft Efficacy Studies

All experimental work involving the use of laboratory animals was conducted in accordance with the recommendations set forth in the Guide for the Care and Use of Laboratory Animals, 8th edition. Mice were housed under pathogen-free conditions in individual ventilated cages at the Association for the Assessment and Accreditation of Laboratory Animal Care accredited facilities at AstraZeneca or Champions Oncology (Rockville, MD). All studies were reviewed and approved by the respective Institutional Animal Care and Use Committees (IACUC); work at Champions Oncology was also reviewed for compliance with AstraZeneca's global ethics standards. All results were reported following the Animal Research: Reporting In Vivo experiments guidelines.

C.B.-17 scid mice were purchased from Charles River Laboratories for the WSU-DLCL2 study or Taconic for the NOMO-1 study. —Five- to 8-week-old mice were implanted with either 5 × 10^6^ luciferase-tagged WSU-DLCL2 tumor cells (WSU-DLCL2luc) or 2 × 10^6^ NOMO-1 cells with 50% Matrigel (Corning). Tumor volumes (measured by caliper), animal body weight, and tumor condition were recorded twice weekly for the duration of the study. The tumor volume was calculated using the formula: length (mm) × width (mm)^2^/0.52. Tumor growth inhibition from the start of treatment was assessed by comparison of the differences in tumor volume between control and treated groups.

Statistical significance was evaluated using a two-way ANOVA with Tukey test. Statistical significance is identified as follows: * 0.05 < *P* < 0.01, ** 0.01 < *P* < 0.001. For efficacy studies, mice were rando­mized on the basis of tumor volumes using stratified sampling, and enrolled into control and treatment groups.

### Software

Figures 1A and 3A were created using a licensed version of BioRender.com. Figures 4A and B, 5A, and 6A were generated using TIBCO Spotfire Analyze. Matrices in Figs. 6C, D, F and Supplementary Figs. S8A–S8C, S9A–S9C, S11A and S11B, S13A and S13B, S14A and S14B, and S15A were generated in Genedata Screener.

### Code Availability

Analysis code for the manuscript figures, biomarkers, and screening data fitting are available in the following repositories:

(i) https://github.com/eac54/Large-scale-pan-cancer-screening
(ii) https://github.com/CancerRxGene/gdscmatrixanalyser

### Data Availability

Screening data are available through Figshare (https://figshare.com/projects/Large-scale_pan-cancer_cell_line_screening_identifies_actionable_and_effective_drug_combinations/163378). Combination response data are available via the GDSC Combinations Website (https://gdsc-combinations.depmap.sanger.ac.uk/). Combination response data are visualized and explored at the screen, cancer type, combination, and cell line combination level. In addition, there are links to other widely used cancer pharmacogenomics resources such as CancerRXGene (61), Cell Model Passports (11, 61), Depmap (38), and Project SCORE (62), enabling researchers to fully explore their combinations and cancer type of interest in the context of other large public pharmacogenomic datasets.

## Supplementary Material

Supplementary Tables List of supplementary tables

Table S1 Supplementary Table 1 lists cell lines used in this study

Table S2 Supplementary Table 2 lists drugs and drug combinations used

Table S3 Supplementary Table 3 shows combination-cancer type pairs with 10 percent or more responder cell lines

Table S4 Supplementary Table 4 shows combination-cancer type pairs with less than 10 percent responder cell lines

Table S5 Supplementary Table 5 shows combinations with broad activity across tumor types

Table S6 Supplementary Table 6 shows combination-cancer type pairs with selective activity

Table S7 Supplementary Table 7 shows the top combination-cancer type pairs in hematological cancers showing activity in at least 10% of tested cell lines in that specific cancer type ranked based on their activity (% responders) and disease selectivity

Table S8 Supplementary Table 8 shows the top combination-cancer type pairs in solid tumors showing activity in at least 10% of tested cell lines in that specific cancer type ranked based on their activity (% responders) and disease selectivity

Table S9 Supplementary Table 9 shows the top 50 combinations (all cancer types) using different % response scoring thresholds

Table S10 Supplementary Table 10 shows the number of hits using different % response scoring thresholds

Table S11 Supplementary Table 11 lists combination drug categories

Table S12 Supplementary Table 12 shows the number of top combinations:cancer type pairs hits in each combination drug category

Table S13 Supplementary Table 13 shows significant biomarkers and significant emergent biomarkers

Table S14 Supplementary Table 14 shows the top 100 significant emergent biomarkers

Table S15 Supplementary Table 15 shows biomarkers associated with top 5 enriched pathways for each drug combination category used in this study (CD = cell death, CS = cell signaling)

Table S16 Supplementary Table 16 shows emergent biomarkers associated with top 5 enriched pathways for each drug combination category used in this study (CD = cell death, CS = cell signaling

Table S17 Supplementary Table 17 shows large effect size and significant Bliss and combo Emax biomarkers for selected top hits

Table S18 Supplementary Table 18 shows drug response data for single agents and combination treatments

Supplementary Figures S1-S16 Supplementary Figure 1: Additional overview of screen and quality control.
Supplementary Figure 2: Combination benefit in combination-cell line pairs.
Supplementary Figure 3: Biomarkers of single agent and combination activity.
Supplementary Figure 4: Combination activity of venetoclax plus AZD5991 in AML cell lines.
Supplementary Figure 5: Combination activity by cancer type for AZD5991 + AZ3202.
Supplementary Figure 6: Venetoclax + selumetinib and AZD5991 + selumetinib activity by cancer type.
Supplementary Figure 7: Venetoclax + selumetinib and AZD5991 + selumetinib activity by cancer type.
Supplementary Figure 8: Effect of MEK1/2 plus BCL2 inhibition on cell viability in AML cells.
Supplementary Figure 9: Effect of MEK1/2 plus MCL1 inhibition on cell viability in AML cells.
Supplementary Figure 10: Cancer type selectivity of venetoclax + AZD2811.
Supplementary Figure 11: Effect of Aurora kinase B or pan Aurora kinase plus BCL2 inhibition on cell viability in DLBCL cell lines.
Supplementary Figure 12: network analysis of AZD2811 plus venetoclax biomarkers and targets, and cancer type selectivity of AZD5991 + AZD5363.
Supplementary Figure 13: Further analysis of capivasertib (AZD5363) plus AZD5991 in endometrial cell lines.
Supplementary Figure 14: Effect of pan AKT inhibition plus MCL1 inhibition on cell viability in endometrial cell lines.
Supplementary Figure 15: Further analysis of capivasertib (AZD5363) plus AZD5991 and other inhibitors in endometrial cell lines.
Supplementary Figure 16: Effect of MCL1 genetic knockdown plus pan AKT inhibition on cell viability in endometrial cancer cell lines.

## References

[1] Devita VT Jr , Serpick AA, Carbone PP. Combination chemotherapy in the treatment of advanced Hodgkin's disease. Ann Intern Med 1970;73:881–95. 5525541 10.7326/0003-4819-73-6-881

[2] DeVita VT Jr . A selective history of the therapy of Hodgkin's disease. Br J Haematol 2003;122:718–27. 12930382 10.1046/j.1365-2141.2003.04541.x

[3] Boshuizen J , Peeper DS. Rational cancer treatment combinations: an urgent clinical need. Mol Cell 2020;78:1002–18. 32559422 10.1016/j.molcel.2020.05.031

[4] Morgan P , Brown DG, Lennard S, Anderton MJ, Barrett JC, Eriksson U, . Impact of a five-dimensional framework on R&D productivity at AstraZeneca. Nat Rev Drug Discov 2018;17:167–81. 29348681 10.1038/nrd.2017.244

[5] Cook D , Brown D, Alexander R, March R, Morgan P, Satterthwaite G, . Lessons learned from the fate of AstraZeneca's drug pipeline: a five-dimensional framework. Nat Rev Drug Discov 2014;13:419–31. 24833294 10.1038/nrd4309

[6] Menden MP , Wang D, Mason MJ, Szalai B, Bulusu KC, Guan Y, . Community assessment to advance computational prediction of cancer drug combinations in a pharmacogenomic screen. Nat Commun 2019;10:2674. 31209238 10.1038/s41467-019-09799-2 PMC6572829

[7] Jaaks P , Coker EA, Vis DJ, Edwards O, Carpenter EF, Leto SM, . Effective drug combinations in breast, colon and pancreatic cancer cells. Nature 2022;603:166–73. 35197630 10.1038/s41586-022-04437-2 PMC8891012

[8] Nair NU , Greninger P, Zhang X, Friedman AA, Amzallag A, Cortez E, . A landscape of response to drug combinations in non-small cell lung cancer. Nat Commun 2023;14:3830. 37380628 10.1038/s41467-023-39528-9 PMC10307832

[9] O'Neil J , Benita Y, Feldman I, Chenard M, Roberts B, Liu Y, . An unbiased oncology compound screen to identify novel combination strategies. Mol Cancer Ther 2016;15:1155–62. 26983881 10.1158/1535-7163.MCT-15-0843

[10] Close DA , Wang AX, Kochanek SJ, Shun T, Eiseman JL, Johnston PA. Implementation of the NCI-60 human tumor cell line panel to screen 2260 cancer drug combinations to generate >3 million data points used to populate a large matrix of anti-neoplastic agent combinations (ALMANAC) database. SLAS Discov 2019;24:242–63. 30500310 10.1177/2472555218812429

[11] van der Meer D , Barthorpe S, Yang W, Lightfoot H, Hall C, Gilbert J, . Cell model passports-a hub for clinical, genetic and functional datasets of preclinical cancer models. Nucleic Acids Res 2019;47:D923–9. 30260411 10.1093/nar/gky872 PMC6324059

[12] Garnett MJ , Edelman EJ, Heidorn SJ, Greenman CD, Dastur A, Lau KW, . Systematic identification of genomic markers of drug sensitivity in cancer cells. Nature 2012;483:570–5. 22460902 10.1038/nature11005 PMC3349233

[13] Iorio F , Knijnenburg TA, Vis DJ, Bignell GR, Menden MP, Schubert M, . A landscape of pharmacogenomic interactions in cancer. Cell. 2016;166:740–54. 27397505 10.1016/j.cell.2016.06.017 PMC4967469

[14] Yu C , Mannan AM, Yvone GM, Ross KN, Zhang Y-L, Marton MA, . Highthroughput identification of genotype-specific cancer vulnerabilities in mixtures of barcoded tumor cell lines. Nat Biotechnol 2016;34:419–23. 26928769 10.1038/nbt.3460 PMC5508574

[15] Bliss CI . The toxicity of poisons applied jointly 1. Ann Appl Biol 1939;26:585–615.

[16] Berenbaum MC . The expected effect of a combination of agents: the general solution. J Theor Biol 1985;114:413–31. 4021503 10.1016/s0022-5193(85)80176-4

[17] Tao Z-F , Hasvold L, Wang L, Wang X, Petros AM, Park CH, . Discovery of a potent and selective BCL-xl inhibitor with in vivo activity. ACS Med Chem Lett 2014;5:1088–93. 25313317 10.1021/ml5001867 PMC4190639

[18] Speranza G , Kinders RJ, Khin S, Weil MK, Do KT, Horneffer Y, . Pharmacodynamic biomarker-driven trial of MK-2206, an AKT inhibitor, with AZD6244 (selumetinib), a MEK inhibitor, in patients with advanced colorectal carcinoma (CRC). J Clin Oncol 30: 15s, 2012 ( suppl 15; abstr 3529).

[19] Zaman S , Wang R, Gandhi V. Targeting the apoptosis pathway in hematologic malignancies. Leuk Lymphoma 2014;55:1980–92. 24295132 10.3109/10428194.2013.855307 PMC4152229

[20] Cokelaer T , Chen E, Iorio F, Menden MP, Lightfoot H, Saez-Rodriguez J, . GDSCTools for mining pharmacogenomic interactions in cancer. Bioinformatics 2018;34:1226–8. 29186349 10.1093/bioinformatics/btx744 PMC6031019

[21] Garcia-Alonso L , Iorio F, Matchan A, Fonseca N, Jaaks P, Peat G, . Transcription factor activities enhance markers of drug sensitivity in cancer. Cancer Res 2018;78:769–80. 29229604 10.1158/0008-5472.CAN-17-1679 PMC6522379

[22] Griffith M , Spies NC, Krysiak K, McMichael JF, Coffman AC, Danos AM, . CIViC is a community knowledgebase for expert crowdsourcing the clinical interpretation of variants in cancer. Nat Genet 2017;49:170–4. 28138153 10.1038/ng.3774 PMC5367263

[23] Parker JS , Mullins M, Cheang MCU, Leung S, Voduc D, Vickery T, . Supervised risk predictor of breast cancer based on intrinsic subtypes. J Clin Oncol 2009;27:1160–7. 19204204 10.1200/JCO.2008.18.1370 PMC2667820

[24] Ebbert MTW , Bastien RRL, Rowe LR, Miller PA, Anderson D, Boucher KM, . PAM50 breast cancer intrinsic classifier: clinical validation of a multianalyte laboratory developed test. J Clin Oncol 29: 15s, 2011 ( suppl 15; abstr 10597).

[25] Prahallad A , Sun C, Huang S, Di Nicolantonio F, Salazar R, Zecchin D, . Unresponsiveness of colon cancer to BRAF(V600E) inhibition through feedback activation of EGFR. Nature 2012;483:100–3. 22281684 10.1038/nature10868

[26] Corcoran RB , Ebi H, Turke AB, Coffee EM, Nishino M, Cogdill AP, . EGFRmediated re-activation of MAPK signaling contributes to insensitivity of BRAF mutant colorectal cancers to RAF inhibition with vemurafenib. Cancer Discov 2012;2:227–35. 22448344 10.1158/2159-8290.CD-11-0341 PMC3308191

[27] Connolly K , Brungs D, Szeto E, Epstein RJ. Anticancer activity of combination targeted therapy using cetuximab plus vemurafenib for refractory BRAF (V600E)-mutant metastatic colorectal carcinoma. Curr Oncol 2014;21:e151–4. 24523613 10.3747/co.21.1661 PMC3921040

[28] Xie Z , Bailey A, Kuleshov MV, Clarke DJB, Evangelista JE, Jenkins SL, . Gene set knowledge discovery with Enrichr. Curr Protoc. 2021;1:e90. 33780170 10.1002/cpz1.90 PMC8152575

[29] Hubner SE , de Camargo Magalhães ES, Hoff FW, Brown BD, Qiu Y, Horton TM, . DNA damage response-related proteins are prognostic for outcome in both adult and pediatric acute myelogenous leukemia patients: samples from adults and from children enrolled in a Children's Oncology Group Study. Int J Mol Sci 2023;24:5898. 36982970 10.3390/ijms24065898 PMC10058043

[30] Adhikary U , Paulo JA, Godes M, Roychoudhury S, Prew MS, Ben-Nun Y, . Targeting MCL-1 triggers DNA damage and an anti-proliferative res­ponse independent from apoptosis induction. Cell Rep 2023;42:113176. 37773750 10.1016/j.celrep.2023.113176 PMC10787359

[31] Carter JL , Hege K, Yang J, Kalpage HA, Su Y, Edwards H, . Targeting multiple signaling pathways: the new approach to acute myeloid leukemia therapy. Signal Transduct Target Ther 2020;5:288. 33335095 10.1038/s41392-020-00361-x PMC7746731

[32] Jain N , Curran E, Iyengar NM, Diaz-Flores E, Kunnavakkam R, Popplewell L, . Phase II study of the oral MEK inhibitor selumetinib in advanced acute myelogenous leukemia: a University of Chicago phase II consortium trial. Clin Cancer Res 2014;20:490–8. 24178622 10.1158/1078-0432.CCR-13-1311 PMC4310865

[33] Lauchle JO , Kim D, Le DT, Akagi K, Crone M, Krisman K, . Response and resistance to MEK inhibition in leukaemias initiated by hyperactive Ras. Nature 2009;461:411–4. 19727076 10.1038/nature08279 PMC4119783

[34] McMahon CM , Ferng T, Canaani J, Wang ES, Morrissette JJD, Eastburn DJ, . Clonal selection with RAS pathway activation mediates secondary clinical resistance to selective FLT3 inhibition in acute myeloid leukemia. Cancer Discov 2019;9:1050–63. 31088841 10.1158/2159-8290.CD-18-1453 PMC11994087

[35] Shah OJ , Lin X, Li L, Huang X, Li J, Anderson MG, . Bcl-XL represents a 34 druggable molecular vulnerability during aurora B inhibitor-mediated polyploidization. Proc Natl Acad Sci U S A 2010;107:12634–9. 20616035 10.1073/pnas.0913615107 PMC2906553

[36] Brown FC , Urosevic J, Polanska U, Cosaert J, Pease JE, Pomilio G, . Targeting aurora kinase B with AZD2811 enhances venetoclax activity in TP53-mutant AML. Blood 2019;134:3930.

[37] Holbeck SL , Camalier R, Crowell JA, Govindharajulu JP, Hollingshead M, Anderson LW, . The National Cancer Institute ALMANAC: A comprehensive screening resource for the detection of anticancer drug pairs with enhanced therapeutic activity. Cancer Res 2017;77:3564–76. 28446463 10.1158/0008-5472.CAN-17-0489 PMC5499996

[38] Boehm JS , Garnett MJ, Adams DJ, Francies HE, Golub TR, Hahn WC, . Cancer research needs a better map. Nature 2021;589:514–6. 33500573 10.1038/d41586-021-00182-0

[39] Gonçalves E , Poulos RC, Cai Z, Barthorpe S, Manda SS, Lucas N, . Pan-cancer proteomic map of 949 human cell lines. Cancer Cell 2022;40:835–49. 35839778 10.1016/j.ccell.2022.06.010 PMC9387775

[40] Nusinow DP , Szpyt J, Ghandi M, Rose CM, McDonald ERIII, Kalocsay M, . Quantitative proteomics of the cancer cell line encyclopedia. Cell. 2020;180:387–402. 31978347 10.1016/j.cell.2019.12.023 PMC7339254

[41] Liu F , Zhao Q, Su Y, Lv J, Gai Y, Liu S, . Cotargeting of Bcl-2 and Mcl-1 shows promising antileukemic activity against AML cells including those with acquired cytarabine resistance. Exp Hematol 2022;105:39–49. 34767916 10.1016/j.exphem.2021.10.006

[42] Dunn S , Eberlein C, Yu J, Gris-Oliver A, Ong SH, Yelland U, . AKT-mTORC1 reactivation is the dominant resistance driver for PI3Kβ/AKT inhibitors in PTEN-null breast cancer and can be overcome by combining with Mcl-1 inhibitors. Oncogene 2022;41:5046–60. 36241868 10.1038/s41388-022-02482-9 PMC9652152

[43] Plana D , Palmer AC, Sorger PK. Independent drug action in combination therapy: implications for precision oncology. Cancer Discov 2022;12:606–24. 34983746 10.1158/2159-8290.CD-21-0212 PMC8904281

[44] Ianevski A , Giri AK, Gautam P, Kononov A, Potdar S, Saarela J, . Prediction of drug combination effects with a minimal set of experiments. Nat Mach Intell 2019;1:568–77. 32368721 10.1038/s42256-019-0122-4 PMC7198051

[45] Zagidullin B , Aldahdooh J, Zheng S, Wang W, Wang Y, Saad J, . DrugComb: an integrative cancer drug combination data portal. Nucleic Acids Res 2019;47:W43–51. 31066443 10.1093/nar/gkz337 PMC6602441

[46] Seo H , Tkachuk D, Ho C, Mammoliti A, Rezaie A, Madani Tonekaboni SA, . SYNERGxDB: an integrative pharmacogenomic portal to identify synergistic drug combinations for precision oncology. Nucleic Acids Res 2020;48:W494–501. 32442307 10.1093/nar/gkaa421 PMC7319572

[47] Liu Y , Hu B, Fu C, Chen X. DCDB: drug combination database. Bioinformatics 2010;26:587–8. 20031966 10.1093/bioinformatics/btp697

[48] Wong DJL , Robert L, Atefi MS, Lassen A, Avarappatt G, Cerniglia M, . Antitumor activity of the ERK inhibitor SCH772984 [corrected] against BRAF mutant, NRAS mutant and wild-type melanoma. Mol Cancer 2014;13:194. 25142146 10.1186/1476-4598-13-194 PMC4155088

[49] Angius G , Tomao S, Stati V, Vici P, Bianco V, Tomao F. Prexasertib, a checkpoint kinase inhibitor: from preclinical data to clinical development. Cancer Chemother Pharmacol 2020;85:9–20. 31512029 10.1007/s00280-019-03950-y

[50] Hartley JA , Flynn MJ, Bingham JP, Corbett S, Reinert H, Tiberghien A, . Preclinical pharmacology and mechanism of action of SG3199, the pyrrolobenzodiazepine (PBD) dimer warhead component of antibody-drug conjugate (ADC) payload tesirine. Sci Rep 2018;8:10479. 29992976 10.1038/s41598-018-28533-4 PMC6041317

[51] Vis DJ , Bombardelli L, Lightfoot H, Iorio F, Garnett MJ, Wessels LF. Multilevel models improve precision and speed of IC_50_ estimates. Pharmacogenomics 2016;17:691–700. 27180993 10.2217/pgs.16.15 PMC6455999

[52] Lehár J , Zimmermann GR, Krueger AS, Molnar RA, Ledell JT, Heilbut AM, . Chemical combination effects predict connectivity in biological systems. Mol Syst Biol 2007;3:80. 17332758 10.1038/msb4100116 PMC1828746

[53] Chakravarty D , Gao J, Phillips SM, Kundra R, Zhang H, Wang J, . OncoKB: A precision oncology knowledge base. JCO Precis Oncol 2017;2017:PO.17.00011. 28890946 10.1200/PO.17.00011 PMC5586540

[54] Martincorena I , Raine KM, Gerstung M, Dawson KJ, Haase K, Van Loo P, . Universal patterns of selection in cancer and somatic tissues. Cell 2017;171:1029–41. 29056346 10.1016/j.cell.2017.09.042 PMC5720395

[55] Bailey MH , Tokheim C, Porta-Pardo E, Sengupta S, Bertrand D, Weerasinghe A, . Comprehensive characterization of cancer driver genes and mutations. Cell 2018;173:371–85. 29625053 10.1016/j.cell.2018.02.060 PMC6029450

[56] van Dyk E , Reinders MJT, Wessels LFA. A scale-space method for detecting recurrent DNA copy number changes with analytical false discovery rate control. Nucleic Acids Res 2013;41:e100. 23476020 10.1093/nar/gkt155 PMC3643574

[57] Chan EM , Shibue T, McFarland JM, Gaeta B, Ghandi M, Dumont N, . WRN helicase is a synthetic lethal target in microsatellite unstable cancers. Nature 2019;568:551–6. 30971823 10.1038/s41586-019-1102-x PMC6580861

[58] Stelzer G , Rosen N, Plaschkes I, Zimmerman S, Twik M, Fishilevich S, . The GeneCards suite: from gene data mining to disease genome sequence analyses. Curr Protoc Bioinformatics 2016;54:1.30.1–1.30.33. 10.1002/cpbi.5 27322403

[59] Szklarczyk D , Kirsch R, Koutrouli M, Nastou K, Mehryary F, Hachilif R, . The 36 STRING database in 2023: protein-protein association networks and functional enrichment analyses for any sequenced genome of interest. Nucleic Acids Res 2023;51:D638–46. 36370105 10.1093/nar/gkac1000 PMC9825434

[60] Wang J , Wu M, Huang X, Wang L, Zhang S, Liu H, . SynLethDB 2.0: a webbased knowledge graph database on synthetic lethality for novel anticancer drug discovery. Database 2022;2022:baac030. 35562840 10.1093/database/baac030 PMC9216587

[61] Yang W , Soares J, Greninger P, Edelman EJ, Lightfoot H, Forbes S, . Genomics of Drug Sensitivity in Cancer (GDSC): a resource for therapeutic biomarker discovery in cancer cells. Nucleic Acids Res 2013;41:D955–61. 23180760 10.1093/nar/gks1111 PMC3531057

[62] Behan FM , Iorio F, Picco G, Gonçalves E, Beaver CM, Migliardi G, . Prioritization of cancer therapeutic targets using CRISPR-Cas9 screens. Nature 2019;568:511–6. 30971826 10.1038/s41586-019-1103-9

