# Large-scale pan-cancer cell line screening identifies actionable and effective drug combinations

**DOI:** 10.1158/2159-8290.CD-23-0388

**Published:** 2024-05-01

**Authors:** Azadeh C. Bashi, Elizabeth A. Coker, Krishna C. Bulusu, Patricia Jaaks, Claire Crafter, Howard Lightfoot, Marta Milo, Katrina McCarten, David F. Jenkins, Dieudonne van der Meer, James T. Lynch, Syd Barthorpe, Courtney L. Andersen, Simon T. Barry, Alexandra Beck, Justin Cidado, Jacob A. Gordon, Caitlin Hall, James Hall, Iman Mali, Tatiana Mironenko, Kevin Mongeon, James Morris, Laura Richardson, Paul D. Smith, Omid Tavana, Charlotte Tolley, Frances Thomas, Brandon S. Willis, Wanjuan Yang, Mark J. O’Connor, Ultan McDermott, Susan E. Critchlow, Lisa Drew, Stephen E. Fawell, Jerome T. Mettetal, Mathew J. Garnett

**Affiliations:** 1 Oncology R&D, AstraZeneca, Cambridge, UK; 2 Wellcome Sanger Institute, Cambridge, UK; 3 Oncology R&D, AstraZeneca, Waltham, MA, USA

## Abstract

Oncology drug combinations can improve therapeutic responses and increase treatment options for patients. The number of possible combinations is vast and responses can be context-specific. Systematic screens can identify clinically-relevant, actionable combinations in defined patient subtypes. We present data for 109 anti-cancer drug combinations from AstraZeneca’s oncology small molecule portfolio screened in 755 pan-cancer cell lines. Combinations were screened in a 7x7 concentration matrix, with over 4 million measurements of sensitivity, producing an exceptionally data-rich resource. We implement a new approach using combination Emax (viability effect) and Highest Single Agent (HSA) to assess combination benefit. We designed a clinical translatability workflow to identify combinations with clearly-defined patient populations, rationale for tolerability based on tumor type and combination specific ‘emergent’ biomarkers, and exposures relevant to clinical doses. We describe three actionable combinations in defined cancer types, confirmed *in vitro* and *in vivo*, with a focus on hematological cancers and apoptotic targets.

## Introduction

Many anti-cancer agents have limited single agent activity in the clinic, making drug combinations an important treatment strategy. The first successful combination chemotherapy, introduced more than 50 years ago, consisted of a cocktail of four drugs (cyclophosphamide, vincristine, procarbazine, and prednisone) and resulted in durable clinical responses in patients with Hodgkin’s lymphoma (1,2). These chemotherapy combinations were often determined empirically in the clinic using existing monotherapy treatments. The recent advent of molecularly-targeted agents has led to the development of more rationally-designed combinations. Inhibiting multiple nodes in either the same or parallel signaling pathways can help tackle problems such as pathway redundancy, feedback reactivation and tumor heterogeneity, all of which can contribute to reduced efficacy and disease progression (3). There are however several challenges that need to be addressed when identifying efficacious drug combinations. First, obtaining deep pharmacological profiles of available targeted and chemotherapeutic agents is a complex and resource-intensive task. Second, handling the scale of this data generation and analyses towards identifying ‘actionable’ combinations are difficult. Finally, clinically-translatable combinations that deliver patient benefits are rare.

Employment of several criteria into portfolio decisions has resulted in higher success rate during discovery and development of novel drugs (4,5), including considerations of the target (efficacy), safety, patient population, exposure, and commercial opportunity. Like single agent drugs, candidate drug combinations require demonstration of activity in a patient population of unmet clinical need, an efficacy profile similar or superior to existing treatments, confidence in the tolerability profile, and knowledge of the exposures required for activity. When possible, application of these principles to design and analysis of *in vitro* combination screens could increase the likelihood of gathering this crucial understanding early in the drug development process. Specifically, comprehensive analysis of multi-omics data for cell line panels can identify potential biomarkers that could lead to patient stratification in the clinic. Avoiding combinations which are broadly active across models in *in vitro* screens can exclude combinations which may also be active in cells without genetic alteration, which thus could have activity in healthy tissues, limiting tolerability. Finally, the design of dose-response surfaces covering a matrix of concentrations relevant to clinical exposures can help inform the right doses for development.

Several groups, including ourselves (6)(7,8), have published unbiased combination screens in cancer cell lines. These have generally focused on assessing large numbers of combination pairs in relatively small cell panels. For example, we published a pan-cancer study as part of the DREAM combination prediction challenge that included >11,500 experiments across 910 combinations in 85 cell lines (6); O’Neil et al. published >22,000 experiments across 583 combinations in 39 cell lines (9); and the NCI-ALMANAC study included >5,000 combinations in 60 cell lines of the NCI-60 panel (10). We also recently described screening subsets of 2,025 combinations across 125 cell lines for three cancer types (7). For all these studies, in addition to screening relatively few cell lines, drug combinations were tested using either a limited or a subset of a full concentration matrix, thereby limiting the ability to comprehensively examine the most relevant range. Here, to extend our knowledge of effective drug combinations beyond previous studies, we screened 109 drug combinations using a 7x7 concentration matrix across 755 cell lines and developed an end-to-end framework which led to the identification and validation of clinically-actionable drug combinations.

## Results

### Over 68,000 combination-cell line pairs screened in 41 cancer types

**Figure 1:**
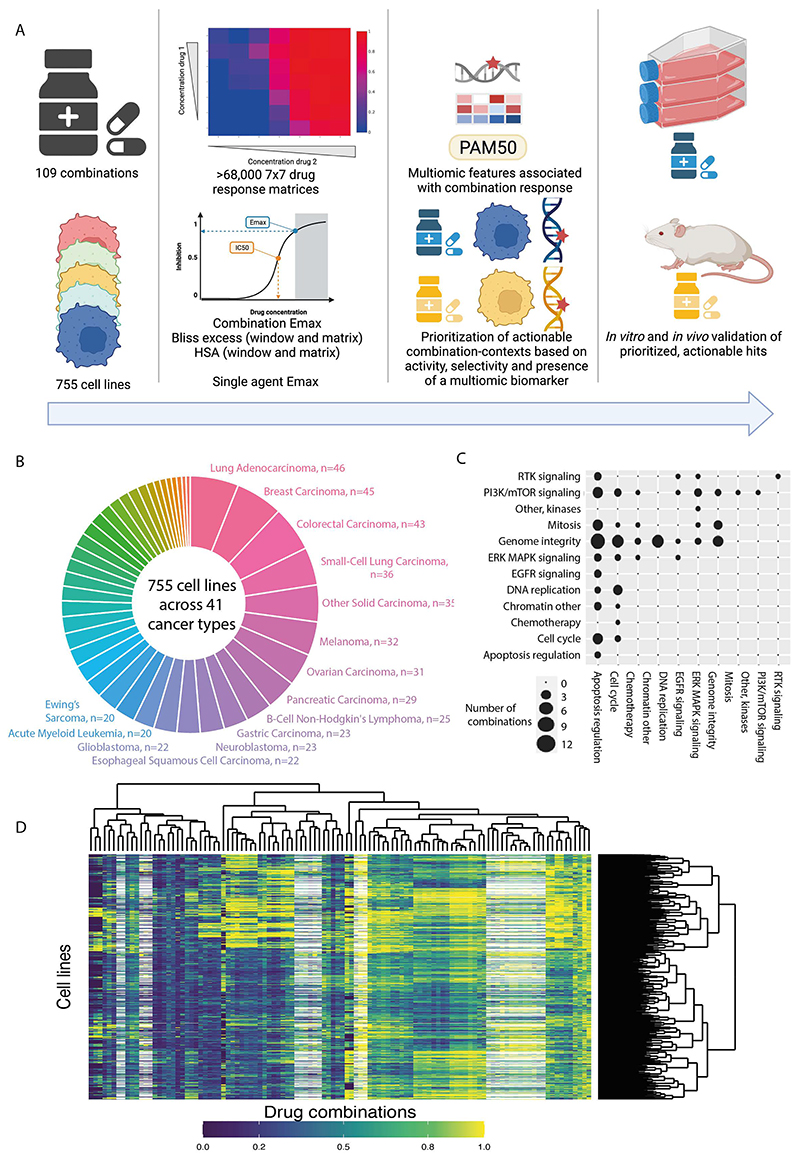
Dose response matrix combination screening landscape. A: Schematic of screen and analysis. Created with Biorender. B: Overview of cell line cancer types. C: Drug combinations screened grouped by drug target pathways. D: Combination Emax for 755 cell lines screened with 109 combinations. White represents combination/cell line pairs not screened.

We screened a diverse and molecularly-characterized panel of 755 cancer cell lines, covering 41 cancer types (11) (Figure 1A and B, Supplementary Table 1). Against these cell lines we screened 109 unique combinations of 37 individual drugs and investigational agents, with the majority coming from the AstraZeneca (AZ) portfolio covering diverse targets and mechanism of action with a particular focus on compounds targeting genome integrity, apoptosis and the cell cycle, which had potential for broad activity in a pan-tumor panel and which were of interest for clinical development (Figure 1C, Supplementary Table 2). Overall, this included 68,816 combination-cell line pairs: 82 combinations were screened in 755 cell lines and additional 27 combinations screened against a half cell line panel of 376 cell lines. To enable in-depth investigations of drug combination responses, we included a high coverage of chosen pathways, including combinations of drugs targeting apoptosis with genome integrity (Figure 1C).

Drugs were screened in a 7x7 combination matrix over a 1,000-fold concentration range of each drug chosen to cover IC_50_ values reported in previous single agent drug screens (12,13). The concentrations were informed by clinical relevance, including clinically achievable Cmax (maximum achievable concentration within the body). This design led to a wide range of combo Emax values (second highest viability effect) obtained across the screen (Figure 1D). As a control for screen quality, plates had low coefficient of variation, robust dynamic range as measured by Z-factor, and high correlation between control replicate screens (Figure S1A-C and see methods). Furthermore, there was a high correlation when comparing single agent IC_50_ values for six overlapping drugs screened using the same experimental platform (Figure S1D) (13), the GDSC2 (Genomics of Drug Sensitivity in Cancer 2) dataset (Pearson R=0.855; 735 common cell lines). Comparisons with the GDSC1 and PRISM monotherapy datasets had good (GDSC1 (12); Pearson R= 0.753; 699 common cell lines for 2 drugs) to moderate (PRISM (14); Pearson R= 0.513; 346 common cell lines for 13 drugs) correlations, with the lower correlations likely reflecting the use of different experimental platforms and protocols (Figure S1E-F). These results support the robustness of the screen.

We used multiple estimates of single agent and combination activity. These include two single agent IC_50_ values, the two single agent maximum viability reductions (single agent Emax; Figure S1G), combination maximum viability reduction (combo Emax, the second highest activity level seen in the matrix) (Figure S1H), and synergy scores, according to either the Bliss model (15) or Highest Single Agent (HSA) (16). HSA metric identifies drug combinations if the response is greater than either single agent alone. We report ‘matrix’ synergy scores averaged across all 49 wells of the combination matrix. In addition, we report ‘window’ synergy scores calculated across all 25 possible 3x3 sub-matrices of the 7x7 matrix and report the synergy score for the 3x3 ‘window’ with the largest synergy score (Figure S1I). The window synergy score is useful where synergy is concentration dependent. For example, IST-MES1 mesothelioma cell line treated with AZD5991 (MCL1 inhibitor) + AZ-3202 (BCL-Xli; also known as compound 15 (17)) had higher Bliss synergy excess for the window (0.823) versus matrix (0.180) (Figure S1J). Overall, 52.3% of combination-cell line pairs had a negative Bliss matrix excess and positive Bliss window excess, indicating that synergy was frequently observed within a narrow range of tested concentrations. Bliss excess was highly correlated with HSA excess (R = 0.924) (Figure S1K). In contrast, there was poor correlation between combo Emax with either HSA or Bliss synergy, consistent with some combination activity being driven by single agent activity (Fig S1L-M). Similarly, single agent Emax weakly correlates with combo Emax (Figure S1N), and single agent activity poorly correlates with synergy (Figure S1O).

Fitted and raw data are available through Figshare and the GDSC Combination website (https://gdsc-combinations.depmap.sanger.ac.uk/), where data can be visualized and explored at the screen, cancer type, combination and cell line-combination. Altogether, these data are a rich resource and show the value of acquiring multiple estimates of single drug and combination activity across a matrix of concentrations.

### Prioritization based on combination activity and tumor subtypes specificity

**Figure 2:**
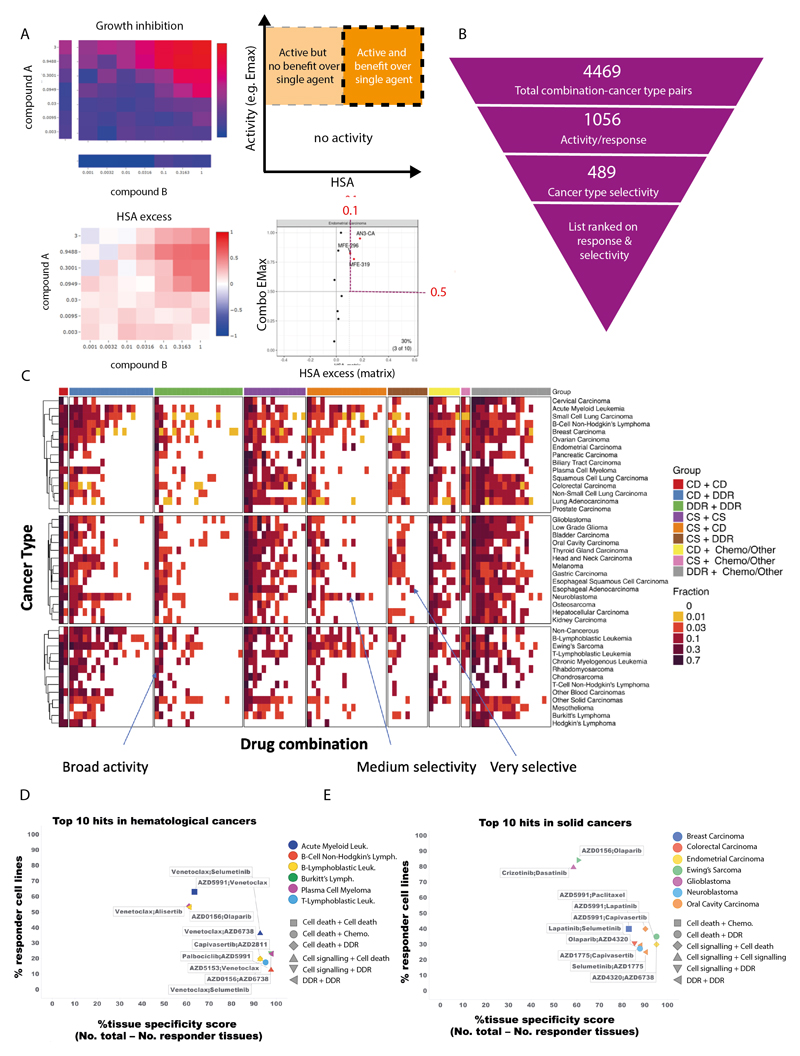
Shortlisting for active and selective combinations. A: Growth inhibition (Emax) and HSA matrix plots were generated for each combination in every cell line. Combination Emax and HSA were used to identify active combinations with benefit over single agent (s.a.). B: Combinations were filtered based on their activity and selectivity in the tested cancer types. C: Activity of each combination tested in this screen in 41 cancer types. The fraction of cell lines where the combinations are active is indicated and combinations are grouped by category. D and E: Top 10 hits in (D) hematological cancers and (E) solid tumors. Percentage of responder cell lines for each combination in each cancer type plotted versus cancer-type specificity scores. Each color represents a cancer type and combination categories are represented by different shapes. CD = cell death, DDR = DNA damage response, CS = cell signaling, chemo = chemotherapeutic agents.

From the over 68,000 combination-cell line pairs tested, we aimed to identify candidate combinations with the greatest potential to be taken forward into clinical development. Therefore, we sought to prioritize combinations with strong activity specifically focused within particular tumor subtypes. As a first step, we identified combination-cell line pairs with high activity (combination Emax>0.5) and combination benefit/synergy beyond single agent activity (HSA>0.1) (Figure 2A). We have previously screened nine combinations with a limited concentration matrix in up to 114 breast, colon and pancreatic cancer cell lines, representing 4,790 overlapping combination-cell line pairs (7). In support of our screening results, the classification of high activity and synergy beyond single agent activity agreed with the classification of synergy/non-synergy for 65.4% of combination-cell line pairs (Figure S2A). More examples of active combinations were identified in this study, supporting the value of our approach incorporating a 7x7 concentration matrix design and HSA metric.

We next identified combinations where at least 10% of cell lines tested within a specific cancer type fulfilled these criteria, reducing the number of combination:cancer type pairs by 76% from 4,469 to 1,056 (Figure 2B, Supplementary Table 3 and Supplementary Table 4). The minimum threshold of 10% was chosen to allow a relatively small number of models to highlight a potential combination, while maintaining a strong enough signal to support clinical actionability. Six combinations showed no combination benefit (HSA < 0.1, combination Emax <0.5) in the 755 cell lines tested, including the MCL1 inhibitor AZD5991 combined with either the DNAPK inhibitor AZD7648 or cMET inhibitor savolitinib; ATM inhibitor AZD1390 combined with either the MEK inhibitor selumetinib, the EGFR inhibitor gefitinib, or the AKT inhibitor capivasertib; and the PARP inhibitor olaparib combined with the BRD4 inhibitor AZD5153. Combination benefit was most enriched in combination:cell line pairs where the combination targeted ERK/MAPK and PI3K/MTOR signaling, or dual targeting of the cell cycle (Figure S2B).

The majority of the active combinations were active in multiple cancer types. Specifically, nineteen combinations were active in more than 50% of cancer types (Supplementary Table 5). Of these, five involved combinations targeting proteins that have a protein-protein/functional interaction from STRING database (prexasertib + AZD1775, SRA737 + AZD1775, AZD5991 + AZ3202, trametinib + taselisib, dasatinib + trametinib), and 3 combinations target synthetic lethal pairs of genes as defined by SynLethDB (AZD5153 + selumetinib, trametinib + taselisib, AZD5991 + AZ-3202)(Figure S2C). Broadly active combinations may be more likely to be active in normal tissues, thereby limiting their therapeutic window and potential for clinical development. For example, the combination of the MEK inhibitor, selumetinib, and the AKT inhibitor, capivasertib, had activity in 22 out of 41 cancer types. Despite strong preclinical activity here and in other studies, the overlapping clinical toxicities of selumetinib (inhibitor of MEK: MEKi) combined with MK2206 (AKTi) was found to prevent sufficient dose escalation to achieve the desired level of target inhibition, and clinical activity was not observed (18). However, more recent AKT inhibitors such as capivasertib or ipatasertib may have a broader therapeutic window on account of an ATP competitive mode of action, whereas MK2206 was an allosteric inhibitor. To maximize the therapeutic window of selected combinations, an additional filtering step was therefore applied to select combinations with high activity (HSA > 0.1 and Combination Emax > 0.5) in less than 50% of cancer types. This reduced the number of combination:cancer type pairs to 489 (Figure 2B-C and Supplementary Table 6).

As a final step of prioritization, drug combinations were ranked based on their activity (% responders in a cancer type) and cancer type selectivity (cancer type specificity score). The cancer type specificity score was calculated by subtracting the number of cancer types showing sensitivity to an individual drug combination (at least 10% responder cell lines) from the total number of cancer types. Activity and cancer-type selectivity were given equal weights and scores were given as a sum of percentage responders in that particular cancer type and the cancer type specificity score. Combination:cancer type pairs tested in less than 10 cell lines were excluded to prevent small sample sizes biasing the analysis, leading to a list of 99 combination:cancer type pairs in hematological cancers (Supplementary Table 7) and 252 combination:cancer type pairs in solid tumors. The top 100 combination:cancer type pairs are shown in Supplementary Table 8. This systematic approach informed our prioritized shortlist for prospective validation (see validation section below).

The top scoring combination in hematological cancers was selumetinib (MEKi) + venetoclax (BCL2i) in AML (response in 36% of AML cell lines), which also had above 10% activity in B-Lymphoblastic Leukemia (Figure 2D, Supplementary Table 6 and 7). Another highly ranked combination also included venetoclax, now with a cell death agent AZD5991 (MCL1i), with 63% responder cell lines in AML, but less selectivity across cancer types (active in 15 cancer types). For solid tumors, crizotinib + dasatinib in low grade glioma (87% responders; active in 16 cancer types) and AZD0156 (ATM/ATRi) plus olaparib (PARPi) in Ewing’s sarcoma (84% responders; active in 16 cancer types) were high scoring (Figure 2E, Supplementary Table 8). Several combination:cancer type pairs which have previously been shown to drive combination activity were identified, including AZD5991 (MCLi) + venetoclax (BCL2i) which was active in AML cell lines, and has been tested in a phase II trial in patients with AML (NCT03218683), providing support that our screen and prioritization process is capable of identifying clinically-relevant combinations in specific cancer types.

To evaluate the impact of our scoring thresholds on prioritized combinations, we investigated alternative thresholds for percentage response and cancer type specificity. Higher response thresholds (from more than 10% of cell lines to 25, 50 or 75%) led to a drop in the number of top hits (120, 14 and 3 respectively) by excluding combinations which are most cell line selective in their activity (Supplementary Table 9 and Supplementary Table 10). There was no change in the top 15 hits when the percentage response threshold was increased to 25% (Supplementary Table 9). Changing the cancer type specificity threshold (from less than 50% of cancer types to either less than 25% or 75%) also altered the number of combinations (223 and 727, respectively), either requiring combinations to be highly cancer type specific, or including widely active combinations which are less likely to be clinically tolerable.

With respect to the frequency of active combinations by cancer-type, AML had the highest number of active combinations within the top hematological cancer hits (25 combinations), followed by Chronic Myelogenous Leukemia (17 combinations) and B lymphoblastic leukemia (15 combinations) (Supplementary Table 7). In solid tumors, the highest number of active combinations within the top 100 hits were in Ewing’s sarcoma (16 combinations), followed by head and neck (9 combinations) and small cell lung carcinoma (8 combinations) (Supplementary Table 8).

To gain mechanistic insights into the top ranked combinations, we assigned combinations into nine categories based on the mechanism of action of the two constituent drugs (Supplementary Table 11). Out of the top 100 drug combination:cancer type pairs in solid tumors, 19 hits belonged to the ‘cell death’ plus ‘cell signaling’. However, in hematological cancers top hits, the highest number of combinations were the ones targeting ‘cell death’ plus ‘DNA damage response (DDR)’ pathways (31 hits). In contrast, combinations from the ‘cell signaling’ plus ‘chemotherapeutic agent’ category were overall rare (2 hits for hematological cancer and 2 hits for solid tumors) (Supplementary Table 7, Supplementary Table 8 and Supplementary Table 12). Out of the top ten drug combinations in hematological cancer, 7 included at least one compound targeting the ‘cell death’ pathway (Figure 2D). This finding is in agreement with the fact that apoptosis/cell death pathways are frequently dysregulated in hematological cancers leading to efficacy of cell death agents in these tumors (19). Overall, our prioritization approach enriched for combinations which are selectively active in subsets of cell lines and by tumor type, increasing the probability of identifying combinations with a clinically manageable tolerability profile.

### Multi-omics analysis identifies biomarkers of combination response

**Figure 3:**
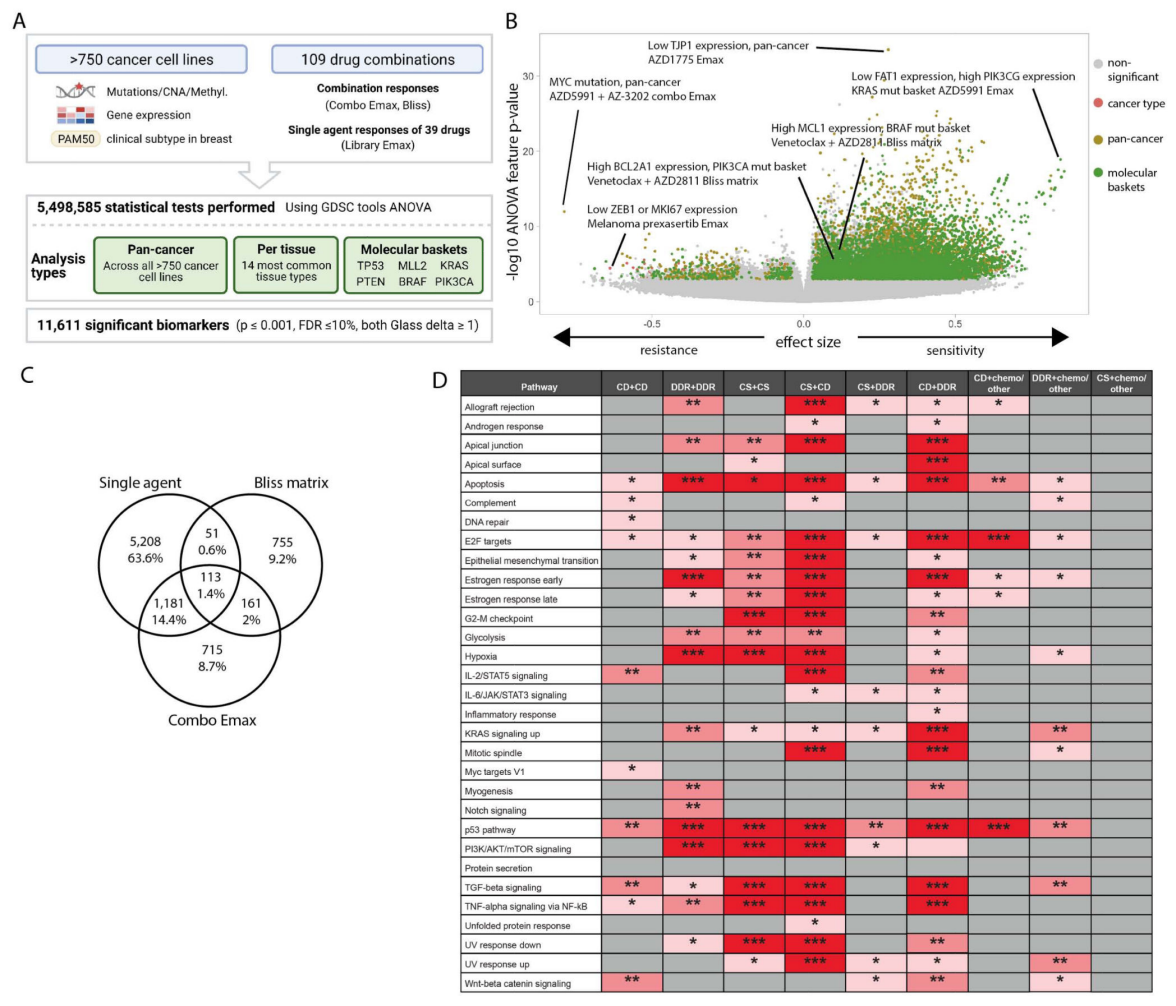
Multi-omics biomarkers of combination activity. A: Schematic of biomarker pipeline including molecular features incorporated and analyses performed. Created with BioRender.com. B: Volcano plot of biomarkers from all analyses. Statistically significant associations are coloured by analysis type, non-significant biomarkers are colo red gray. C: Venn diagrams of the biomarkers from different inputs leading to the identification of emergent biomarkers. Note that single agent biomarkers may be duplicated for the multiple combinations in which the single agent has been screened: the Venn diagram depicts unique single agent biomarker associations only. D: Significant enriched pathways for emergent biomarkers in each drug combination category based on adjusted p values. * 0.05 < p < 0.01, ** 0.01 < p < 0.001, *** p < 0.001 (CD = cell death, CS = cell signaling, chemo = chemotherapeutic agents).

We leveraged the large number of cell lines screened to understand how molecular context affects drug combination response. Using GDSCtools ANOVA (20), we performed 5.4 million statistical tests to identify statistically significant associations between drug response metrics and multi-omics features (Figure 3A, Supplementary Table 13). This included curated molecular features previously associated with single agent drug response (somatic mutations, copy number alterations and DNA methylation; n =1,073 (13)); additional molecular features curated from public datasets (see methods) (n = 586); a curated set of binarised gene expression features (n =1,344) (21,22); and PAM50 status for breast cancer cell lines (n=9) (23,24). Associations were identified with five response metrics, including single agent (compound 1 Emax, compound 2 Emax) and combination responses (combo Emax, Bliss matrix, Bliss window). Bliss was chosen over HSA as the synergy metric for biomarker identification because it is a more stringent measure of drug combination response and biomarkers of Bliss scores are more likely to identify drug interactions.

We identified significant associations for 21 different subgroups of cell lines. This included pan-cancer across the entire cell line panel; per cancer type for the 14 most common cancer types in our panel (>19 cell lines); and 6 molecular ‘baskets’ representing cancer type-agnostic cell line subpanels of the six most frequently mutated genes (*TP53* (n=477 cell lines), *KRAS* (n=107), *MLL2/KMT2D* (n=81), *PTEN* (n=72), *PIK3CA* (n=80) and *BRAF* (n=61)). Overall, we identified 11,611 statistically significant associations (p<=0.001, FDR<=10%, and positive and negative Glass delta >=1) (Figure 3A-B). This included 6,911 non-unique single agent and 4,700 combination biomarkers, representing at least one significant association for every combination tested (combo Emax associations, n =2,170; Bliss matrix, n =1,080; Bliss window, n =1,450). Cancer-type specific ANOVAs and molecular basket ANOVAs identified 803 and 4,388 context-specific biomarkers, respectively, in addition to those found in the pan-cancer setting, confirming the benefit of considering sensitivity biomarkers in specific molecular contexts (Figures S3A and B) (7).

A subset of biomarker associations were linked to the target of one or both of the drugs in a combination. For example, elevated expression of *PIK3CG* was associated with a greater Bliss window synergy score for AZD8186 (PI3Kβi) + palbociclib (CDK4/6i) in the KRAS molecular basket (Figure S3C). Elevated expression of *BIM* (BCL2L11) was significantly associated with higher AZD4320 (BCL2i, BCL-XLi) and venetoclax (BCL2i) single agent Emax values in the KRAS molecular basket (Figure S3D-E), and elevated *BCL2* expression was associated with higher venetoclax (BCL2i) Emax in the TP53 mutant basket (Figure S3F). In many instances, combination biomarkers were also associated with the single agent activity of a constituent drug. Specifically, 59.5% (6,911 of 11,611) of combination biomarkers were also biomarkers for at least one of the two monotherapies in that combination. This has been observed previously, for example *BRAF* mutation is a predictor of dabrafenib monotherapy activity in multiple cancer types and of response to dabrafenib-containing combinations in colon cancer (25–27).

We reasoned biomarkers specifically associated with combinatorial activity, and not with single agent activity of the individual constituent drugs, so called ‘emergent’ combination biomarkers, would be of particular interest because they are more likely to capture properties arising from drug-drug interactions. By excluding monotherapy-driven markers for each compound, we identified 14% of biomarkers (1,631 out of 11,611: 755 Bliss matrix only, 161 Bliss Matrix AND Combo Emax, 715 Combo Emax only; Figure 3C) as ‘emergent’ (Supplementary Table 13). Considering the top 100 most statistically significant emergent biomarkers (Supplementary Table 14), these involved ten unique combinations, seven of which included BCL2 or MCL-1 inhibitors which target cell death pathways (Supplementary Table 15, Supplementary Table 16 includes emergent biomarkers). To gain further insights into their properties, emergent biomarkers were grouped by signaling pathway prior to performing pathway enrichment analysis using EnrichR comparing single agent and emergent biomarkers for each mechanistic category of combination (28) (Figure 3D and Figure S3G). Apoptosis, P53 and E2F pathway were among the most enriched pathways across the combination categories. A smaller number of pathways were enriched in categories including chemotherapeutic agents, with no signaling pathway being enriched for the emergent biomarkers in the ‘cell signaling’ (non-DDR/cell death) plus chemotherapy category. Chemotherapeutics were broadly active in our screen, likely explaining why highly predictive markers of response were not observed. We hypothesize that these emergent biomarkers represent predictors of drug-drug interactions that cannot be readily identified from single agent activity alone, and thus highlight the potential utility of combination screens for biomarker identification over using monotherapy biomarkers alone.

### Combination Validation

We identified active and cancer-type selective combinations using our prioritization framework. A subset of the top scoring combinations are exemplified here and were validated *in vitro* and *in vivo* based on AZ portfolio interest, prior knowledge and to illustrate different types of therapeutic opportunities including new combinations and repurposing.

### AZD5991 plus venetoclax in AML

The second ranked combination in hematological cancers was venetoclax (BCL2i) + AZD5991 (MCLi) in acute myeloid leukemia (AML). For 13 of 19 AML cell lines the combination was active (HSA > 0.1 and combination Emax > 0.5) (Figure S4A-B). The combination was also active in other hematological cancers including Hodgkin’s lymphoma (42.9%, 3 of 7 cell lines), B-lymphoblastic leukemia (40%, 6 of 15 cell lines) and B Cell Non Hodgkin’s lymphoma (36%, 9 of 25 cell lines), as well for some solid tumors such as small cell lung carcinoma (47.2 %, 17 of 36 cell lines) and Ewing’s sarcoma (45%, 9 out of 20 cell lines) (Figure S4C). From our biomarker analysis, cell cycle and DNA repair pathways genes (e.g. *BRCA2, WEE1, CDC25A*) were associated with combination Emax, and downregulation of the nucleotide excision repair protein ERCC1 was associated with Bliss synergy, providing a putative mechanistic link between combination activity and DDR and cell cycle related pathways, as previously reported (29–31) (Figure S4D, Supplementary Table 17). Importantly, this combination was selective, with 40% (17/41) of tumor types having a response rate >10% and only 19% (8/41) of tumor types having a response rate >25%. In comparison, the combination of AZD5991 with another cell death target, the Bcl-xL inhibitor (AZ-3202), has poor selectivity with a >25% response rate in 90% (38/41) of tumor types (Figure S5A). The combination of AZD5991 + venetoclax was under clinical investigation in a phase 1/2a trial in patients with refractory or relapsed hematological malignancies but was recently terminated for undisclosed reasons (NCT03218683).

### Selumetinib plus venetoclax or AZD5991 in AML

**Figure 4:**
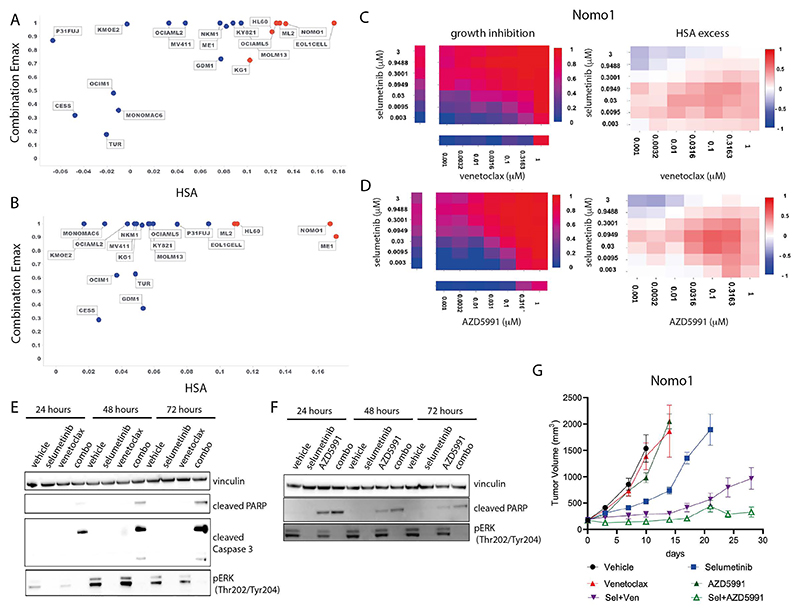
Combination activity of selumetinib plus venetoclax or AZD5991 in AML. A and B: Combination Emax versus HSA scores in 19 AML cell lines exposed to selumetinib combined with (a) venetoclax or (b) AZD5991. C and D: NOMO1 growth inhibition and HSA excess to the combination of selumetinib with (c) venetoclax or (d) AZD5991. E and F: Western blot for apoptosis markers in NOMO1 cells following time course treatment with selumetinib (300nM) combined with (e) venetoclax (300 nM) or (f) AZD5991 (100nM). G: Tumor growth in NOMO1 xenografts treated with selumetinib, AZD5991 or venetoclax alone or in combination for 28 days (n=5 each arm). Control and monotherapy experimental arms were halted once the maximum permitted tumor volume (2000cm^3^) was reached. Data are plotted as mean tumor volume +/- SEM.

Two further effective and specific combinations in AML were selumetinib (MEK1/2i) combined with venetoclax (BCL2i) or AZD5991 (MCLi) (Supplementary Table 7). Both combinations also had activity in other hematological cancers and solid tumors (Figure S6A-B). Of the nineteen AML cell lines included in the screen, six (EoL-1-cell, ML-2, OCI-AML5, NOMO-1, KG-1, HL-60) had strong combination activity when selumetinib was combined with venetoclax and four cell lines (ME-1, ML-2, NOMO-1, HL-60) when combined with AZD5991 (Figure 4A-D). These cell lines predominantly harbored alterations in MAPK pathway family members including OCI-AML5 (SOS1^N2337Y^, NF1^K1385R^), ML-2 (KRAS^A146T^), HL-60 (NRAS^Q61L^), and Nomo-1 (KRAS^G13D^). Consistent with these findings, among the significant biomarkers for venetoclax and selumetinib were proteins involved in ERK-MAPK signaling including EGF and SOS1, as well as members of the BCL2 family (PMAIP1) (Figure S7A-B, Supplementary Table 17).

To validate these two combinations *in vitro*, we assessed MAPK pathway inhibition and induction of apoptosis in the sensitive Nomo-1 cell line. Treatment with selumetinib completely inhibited phospho-ERK (pERK) levels tested for up to 72 hours. Neither venetoclax nor AZD5991 altered pERK levels (Figure 4E and 4F). When selumetinib was combined with venetoclax or AZD5991, pERK suppression was maintained and induction of cleaved PARP and cleaved caspase 3 was observed as early as 24 hours, increasing at 72 hours. AZD5991 alone caused a weak induction of cleaved PARP which was enhanced by the combination. Combination benefit was also achieved by combining selumetinib with an alternative selective MCL1 inhibitor, tapotoclax, or selective BCL2 inhibitor, S55746 (both currently under clinical investigation) in NOMO1, HL60 and ML2 cell lines. Similarly, an alternative MEK1/2 inhibitor trametinib was active in combination with the four MCL1 and BCL2 inhibitors tested (Figures S8A-C and S9A-C). Together, these results confirm on-target combination activity induces suppression of MAPK signaling and increased apoptosis.

We next evaluated the *in vivo* activity of the combinations using subcutaneous Nomo-1 xenograft models. Neither venetoclax (100 mg/kg oral daily) nor AZD5991 (two intravenous doses of 30mg/kg given two hours apart once weekly) alone caused any significant tumor growth inhibition when dosed as a monotherapy (Figure 4G). Whilst selumetinib monotherapy (10 mg/kg oral twice daily, 8 hours apart) led to 63% tumor growth inhibition (TGI) at day 10, tumors eventually grew out. Notably, combining selumetinib with venetoclax or AZD5991 markedly reduced tumor growth. Tumors treated with the selumetinib + venetoclax combination only reached a mean tumor volume of 963 mm^3^ after 28 days. The combination of selumetinib with AZD5991 was even more pronounced (88% TGI at day 10) and the mean tumor volume had not exceeded 400 mm^3^ by day 28. Collectively, these results confirm the *in vitro* and *in vivo* efficacy of these combinations in the setting of AML.

Venetoclax monotherapy in AML is only modestly active and significant benefit comes from addition of a second agent such as decitabine or cytarabine. Selumetinib has modest clinical activity as a monotherapy in AML patients (32). Given that the MAPK pathway is activated in about 70% of patients with AML due to mutations in upstream key proteins including RAS and FLT3 (33), and recent studies which show that further mutations in MAPK can arise from use of venetoclax or targeted therapies like gilteritinib (FLT3i), the use of a MEK inhibitor like selumetinib as a combination partner has strong rationale (34). Furthermore, the activity of selumetinib combined with AZD5991 also suggests an alternative partner in patients where BCL2 inhibition is insufficient to remove the anti-apoptotic blockade, and combination with an MCL1 inhibitor may be a good choice.

### AZD2811 plus venetoclax in DLBCL

An additional highly ranked combination was the aurora kinase B inhibitor (AurkB) AZD2811 + venetoclax in B-Cell Non-Hodgkin Lymphoma (NHL). The active pharmaceutical ingredient in AZD2811 (AZD1152) has previously undergone clinical evaluation for diffuse large B cell lymphoma (DLBCL), and the combination activity of aurora kinase B inhibitors and BH3 mimetics has been investigated in solid and hematological malignancies (35). AZD2811 + venetoclax has efficacy in *TP53* mutant and wildtype AML *in vitro* and *in vivo* models, and overcame venetoclax resistance in *TP53* models (36). However, despite these preclinical and clinical signals, the combination of Aurora kinase inhibitors with BCL2 inhibitors has not been reported to be active in DLBCL.

**Figure 5:**
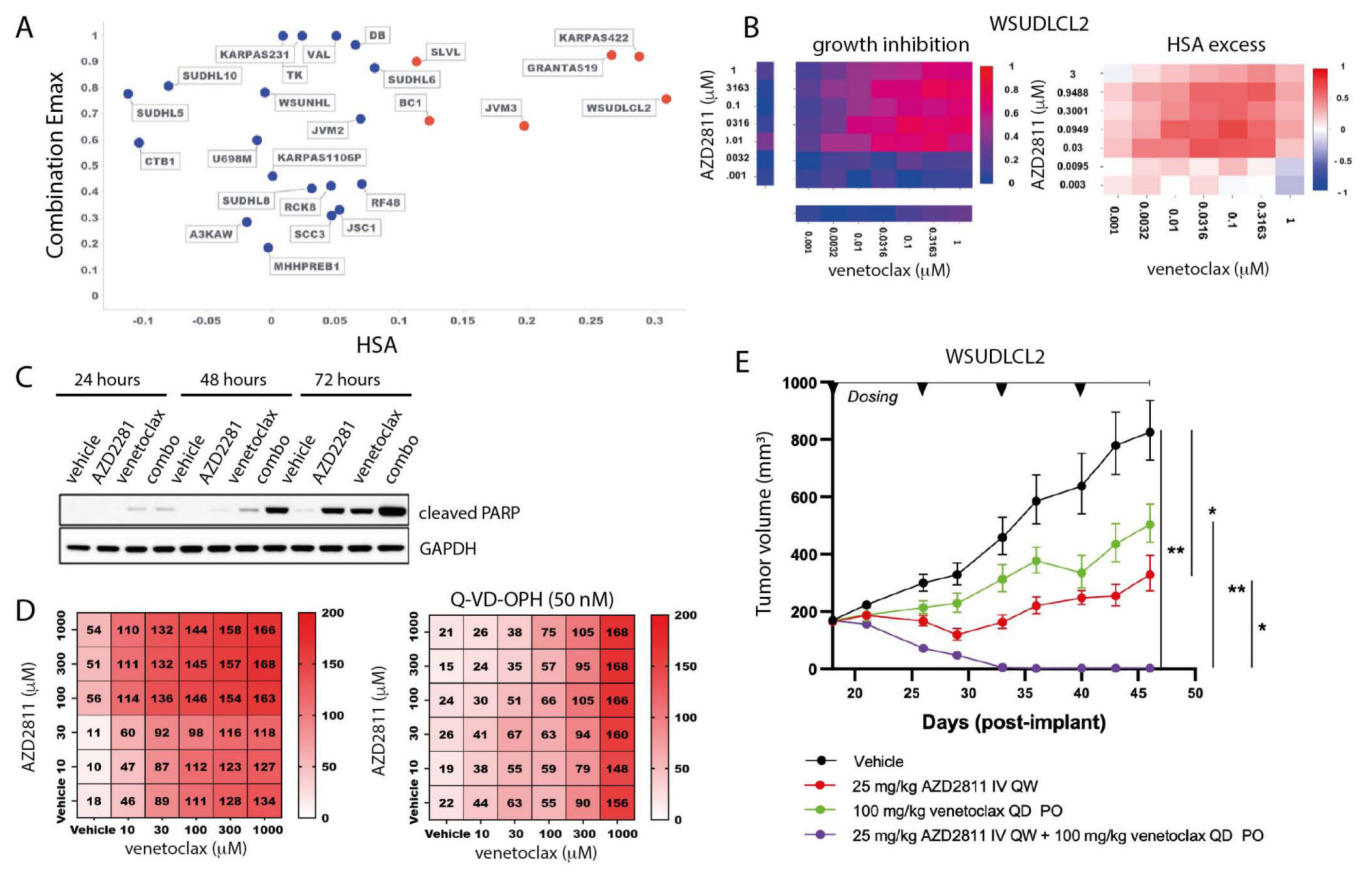
AZD2811 plus venetoclax combination in DLBCL. A: Combination Emax versus HSA in 25 B-cell NHL cell lines including 11 DLBCL cell lines. Cell lines with high combination activity (combination Emax > 0.5 and HSA > 0.1) are in red. B: Growth inhibition and HSA excess matrices in DLBCL cell line WSUDLCL2. C: Western blot for cleaved PARP in WSUDLCL2 cells treated with AZD2811 or venetoclax alone or in combination. D: Matrix plots indicating combination activity (measured by growth inhibition) in WSUDLCL2 cells pretreated with pan caspase inhibitor Q-VD-OPH and exposed to AZD2811 combined with venetoclax for 72 hours. Matrix values represent cell viability normalized to day 0 on the scale of 0 - 200 (value < 100 = percentage of growth inhibition, value > 100 = cell death). E: Tumor growth in WSUDLCL2 xenografts treated with AZD2811 or venetoclax alone or in combination for 46 days (n = 6 per group, * 0.05 < p < 0.01, ** 0.01 < p < 0.001). Data are plotted as mean tumor volume +/-SEM.

In our screen, 6 of the 25 B-Cell NHL cell lines had strong combination activity (HSA > 0.1 and combination Emax > 0.5), including 2 DLBCL cell lines (WSU-DLCL2 and KARPAS_422; Figure 5A and 5B). Combination activity was also seen in AML (5 of 19 cell lines), Ewing sarcoma (4 of 20 cell lines), plasma cell myeloma (3 of 13 cell lines) and small cell lung carcinoma (9 of 36 cell lines) (Figure S10A). In support of our screening results, combinations with alternative compounds targeting aurora kinase (danusertib) and BCL2 (S55748) had combination benefit in DLBCL models WSU-DLCL2 and KARPAS422 (Figure S11A-B). Except for upregulation of *CCNB1, MCL1* and *BCL2A1* gene expression in the *KRAS, BRAF* and *PIK3CA* baskets respectively, other significant biomarkers were non canonical to the cell cycle and cell death pathways which are the targets of the drugs (Figure S12A and Supplementary Table 17).

One of the responsive DLBCL cell lines, WSU-DLCL2, was selected for further *in vitro* and *in vivo* validation. The combination of venetoclax plus AZD2811 led to a time dependent induction of apoptosis compared to either single agent alone (Figure 5C), and combination activity was suppressed by pretreatment of cells with the pan-caspase inhibitor Q-VD-OPH (50nM; QVD) (Figure 5D). Additionally, *in vivo* anti-tumor activity of AZD2811 combined with venetoclax was assessed in mice bearing WSU-DLCL2luc xenografts. Once weekly intravenous administration of 25 mg/kg AZD2811 resulted in a statistically significant tumor growth inhibition (TGI) of 74%, while daily 100 mg/kg venetoclax resulted in 49% TGI but failed to reach statistical significance (Figure 5E). While both monotherapies were unable to prevent progressive tumor growth, the combination of AZD2811 and venetoclax drove striking activity, leading to tumor regression resulting in statistically significant complete regression (98% regression) by the third week of dosing. Together these studies support the *in vitro* and *in vivo* activity of venetoclax + AZD2811 in the setting of DLBCL.

### Capivasertib (AZD5363) plus AZD5991 in endometrial cancer

**Figure 6:**
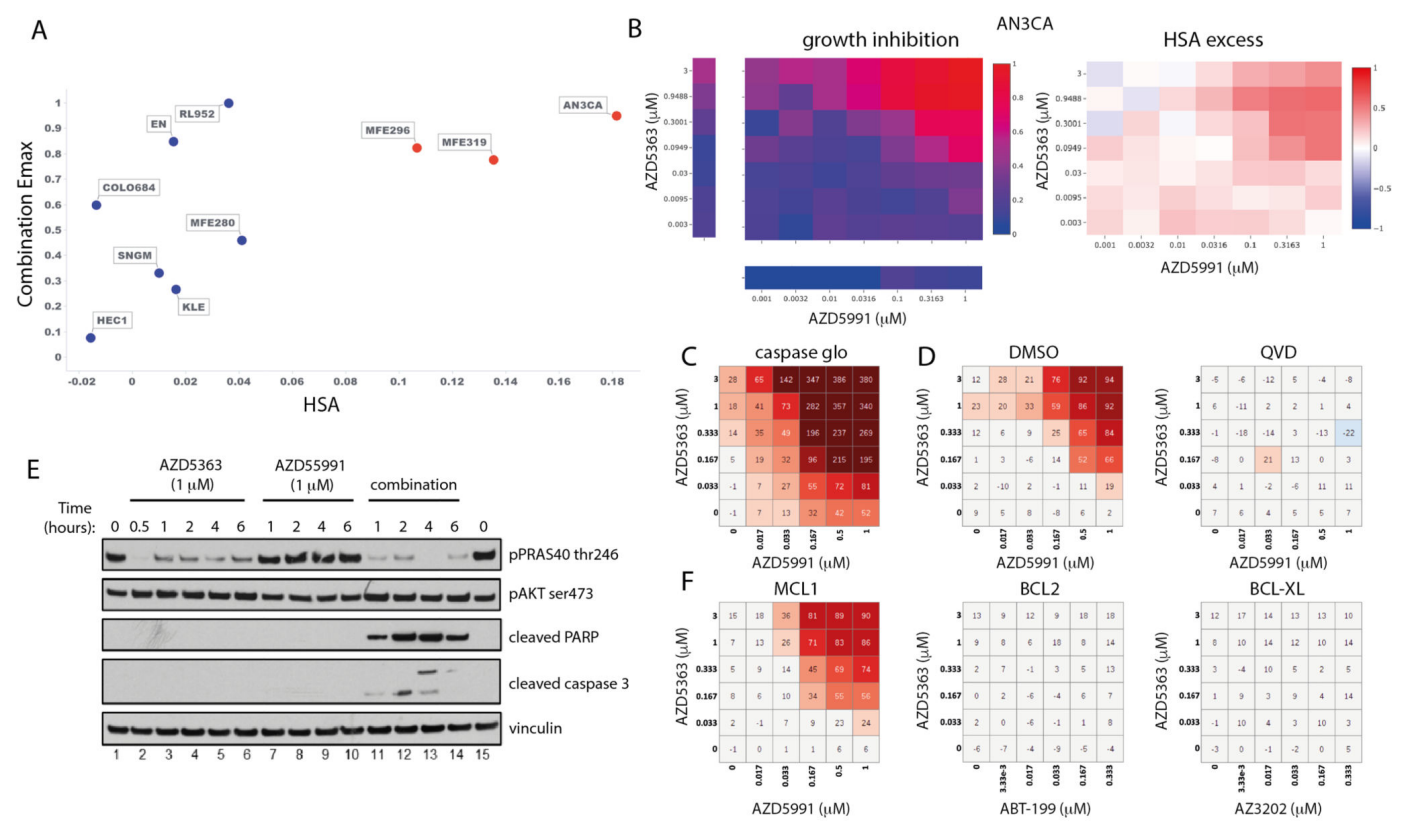
Capivasertib (AZD5363) plus AZD5991 combination activity in endometrial cell lines. A: Screening results of combination Emax versus HSA in endometrial cell lines treated with AZD5363 plus AZD5991. Cell lines with high combination activity are in red. B: Representative growth inhibition and HSA excess matrix plots in endometrial AN3CA cells. C: Matrix plot measuring apoptosis with AZD5991 and AZD5363 at indicated doses for 6 hours in AN3-CA cells. D: Matrix plots showing viability for AN3-CA cells pretreated with DMSO or QVD (caspase inhibitor) for 16 hours prior to the combination for 6 hours. E: Western blot analysis in AN3-CA cells treated with AZD5363 (1μm), AZD5991 (500 nM), or in combination at indicated times. F: Matrix plots showing viability in AN3-CA cells treated with AZD5991 or Venetoclax (ABT-199-BCL2 inhibitor), AZD4320 or AZ3202 (BCL-XL inhibitors) with AZD5363 at indicated doses for 6h.

The combination of the AKT inhibitor capivasertib (AZD5363) with the MCL1 inhibitor AZD5991 was one of the most selective combinations, active in only 2 of 41 cancer types (Figure S12B). The greatest responses were in endometrial cancer with 3 of the 10 endometrial cell lines showing strong combination activity (Figure 6A and 6B). Our biomarker analysis identified three significant associations involving DDR pathway genes (up-regulation of *BRCA2, RAD51* and down regulation of *ERCC1*), and upregulation of genes which directly or indirectly activate AKT (e.g *CDC25A* in the *TP53* basket and *RHOA* in the *PTEN* basket) were associated with combination Emax and Bliss score (Supplementary Table 17).

We chose two responder cell lines (AN3-CA and MFE-296) and two non-responder cell lines (HEC1 and MFE-280) for further validation. Both cell lines sensitive to the combination have *PTEN* mutations and had elevated baseline levels of phosphorylated AKT and PRAS40 (Figure S13A). Selective combination activity was confirmed in responsive and non-responsive lines, and notably became apparent as early as 3 hours, before either compound had single agent activity (Figure S13B - C). The combination of capivasertib and AZD5991 led to apoptosis (Figure 6C - E) as evidenced by induction of cleaved PARP and cleaved caspase 3 as early as 1 hour, as well as a marked induction of caspases (Figure 6C). Pre-treatment with the pan-caspase inhibitor QVD blunted apoptosis (Figure 6D).

To evaluate the on-target mechanism of action of the combination, we tested alternative compounds with similar target specificity. Combination activity was specific to MCL1 inhibition as both AZD5991 and tapotoclax (an alternative MCL1 inhibitor) showed combination benefit with AKT inhibition in responder cells (Figure S14A - B), whereas, the BCL2 inhibitor venetoclax (ABT-199) or a BCL-XL selective inhibitor AZ-3202 did not (Figure 6F). The combination effect with AZD5991 also occurred with the AKT inhibitors MK2206 and ipatasertib, as well as AZD8186 (PI3Kβ/δ), and to a lesser degree BYL719 (PI3Kα), but not the mTOR1 inhibitor rapamycin (Figure S14A - B and S15). Additionally, genetic knockdown of MCL1 in AN3-CA and MFE-296 cells caused a shift and reduction in the IC50 of AZD5363 and ipatasertib (Figure S16A-D). Taken together these results show marked combination activity through dual targeting the PI3K-AKT-pathway and MCL1 signaling axes in the setting of endometrial cancer.

## Discussion

In this study, 755 genomically-characterized cell lines from 41 cancer types were screened with 109 drug combinations using a 7x7 concentration matrix to generate over 4 million individual sensitivity measurements, of which more than 2.3 million describe combination response. Previous studies have been limited to a maximum of 125 cell lines (7) and consequently lack the same diversity of molecular backgrounds and cancer-types, which are known to impact treatment response. Furthermore, the use of full dose matrices uniquely provides an opportunity to identify effective combinations across a range of clinically-relevant concentrations with enhanced sensitivity compared to previous studies which have adopted a partial matrix approach (6–8,37). We anticipate that this dataset will be a rich resource and contribute to datasets available for these cancer cell lines as part of a Cancer Dependency Map (38).

Future analyses investigating combinations not prioritized in this study may yield additional actionable combinations, and the data availability will enable this. For example, combinations with activity limited to a small number of cell lines could have utility for a subset of patients if highly predictive markers could be identified. Similarly, combinations which were highly active across multiple tumor types, and so likely to be less tumor cell selective, may be tolerable for patients through the use of fractionated and alternative dosing schedules.

We capitalized on the availability of multi-omics data across all cell lines to not only identify biomarkers within a molecular basket (i.e. clinically-relevant genotypes), but also markers of monotherapy and combination response. We report emergent combination biomarkers that could not be readily explained by markers of response to the individual drugs. Such biomarkers, subject to validation, could provide insights into novel biology and signaling pathways driving combination efficacy. Future work incorporating newly available ‘omics’ data such as proteomics (39,40) may yield additional markers for combination opportunities and enable precision medicine approaches.

Our approach is designed to optimize preclinical interpretation with a focus on actionability. Rather than simply selecting combinations which elicited the greatest synergy, we shortlisted combinations that were highly active, more effective than monotherapy alone, and cancer-type selective. Several of the identified combination hits have already undergone clinical and preclinical development in the same cancer type as identified in our screen, such as the combination of the AZD5991 (MCL1i) + venetoclax (BCL2i) in AML (41) A factor driving our selection of combinations for experimental follow-up was having a rationale for at least one of the agents in the indication. For example, selumetinib (MEKi) + AZD5991 (MCL1i) in AML cell lines, which builds on reports showing combination activity in colorectal and melanoma cell models (30). The combination of AZD2811 (AurkBi) + venetoclax (BCL2i) was shown here to be active in DLBCL cell lines. This combination has activity in AML preclinical models (36). In addition, the combination of capivasertib (AKTi) + AZD5991 (MCL1i) has shown activity in breast cancer models (42) and was identified here as an active combination in endometrial lines. The PI3K/AKT/mTOR signaling pathway is frequently altered in endometrial cancer, therefore capivasertib + AZD5991 could represent an active and potent combination in this cancer type which currently lacks effective treatments. For multiple combinations we confirmed comparable activity using alternative inhibitors to the same targets, indicating that ‘on-target’ combination activity can be achieved with inhibitors in addition to the specific molecules tested here.

Future work should seek to refine and extend the current study. Focused screens in healthy or primary cells, albeit technically challenging, could control for potential combination toxicity. Similarly, tumor xenograft studies should be used to assess *in vivo* activity and tolerability. The inclusion of tumor stroma into screens could inform how the tumor microenvironment modulates combination response, and reveal new active combinations that target tumor cell - stroma signaling. A longer duration of combination exposure to cells might identify combinations that are dependent on cell division, and screening in 3D cultures could reveal combinations dependent on cell-cell interactions and 3D structure. Furthermore, many current clinically effective oncology drug combinations work through targeting tumor heterogeneity, a concept called independent action (43). The large heterogeneous panel of cell lines used here should enable analyses for independent drug action. Beyond the specific combinations identified, we anticipate that the experimental and analytical approach taken here will facilitate the interpretation of future drug combination studies. The richness of the full matrix design should enable data-driven approaches to better model combinatorial drug responses and to guide more efficient experimental designs based on optimized matrices, for example through subsampling the matrix or discontinuous dosing gradients (7,44). Furthermore, our study will be of interest to the fields of machine learning and computational biology, and as such complements previously published drug combination studies (6,9,37,45–47).

In conclusion, this study provides a rich resource and identifies actionable combinations as a starting point towards achieving the goal of developing rational combinations to improve treatment options for patients.

## Methods

### Cell Lines

The majority of cell lines were sourced commercially from repositories and cell banks. To facilitate high throughput screening all cell lines were maintained and screened in one of two media types; DMEM/F12 or RPMI supplemented with 10% FBS, Penicillin-Streptomycin and Sodium Pyruvate. All cell line stocks used for screening were tested for mycoplasma contamination prior to banking using both a polymerase chain reaction (EZ-PCR Mycoplasma Detection Kit, Biological Industries) and a biochemical test (MycoAlert, Lonza). Cultures testing positive using either method were removed from the collection.

To prevent cross-contamination or misidentification, all banked cryovials of cell lines were analyzed using a panel of 94 single nucleotide polymorphisms (SNPs) (12) (Fluidigm, 96.96 Dynamic Array IFC). The data obtained were compared against a set of reference SNP profiles that have been authenticated by short tandem repeat (STR) back to a published reference (typically the supplying repository). Where a published reference STR profile was not available, the reference SNP profile is required to be unique within the collection/dataset. A minimum of 75% of SNPs is required to match the reference profile for a sample to be positively authenticated.

In addition, cell line underwent authentication via STR profiling at CellBank Australia (Westmead, Australia) in 2022. STR loci were amplified using the PowerPlex® 16HS System (Promega) and the data were analyzed using GeneMapper™ ID software (ThermoFisher). The models were typically maintained for less than a month between thawing and being screened. The cell line stocks were authenticated using SNP and STR profiling. Details of cell lines are in Supplementary Table 1 and provided on the Cell Model Passport database (11).

### Compounds

Compounds were sourced from commercial vendors or supplied by pharmaceutical collaborators. The purity of all compound supplied by AstraZeneca compound management was >85% as determined by UV analysis of liquid chromatography-mass spectroscopy (LCMS) chromatograms at 254 nM and substantiated using the TAC (Total Absorption Chromatogram). DMSO solubilised compounds were stored at room temperature in a low humidity (<12%), low oxygen (<2.5%) environment. Details of compounds and drug combinations are in Supplementary Table 2. We included 3 compounds in our screen outside the AstraZeneca portfolio that have not yet completed clinical trials that are available for purchase from vendors: SCH7729 (48), prexasertib (49), SG3199 (50).

### Screening

Cells were transferred into 1536 microwell plates within 7.5μl of the appropriate media. The seeding density of each cell line was optimized to ensure they remained in the growth phase throughout the duration of the assay. Assay plates were then incubated at 37°C in a humidified atmosphere at 5% CO_2_ for 24 hours prior to dosing with the compounds. Final DMSO concentrations were typically 0.2% and the duration of drug treatment was 72 hours. Cell viability was measured using Cell Titer Glo 2.0 (Promega), 2.5μl was added to each well, plates incubated for 10 minutes and quantification performed using a luminescence microplate reader.

### Controls

Each assay plate contains widely distributed controls wells including, two sets of negative controls n=155 (wells receiving either no treatment or those treated with DMSO only), positive controls n=32 (wells treated with either MG-132 or Staurosporine) and blank wells n=28 (media only, no cells). To ensure high quality data we used quality control metrics of the screen: 1536 microtiter screening plates passing coefficient of variation (CV; threshold: CV≤0.17985, median: 0.1228, range: 0.1252 - Figure S1A) and Z-factor (threshold: Z-factor≥0.3, median: 0.498 and range: 0.54945 for both positive controls - Figure S1B) thresholds.

### Quality Control

Strict quality controls were applied to each assay plate and across the screen. An assay plate is required to have a negative control coefficient of variation (CV) below 0.18 which is calculated using the DMSO treated wells (NC-1).

CV=σN/μN

With σN the standard deviation of the negative controls and μN the mean of the negative controls.

The effect of DMSO on cell viability is also assessed using the untreated and DMSO treated negative control wells. The DMSO concentration in the negative control wells is equivalent to that of the combination treatment wells (0.2%). Plates are required to have an NC-0/NC-1 ratio of between 0.8-1.2 calculated using the mean of each negative control. Z-factors are calculated using the negative control (NC-1) and each positive control (PC1, PC2 & B). Where cell lines are sensitive to a positive control (NC-1/PC ratio ≥ 4) the Z-Factor is required to be above 0.3 (a small proportion of lines ~5% have a lower threshold of 0.2).

$$\;\text{Z}-\text{factor}=1-3^{*}(\sigma \text{P}+\sigma \text{N})/(\mu \text{N}-\mu \text{P})$$

With σN and σP the standard deviation of the negative and positive controls, and μN and μP the mean of the negative and positive controls, respectively. Across all plates in a screen the mean and median Z-Factors will be >0.4.

A subset of seven cell lines (A375, HT-29, PC-14, U-2-OS, SW620, C32 and MHH-ES-1) are screened in technical triplicate on six occasions. This generates 18 replicates for every compound across each of the seven lines, provided all plates meet quality controls and enables reproducibility to be investigated. Correlations between single agent and combination and synergy metrics for replicated cell lines are shown in Figure S1C.

Additionally, we compare the response of each drug across all the technical and biological replicates for the seven replicate cell lines to identify any systematic error or inconsistency. Drugs were flagged as failing QC when they demonstrate the following: either significant inconsistency across two or more dose points, or the behavior is observed in two or more of the replicate lines. Compounds meeting these criteria were failed and removed from the screen.

### Curve fitting and drug responses

Fluorescent intensity measurements of drug-treated wells (Cell Titer Glo assay) were normalized to a cell growth inhibition scale between a maximum of 1 (mean of blank wells) and a minimum of 0 (mean of DMSO control wells). Dose responses on this scale for individual library drugs are fitted to a two parameter logistic curve using a non-linear mixed effects model (51). The fitted response at the highest screened dose is reported as the single agent Emax. Combination treatments are normalized but not fitted. As a precaution against outlying results, the combo Emax is the second highest reported inhibition value for a given 7x7 matrix. Results are in Supplementary Table 18.

### Synergy measurements

Synergy of combinations is measured using two metrics, Bliss excess (15) and Highest Single agent (HSA) (16,52). For Bliss excess, the single agent activities of Drug A and Drug B must be expressed as a probability between 0 and 1 (0 ≤ *E*_*A*_ ≤ 1 and 0 ≤ *E*_*B*_ ≤ 1). The observed effect of the combination is also expressed as a probability: (0 ≤ *E*_*AB*_ ≤ 1). This means that the expected Bliss additive effect can be expressed as *E*_*A*_ + *E*_*B*_ (1 − *E*_*A*_) = *E*_*A*_ + *E*_*B*_ − *E*_*A*_*E*_*B*_. A positive ‘excess’ over the expected Bliss additive effect defines a synergistic response. For HSA, a combination of Drug A and Drug B is classified as synergistic if the effect of the combination is larger than the effect of either Drug A alone or Drug B alone, whichever is larger: a positive HSA value therefore indicates synergy. Both metrics are reported as either the highest Bliss/HSA value found across the entire 7 x 7 dose matrix (‘Bliss matrix’, ‘HSA matrix’), or as the highest value measured across the 25 possible 3 x 3 submatrices, or ‘windows’, across the 7 x 7 dose matrix (‘Bliss window’, ‘HSA window). Synergy metrics are calculated in this way to provide global and local views of synergy to enable identification of local, dose-specific maxima of synergy that may be ‘canceled out’ when considering the full dose matrix. During the course of screening it was decided that the top two highest concentrations for the wee1 inhibitor AZD1775 were too high to give biologically-relevant results, and so for these combinations the 7 x 7 matrix was cut down to 5 x 5, removing the two highest doses of both drugs for AZD1775-containing combinations only. Results are in Supplementary Table 18.

### Biomarkers

GDSCTools (20) was used to perform ANOVA biomarker discovery using single agent and combination Emax, and Bliss matrix as inputs. Significance cutoffs of p<=0.001 and FDR <=10% and both Glass deltas >=1 were applied to filter results. Biomarkers were identified in either pan-cancer, within common cancer types, or within specific genomic ‘basket’ (common genotypes: TP53, KRAS, PIK3CA, MLL2, PTEN, BRAF) settings by subsetting the cell lines used for each ANOVA using information on cancer type from Cell Model Passports (11,13), or information on mutational status in the multi-omics binary event matrix (13). 5,498,585 tests were performed, of which 11,611 passed significance thresholds. Results are in Supplementary Table 13.

### Biomarker features

A multi-omics binary event matrix (‘MOBEM’) of mutational, gene fusion, CNA and methylation features (number of features=1,073) previously found to be informative for predicting single agent drug response in cell lines (13) were used as a feature dataset for biomarker discovery - the ‘Sanger MOBEMs’. This was supplemented with additional binary genomic and molecular biology features (number of features = 586, the ‘AZ MOBEMs’) curated from public datasets. To identify CRISPR gene dependencies that are significantly associated with clinically relevant molecular alterations, we implemented a framework that integrates TCGA(The Cancer Genome Atlas (RRID:SCR_003193)) data with DepMap (Cancer Dependency Map Portal (RRID:SCR_017655)) annotations. These alterations are either recurrently mutated genes (indicated by the gene name) or recurrent chromosomal regions that are lost or gained (indicated as gain:cna or loss:cna and a gene name where a known cancer gene is present in that chromosomal segment). All clinically recurrent mutations and copy number alterations identified in each tumor type are mapped on to >1000 cancer cell lines. We defined whether a gene was an oncogene or a tumor suppressor gene using the OncoKB database (53). Copy number regions in cell lines were defined as ‘gain’ or ‘loss’ if log2(Segment_Mean) >1 or <-1 respectively for that region. We defined for all common TCGA tumor types (The Cancer Genome Atlas (RRID:SCR_003193)):

1. Driver-mutated Cancer Genes (CGs) specific for each tumor type (54,55)(The Cancer Genome Atlas (RRID:SCR_003193))
2. Recurrent copy number regions amplified or deleted per tumor type(56)
3. ER expression status (Breast cancer) and ERBB2 (HER2) expression
4. Microsatellite instability (MSI)(57)

ER/ERBB2 status - we used expression for ER and ERBB2 as defined by the CCLE team at the Broad Institute. For cell lines with RNA-seq data, they used a probabilistic model to classify the status. The classification was consistent in both RNA-seq and RPPA, and with the previous knowledge. This classification was equivalent to log2(RPKM+1) > 1.5. For cell lines for which they did not have RNA-seq data, the status from published data was used.

Additionally, previously published RNAseq gene expression data (21) was filtered to a panel of 672 genes representing known targets of the drugs used in the screen and their family members, genes encoding receptor tyrosine kinases, genes associated with the DNA damage superpathway (58), plus genes known to be clinically relevant in the oncology clinic (22) and genes annotated as mutated in the MOBEM. The gene expression dataset was then binarised across the relevant cell line panel subsets by a Z score >= 2 equating ‘GeneX_up’ and a Z score <=-2 equating to ‘GeneX_down (number of features=1,344). Additionally, binarised PAM50 status (number of features = 9) (23,24) was also used as a biomarker feature for breast carcinoma cell lines.

### Protein interaction and synthetic lethality assessment

Protein interaction maps were generated in STRINGdb (59) with the following ‘source’ filters applied – Experiments, Databases, Gene Fusions. Each edge captures confidence in the interaction with the minimum threshold of 0.4. Synthetic Lethality assessment of all broadly active combination targets was performed using SynLethDB 2.0 (60).

### Enrichment assessment of synergistic pathways over random

All synergistic combinations with targets and pathways (Supplementary Table 18) and implemented the threshold 0.1 HSA, 0.5 Emax to define efficacious combinations (‘**n**’). Next, we calculated the total number of combinations per pathway using the full data matrix represented in Fig. 1C ('**Nc**'). In order to assess randomness within the combination-pathway relationship, we generated random numbers (from 1-n) and assigned them to each pathway combination. This was performed by bootstrapping 10-fold with a upper limit of 'n' and calculating average for each pathway combo category (‘**nb**’). The number of pathway combinations for only synergistic combinations per category ('**nc**'). Ratio of pathway combinations with synergistic combinations vs total number of combinations. '**Es = nc/Nc**' for each pathway category - **Es (enrichment for synergy)**. Pathway combinations with weight from bootstrap '**Er = nb/Nc**' for each pathway category - **Er (enrichment by random)**. Enriched for synergy over random if **Es > Er**. Code for this analysis is included in the publication’s Github repository.

### Additional Cell Culture

WSU-DLCL2 cells are maintained in RPMI-160 (Gibco) supplemented with 10% (v:v) heat-inactivated fetal bovine serum (Sigma Aldrich; Cat. No. F4135), 2 mM L-Glutamine, and 50 U/mL Penicillin-Streptomycin.

NOMO1 cells were obtained from DSMZ and maintained in RPMI-160 (Gibco) supplemented with 10% fetal bovine serum and 5% L-Glutamine.

AN3-CA cells were obtained from ATCC and maintained with DMEM supplemented with 1-% FBS and 1% L-Glutamine. HEC1 were obtained from ATCC and maintained with McCoy’s 5a Medium Modified supplemented with 10% FBS. MFE-280 were obtained by ECACC and MFE-296 were obtained from DSMZ. Both cells were maintained in MEM with 10% FBS and 1% L-Glutamine. All cells were incubated at 37 °C under 5% CO2. All cell lines were authenticated and tested negative for mycoplasma contamination.

### Drug treatments and cell assays

For AZD2811 in combination with venetoclax studies, cells were seeded at 0.5E6 cells/mL in culture medium containing either 50 μM Q-VD-OPH (Cayman Chemical; item no. 15260) pan-caspase inhibitor or vehicle 16 hours prior to dosing with compounds. Compounds were solubilized in DMSO at a stock concentration of 10 mM and diluted in sterile PBS to a 10X solution. Falcon 96-well White Flat Bottom plates (Corning; cat. No. 353296) were seeded with 10X compounds and cells were added on top for a final assay volume of 100 μL/well. 5 μM Staurosporine (Sigma Aldrich; cat. No. S5921) was used as a positive control for cell death. After 72 hours, 50 μL of CellTiter Glo (Promega; cat. No. G7572) was added on top of the cells, the plates were shaken for 2 minutes, and then left to incubate protected from light at room temperature for 30 minutes before reading luminescence on the Synergy Neo2 (BioTek) plate reader.

The Caspase-Glo-3/7 time course assay was conducted similarly to the 3-day growth assay, except that 100 μL/well of Caspase-Glo-3/7 (Promega; cat. No. G8090) was added onto the cells at the time point. Separate plates were used for each time point.

For the AKT inhibitor capivasertib in combination with the MCL1 inhibitor AZD5991 studies cells were seeded overnight on white opaque plates (384w; Corning) at 2500-5000 cells per well in a 30μL volume. Combinations were dosed using 5-point half-log dilutions at indicated doses using an Echo 555 acoustic liquid dispenser (Labcyte). Cell viability was measured at indicated time points after drug incubation using CellTiter-Glo (Promega). The percentage of viability was calculated by normalizing drug-induced luminescent measurements to a negative control (DMSO only). For pre-treatment studies, DMSO or QVD was added to the media when cells were seeded; 16 hours later the combination was added and cell viability was measured at indicated time points.

For measurement of caspase activation, cells were incubated with compounds for 6 hours followed by addition of Caspase-Glo 3/7 (Promega) following the vendor-supplied protocol. The percentage of caspase activation was calculated by normalizing drug-induced luminescent measurements to maximum (100% mixture of 0.5 mM AZD5991 and 0.5 mM AZD4320 inhibitors) and minimum (dimethyl sulfoxide [DMSO] only) controls.

For drug treatments used for light microscopy or harvesting protein, cells were seeded overnight in 6-well plates (Corning) at 30-80% confluency. Compounds were manually added at indicated doses and harvested at indicated time points.

For the cell viability 6 x 6 drug combination matrix studies 1000 – 2000 cells were seeded in 384 black well plates (Greiner Bio-One Ltd, Stonehouse, #781090) and incubated overnight at 37 °C, 5% CO2. Cells were dosed using an Echo 555 acoustic liquid dispenser (Labcyte). Cell viability was measured at the time of dosing (day 0) and 72 hours after drug incubation using CellTiter-Glo (Promega) according to the manufacturer’s instruction. Cell viability values were normalized to the day 0 and the day 3 DMSO and were analyzed using Genedata Screener to generate heatmaps and calculate the HSA synergy score.

For siRNA experiments, AN3-CA (30nM siRNA) and MFE-296 (40nM siRNA) with 9000 cells per 96 well) were reverse transfected using lipofectamine RNAimax and siRNAs of a non-targeting pool (Dharmacon, D-001810-10-05) or a MCL1 pool (Dharmacon, L-004501-00-0005). After 18 hrs cells were treated with a drug dose range of 0.001 to 10uM. 72hrs later viability was measured with CTG and signal normalized to the DMSO control.

### Western Blot

Cells were collected and centrifuged before the pellet was lysed in a cold RIPPA buffer (Thermo Scientific 89901) supplemented with HALT protease and phosphatase inhibitor cocktail (Thermo Fisher; Cat. No. 78440). Samples were prepared with NuPAGE LDS Sample Buffer (4X) (Thermo Fisher; Cat. No. NP0007) and boiled at 95°C for 5 minutes. Protein samples were quantified using Pierce BCA Protein Assay Kit (Thermo scientific 23225) according to the manufacturer instruction before equal amounts of proteins were loaded and separated on to 4-12% NuPAGE or Bolt Bis-Tris protein gels, transferred to nitrocellulose membranes and blocked with 5% (wt/vol) nonfat dry milk in TBST (20 mM Tris-HCl (pH 7.6), 137 mM NaCl, 0.1% Tween-20). Membranes were probed with indicated primary antibodies overnight at 4 °C. HRP-conjugated secondary antibodies (CST 7074) (1:2000) were diluted in 5% (wt/vol) nonfat dry milk in TBST and detected on autoradiographic films or using G:Box gel doc system (Syngene) or Amersham ImageQuant 800 imager (Cytiva) after incubating with the ECL or SuperSignal West Dura reagents (Pierce).

### Antibodies

The following antibodies were used in this study: pPRAS40(T246) catalog number CST 2997; pAKT(S473) CST9271; total AKT CST 4691; cleaved PARP CST 9542; cleaved caspase 3 CST 9664; vinculin Sigma V9131; GAPDH CST 2118; pERK CST 9101; BIM CST 2933; β-Tubulin CST 2146; MCL1 CST 5453.

### Xenograft Efficacy Studies

All experimental work involving the use of laboratory animals was conducted in accordance with the recommendations set forth in the Guide for the Care and Use of Laboratory Animals, 8th edition. Mice were housed under pathogen-free conditions in individual ventilated cages at the AAALAC (Association for the Assessment and Accreditation of Laboratory Animal Care) accredited facilities at AstraZeneca (Waltham, MA) or Champions Oncology (Rockville, MD). All studies were reviewed and approved by the respective Institutional Animal Care and Use Committees (IACUC); work at Champions Oncology was also reviewed for compliance with AZ’s global ethics standards. All results were reported following the Animal Research: Reporting In Vivo experiments guidelines.

C.B.-17 scid (severe combined immunodeficient) mice were purchased from Charles River Laboratories (Wilmington, MA) for the WSU-DLCL2 study or Taconic (Germantown, NY) for the NOMO-1 study. 5-8 week old mice were implanted with either five million luciferase tagged WSU-DLCL2 tumor cells (WSU-DLCL2luc) or two million NOMO-1 cells with 50% matrigel (Corning). Tumor volumes (measured by caliper), animal body weight, and tumor condition were recorded twice weekly for the duration of the study. The tumor volume was calculated using the formula: length (mm) x width (mm)^2^/0.52. Tumor growth inhibition from the start of treatment was assessed by comparison of the differences in tumor volume between control and treated groups

Statistical significance was evaluated using a two-way ANOVA with Tukey’s Test. Statistical significance is identified as follows: * 0.05 < p < 0.01, ** 0.01 < p < 0.001. For efficacy studies, mice were randomized based on tumor volumes using stratified sampling, and enrolled into control and treatment groups.

### Software

Figures 1A and 3A were created using a licensed version of BioRender.com. Figures 4A, 4B, 5A, 6A were generated using TIBCO Spotfire Analyze. Matrices in Figures 6C, 6D, 6F and S8A-C, S9A-C, S11A-B, S13A-B, S14A-B, and S15A were generated in Genedata Screener.

## Supplementary Material

Supplementary figures

Supplementary table legend

Table S1

Table S2

Table S3

Table S4

Table S5

Table S6

Table S7

Table S8

Table S9

Table S10

Table S11

Table S12

Table S13

Table S14

Table S15

Table S16

Table S17

Table S18

## Data Availability

Analysis code for the manuscript figures, biomarkers and screening data fitting are available in the following repositories: https://github.com/eac54/Large-scale-pan-cancer-screening https://github.com/CancerRxGene/gdscmatrixanalyser

## Statement of significance

We present the largest cancer drug combination screen published to date with 7x7 concentration response matrices for 109 combinations in over 750 cell lines, complemented by multi-omics predictors of response and identification of ‘emergent’ combination biomarkers. We prioritize hits to optimize clinical translatability, and experimentally validate novel combination hypotheses.

## Data Availability

Screening data is available through Figshare (https://figshare.com/projects/Large-scale_pan-cancer_cell_line_screening_identifies_actionable_and_effective_drug_combinations/163378). Combination response data is available via the GDSC Combinations Website (https://gdsc-combinations.depmap.sanger.ac.uk/). Combination response data is visualized and explored at the screen, cancer type, combination and cell line-combination level. Additionally, there are links to other widely-used cancer pharmacogenomics resources such as CancerRXGene (61), Cell Model Passports (11,61), Depmap (38) and Project SCORE (62), enabling researchers to fully explore their combinations and cancer type of interest in the context of other large public pharmacogenomic datasets.

## References

[1] Devita VT, Serpick AA, Carbone PP (1970). Combination chemotherapy in the treatment of advanced Hodgkin’s disease. Ann Intern Med.

[2] DeVita VT (2003). A selective history of the therapy of Hodgkin’s disease. Br J Haematol.

[3] Boshuizen J, Peeper DS (2020). Rational Cancer Treatment Combinations: An Urgent Clinical Need. Mol Cell.

[4] Morgan P, Brown DG, Lennard S, Anderton MJ, Barrett JC, Eriksson U (2018). Impact of a five-dimensional framework on R&D productivity at AstraZeneca. Nat Rev Drug Discov.

[5] Cook D, Brown D, Alexander R, March R, Morgan P, Satterthwaite G (2014). Lessons learned from the fate of AstraZeneca’s drug pipeline: a five-dimensional framework. Nat Rev Drug Discov.

[6] Menden MP, Wang D, Mason MJ, Szalai B, Bulusu KC, Guan Y (2019). Community assessment to advance computational prediction of cancer drug combinations in a pharmacogenomic screen. Nat Commun.

[7] Jaaks P, Coker EA, Vis DJ, Edwards O, Carpenter EF, Leto SM (2022). Effective drug combinations in breast, colon and pancreatic cancer cells. Nature.

[8] Nair NU, Greninger P, Zhang X, Friedman AA, Amzallag A, Cortez E (2023). A landscape of response to drug combinations in non-small cell lung cancer. Nat Commun.

[9] O’Neil J, Benita Y, Feldman I, Chenard M, Roberts B, Liu Y (2016). An Unbiased Oncology Compound Screen to Identify Novel Combination Strategies. Mol Cancer Ther.

[10] Close DA, Wang AX, Kochanek SJ, Shun T, Eiseman JL, Johnston PA (2019). Implementation of the NCI-60 Human Tumor Cell Line Panel to Screen 2260 Cancer Drug Combinations to Generate >3 Million Data Points Used to Populate a Large Matrix of Anti-Neoplastic Agent Combinations (ALMANAC) Database. SLAS Discov.

[11] van der Meer D, Barthorpe S, Yang W, Lightfoot H, Hall C, Gilbert J (2019). Cell Model Passports-a hub for clinical, genetic and functional datasets of preclinical cancer models. Nucleic Acids Res.

[12] Garnett MJ, Edelman EJ, Heidorn SJ, Greenman CD, Dastur A, Lau KW (2012). Systematic identification of genomic markers of drug sensitivity in cancer cells. Nature.

[13] Iorio F, Knijnenburg TA, Vis DJ, Bignell GR, Menden MP, Schubert M (2016). A Landscape of Pharmacogenomic Interactions in Cancer. Cell.

[14] Yu C, Mannan AM, Yvone GM, Ross KN, Zhang Y-L, Marton MA (2016). High-throughput identification of genotype-specific cancer vulnerabilities in mixtures of barcoded tumor cell lines. Nat Biotechnol.

[15] Bliss CI (1939). The toxicity of poisons applied jointly 1. Ann Appl Biol.

[16] Berenbaum MC (1985). The expected effect of a combination of agents: the general solution. J Theor Biol.

[17] Tao Z-F, Hasvold L, Wang L, Wang X, Petros AM, Park CH (2014). Discovery of a Potent and Selective BCL-XL Inhibitor with in Vivo Activity. ACS Med Chem Lett.

[18] Speranza G, Kinders RJ, Khin S, Weil MK, Do KT, Horneffer Y (2012). Pharmacodynamic biomarker-driven trial of MK-2206, an AKT inhibitor, with AZD6244 (selumetinib), a MEK inhibitor, in patients with advanced colorectal carcinoma (CRC). J Clin Oncol.

[19] Zaman S, Wang R, Gandhi V (2014). Targeting the apoptosis pathway in hematologic malignancies. Leuk Lymphoma.

[20] Cokelaer T, Chen E, Iorio F, Menden MP, Lightfoot H, Saez-Rodriguez J (2018). GDSCTools for mining pharmacogenomic interactions in cancer. Bioinformatics.

[21] Garcia-Alonso L, Iorio F, Matchan A, Fonseca N, Jaaks P, Peat G (2018). Transcription Factor Activities Enhance Markers of Drug Sensitivity in Cancer. Cancer Res.

[22] Griffith M, Spies NC, Krysiak K, McMichael JF, Coffman AC, Danos AM (2017). CIViC is a community knowledgebase for expert crowdsourcing the clinical interpretation of variants in cancer. Nat Genet.

[23] Parker JS, Mullins M, Cheang MCU, Leung S, Voduc D, Vickery T (2009). Supervised risk predictor of breast cancer based on intrinsic subtypes. J Clin Oncol.

[24] Ebbert MTW, Bastien RRL, Rowe LR, Miller PA, Anderson D, Boucher KM (2011). PAM50 breast cancer intrinsic classifier: Clinical validation of a multianalyte laboratory developed test. J Clin Orthod.

[25] Prahallad A, Sun C, Huang S, Di Nicolantonio F, Salazar R, Zecchin D (2012). Unresponsiveness of colon cancer to BRAF(V600E) inhibition through feedback activation of EGFR. Nature.

[26] Corcoran RB, Ebi H, Turke AB, Coffee EM, Nishino M, Cogdill AP (2012). EGFR-mediated re-activation of MAPK signaling contributes to insensitivity of BRAF mutant colorectal cancers to RAF inhibition with vemurafenib. Cancer Discov.

[27] Connolly K, Brungs D, Szeto E, Epstein RJ (2014). Anticancer activity of combination targeted therapy using cetuximab plus vemurafenib for refractory BRAF (V600E)-mutant metastatic colorectal carcinoma. Curr Oncol.

[28] Xie Z, Bailey A, Kuleshov MV, Clarke DJB, Evangelista JE, Jenkins SL (2021). Gene Set Knowledge Discovery with Enrichr. Curr Protoc.

[29] Hubner SE, de Camargo Magalhães ES, Hoff FW, Brown BD, Qiu Y, Horton TM (2023). DNA Damage Response-Related Proteins Are Prognostic for Outcome in Both Adult and Pediatric Acute Myelogenous Leukemia Patients: Samples from Adults and from Children Enrolled in a Children’s Oncology Group Study. Int J Mol Sci.

[30] Adhikary U, Paulo JA, Godes M, Roychoudhury S, Prew MS, Ben-Nun Y (2023). Targeting MCL-1 triggers DNA damage and an anti-proliferative response independent from apoptosis induction. Cell Rep.

[31] Carter JL, Hege K, Yang J, Kalpage HA, Su Y, Edwards H (2020). Targeting multiple signaling pathways: the new approach to acute myeloid leukemia therapy. Signal Transduct Target Ther.

[32] Jain N, Curran E, Iyengar NM, Diaz-Flores E, Kunnavakkam R, Popplewell L (2014). Phase II study of the oral MEK inhibitor selumetinib in advanced acute myelogenous leukemia: a University of Chicago phase II consortium trial. Clin Cancer Res.

[33] Lauchle JO, Kim D, Akagi K, Crone M, Krisman K (2009). Response and resistance to MEK inhibition in leukaemias initiated by hyperactive Ras. Nature.

[34] McMahon CM, Ferng T, Canaani J, Wang ES, Morrissette JJD, Eastburn DJ (2019). Clonal Selection with RAS Pathway Activation Mediates Secondary Clinical Resistance to Selective FLT3 Inhibition in Acute Myeloid Leukemia. Cancer Discov.

[35] Shah OJ, Lin X, Li L, Huang X, Li J, Anderson MG (2010). Bcl-XL represents a druggable molecular vulnerability during aurora B inhibitor-mediated polyploidization. Proc Natl Acad Sci U S A.

[36] Brown FC, Urosevic J, Polanska U, Cosaert J, Pease JE, Pomilio G (2019). Targeting Aurora kinase B with AZD2811 enhances venetoclax activity in TP53-mutant AML. Blood.

[37] Holbeck SL, Camalier R, Crowell JA, Govindharajulu JP, Hollingshead M, Anderson LW (2017). The National Cancer Institute ALMANAC: A Comprehensive Screening Resource for the Detection of Anticancer Drug Pairs with Enhanced Therapeutic Activity. Cancer Res.

[38] Boehm JS, Garnett MJ, Adams DJ, Francies HE, Golub TR, Hahn WC (2021). Cancer research needs a better map. Nature.

[39] Gonçalves E, Poulos RC, Cai Z, Barthorpe S, Manda SS, Lucas N (2022). Pan-cancer proteomic map of 949 human cell lines. Cancer Cell.

[40] Nusinow DP, Szpyt J, Ghandi M, Rose CM, McDonald ER, Kalocsay M (2020). Quantitative Proteomics of the Cancer Cell Line Encyclopedia. Cell.

[41] Liu F, Zhao Q, Su Y, Lv J, Gai Y, Liu S (2022). Cotargeting of Bcl-2 and Mcl-1 shows promising antileukemic activity against AML cells including those with acquired cytarabine resistance. Exp Hematol.

[42] Dunn S, Eberlein C, Yu J, Gris-Oliver A, Ong SH, Yelland U (2022). AKT-mTORC1 reactivation is the dominant resistance driver for PI3Kβ/AKT inhibitors in PTEN-null breast cancer and can be overcome by combining with Mcl-1 inhibitors. Oncogene.

[43] Plana D, Palmer AC, Sorger PK (2022). Independent Drug Action in Combination Therapy: Implications for Precision Oncology. Cancer Discov.

[44] Ianevski A, Giri AK, Gautam P, Kononov A, Potdar S, Saarela J (2019). Prediction of drug combination effects with a minimal set of experiments. Nat Mach Intell.

[45] Zagidullin B, Aldahdooh J, Zheng S, Wang W, Wang Y, Saad J (2019). DrugComb: an integrative cancer drug combination data portal. Nucleic Acids Res.

[46] Seo H, Tkachuk D, Ho C, Mammoliti A, Rezaie A, Madani Tonekaboni SA (2020). SYNERGxDB: an integrative pharmacogenomic portal to identify synergistic drug combinations for precision oncology. Nucleic Acids Res.

[47] Liu Y, Hu B, Fu C, Chen X (2010). DCDB: drug combination database. Bioinformatics.

[48] Wong DJL, Robert L, Atefi MS, Lassen A, Avarappatt G, Cerniglia M (2014). Antitumor activity of the ERK inhibitor SCH772984 [corrected] against BRAF mutant, NRAS mutant and wild-type melanoma. Mol Cancer.

[49] Angius G, Tomao S, Stati V, Vici P, Bianco V, Tomao F (2020). Prexasertib, a checkpoint kinase inhibitor: from preclinical data to clinical development. Cancer Chemother Pharmacol.

[50] Hartley JA, Flynn MJ, Bingham JP, Corbett S, Reinert H, Tiberghien A (2018). Pre-clinical pharmacology and mechanism of action of SG3199, the pyrrolobenzodiazepine (PBD) dimer warhead component of antibody-drug conjugate (ADC) payload tesirine. Sci Rep.

[51] Vis DJ, Bombardelli L, Lightfoot H, Iorio F, Garnett MJ, Wessels LF (2016). Multilevel models improve precision and speed of IC50 estimates. Pharmacogenomics.

[52] Lehár J, Zimmermann GR, Krueger AS, Molnar RA, Ledell JT, Heilbut AM (2007). Chemical combination effects predict connectivity in biological systems. Mol Syst Biol.

[53] Chakravarty D, Gao J, Phillips SM, Kundra R, Zhang H, Wang J (2017). OncoKB: A Precision Oncology Knowledge Base. JCO Precis Oncol.

[54] Martincorena I, Raine KM, Gerstung M, Dawson KJ, Haase K, Van Loo P (2017). Universal Patterns of Selection in Cancer and Somatic Tissues. Cell.

[55] Bailey MH, Tokheim C, Porta-Pardo E, Sengupta S, Bertrand D, Weerasinghe A (2018). Comprehensive Characterization of Cancer Driver Genes and Mutations. Cell.

[56] van Dyk E, Reinders MJT, Wessels LFA (2013). A scale-space method for detecting recurrent DNA copy number changes with analytical false discovery rate control. Nucleic Acids Res.

[57] Chan EM, Shibue T, McFarland JM, Gaeta B, Ghandi M, Dumont N (2019). WRN helicase is a synthetic lethal target in microsatellite unstable cancers. Nature.

[58] Stelzer G, Rosen N, Plaschkes I, Zimmerman S, Twik M, Fishilevich S (2016). The GeneCards Suite: From Gene Data Mining to Disease Genome Sequence Analyses. Curr Protoc Bioinformatics.

[59] Szklarczyk D, Kirsch R, Koutrouli M, Nastou K, Mehryary F, Hachilif R (2023). The STRING database in 2023: protein–protein association networks and functional enrichment analyses for any sequenced genome of interest. Nucleic Acids Res.

[60] Wang J, Wu M, Huang X, Wang L, Zhang S, Liu H (2022). SynLethDB 2.0: a web-based knowledge graph database on synthetic lethality for novel anticancer drug discovery. Database.

[61] Yang W, Soares J, Greninger P, Edelman EJ, Lightfoot H, Forbes S (2013). Genomics of Drug Sensitivity in Cancer (GDSC): a resource for therapeutic biomarker discovery in cancer cells. Nucleic Acids Res.

[62] Behan FM, Iorio F, Picco G, Gonçalves E, Beaver CM, Migliardi G (2019). Prioritization of cancer therapeutic targets using CRISPR-Cas9 screens. Nature.

