# Supplementary material for: Large-scale pan-cancer cell line screening identifies actionable and effective drug combinations
Source: Cancer Discov. Author manuscript; Available in PMC 2024 May 3. (PMC11061612; doi:10.1158/2159-8290.CD-23-0388)
Supplement: Supplementary figures [file EMS194010-supplement-Supplementary_figures.pdf]

Supplementary Figures

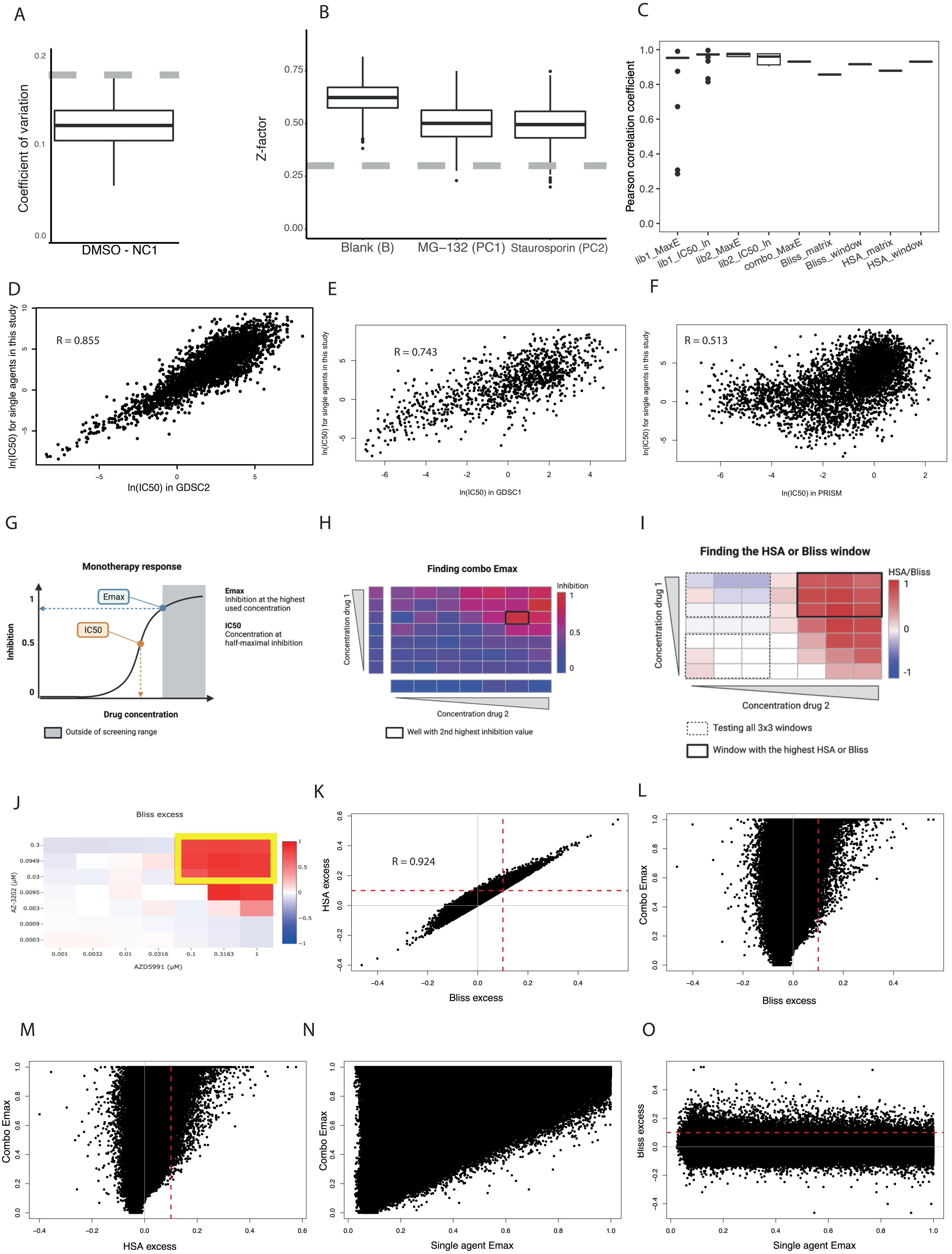
A
Coefficient of variation
0.2
0.1
0.0
DMSO - NC1
B
Z-factor
0.75
0.50
0.25
0.00
Blank (B)
MG−132 (PC1)
Staurosporin (PC2)
C
Pearson correlation coefficient
1.0
0.8
0.6
0.4
0.2
0.0
lib1_MaxE
lib1_IC50_ln
lib2_MaxE
lib2_IC50_ln
combo_MaxE
Bliss_matrix
Bliss_window
HSA_matrix
HSA_window
D
R = 0.855
ln(IC50) for single agents in this study
ln(IC50) in GDSC2
E
R = 0.743
ln(IC50) for single agents in this study
ln(IC50) in GDSC1
F
R = 0.513
ln(IC50) for single agents in this study
ln(IC50) in PRISM
G
Monotherapy response
Emax
IC50
Inhibition
Drug concentration
Emax
Inhibition at the highest used concentration
IC50
Concentration at half-maximal inhibition
Outside of screening range
H
Finding combo Emax
Inhibition
Concentration drug 1
Concentration drug 2
Well with 2nd highest inhibition value
I
Finding the HSA or Bliss window
HSA/Bliss
Concentration drug 1
Concentration drug 2
Testing all 3x3 windows
Window with the highest HSA or Bliss
J
Bliss excess
AZ-3202 (μM)
AZD5991 (μM)
K
R = 0.924
HSA excess
Bliss excess
L
Combo Emax
Bliss excess
M
Combo Emax
HSA excess
N
Combo Emax
Single agent Emax
O
Bliss excess
Single agent Emax

Supplementary Figure 1: Additional overview of screen and quality control

A: coefficient of variation boxplot for negative control wells.

B: Z-factor for positive and negative controls. MG-132 and staurosporin are positive control drugs with potent cytotoxic cell activity.

C: Correlations for replicate screenings of cell lines.

D: Schematic of how single agent IC50 and Emax are determined from fitted drug response curves

E: Schematic of experimental screening plate layout and how combination Emax is calculated

F: Schematic of how Bliss and HSA matrix and window values are obtained

G: Example of a combination matrix where there is a much higher Bliss synergy excess for the window highlighted in yellow (0.823) versus the Bliss matrix excess (0.180)

H: Scatterplot of HSA vs Bliss, showing high correlation between the two synergy metrics.

I and J: Scatterplots of correlations between Emax and Bliss (I) or HSA (J).

K: Scatter plots showing lack of correlation between combo Emax vs single agent Emax, showing the value of single agent screening.

L: Scatter plots showing lack of correlation between Bliss score vs single agent Emax, showing the value of single agent screening

M: Scatterplot of ln(IC50) of single agents in this study versus ln(IC50) of single agents in previously published GDSC2 screen showing high correlation (Pearson R = 0.855 (3dp)).

N: Scatterplot between ln(IC50) of single agents in this study versus ln(IC50) of single agents in the GDSC1 screen dataset showing good correlation (Pearson R = 0.743 (3dp)) across the 699 cell lines and two compounds screened in both projects. The screening protocol differs between the studies: Resazurin or Syto60 staining in 96- or 384-well plates for GDSC1, versus CellTitreGlo in 1536-well plates as used in this study.

O: Scatterplot between ln(IC50) of single agents in this study versus ln(IC50) of single agents in the PRISM screen with a distinctly different screening protocol, showing moderate correlation (Pearson R = 0.513 (3dp)) across the 346 cell lines and 13 compounds screened in both projects. There are multiple differences in protocol between the two studies, notably the use of barcoded pools of 20-25 cell lines in PRISM and a 5-day viability assay, versus individual cell lines and a 3-day viability assay used in this study.

A

	'High activity and synergy' in this study	Not 'high activity and synergy' in this study
Synergy in Jaaks, et al.	724 (15.1%)	369 (7.7%)
Not synergy in Jaaks, et al.	1,283 (26.8%)	2,414 (50.3%)

B

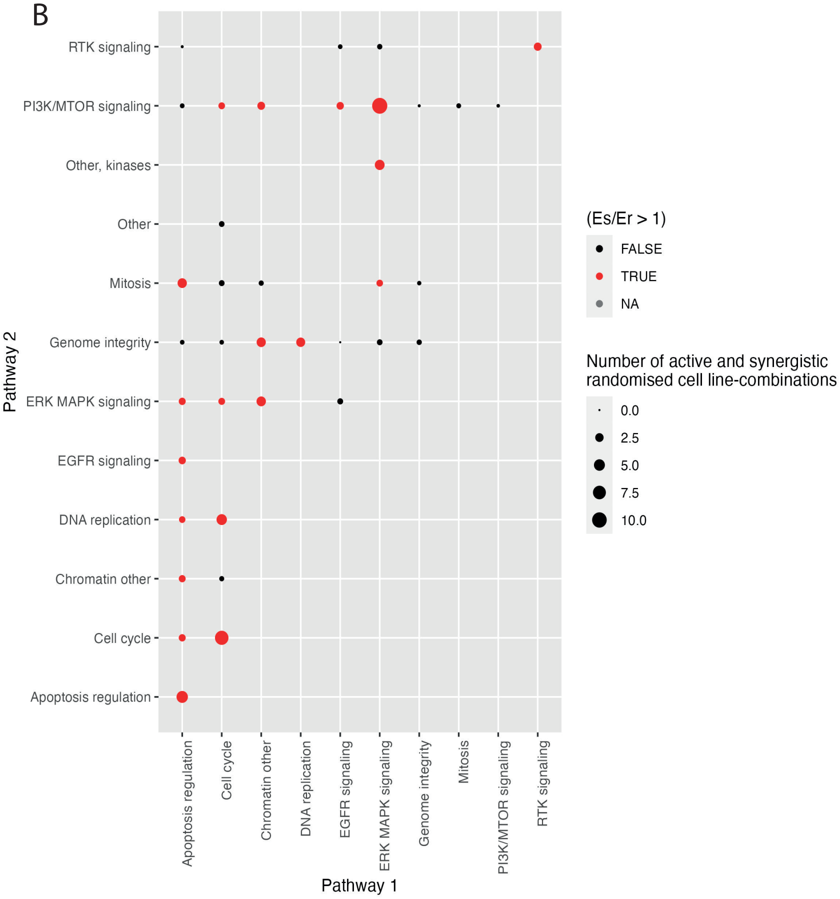

C

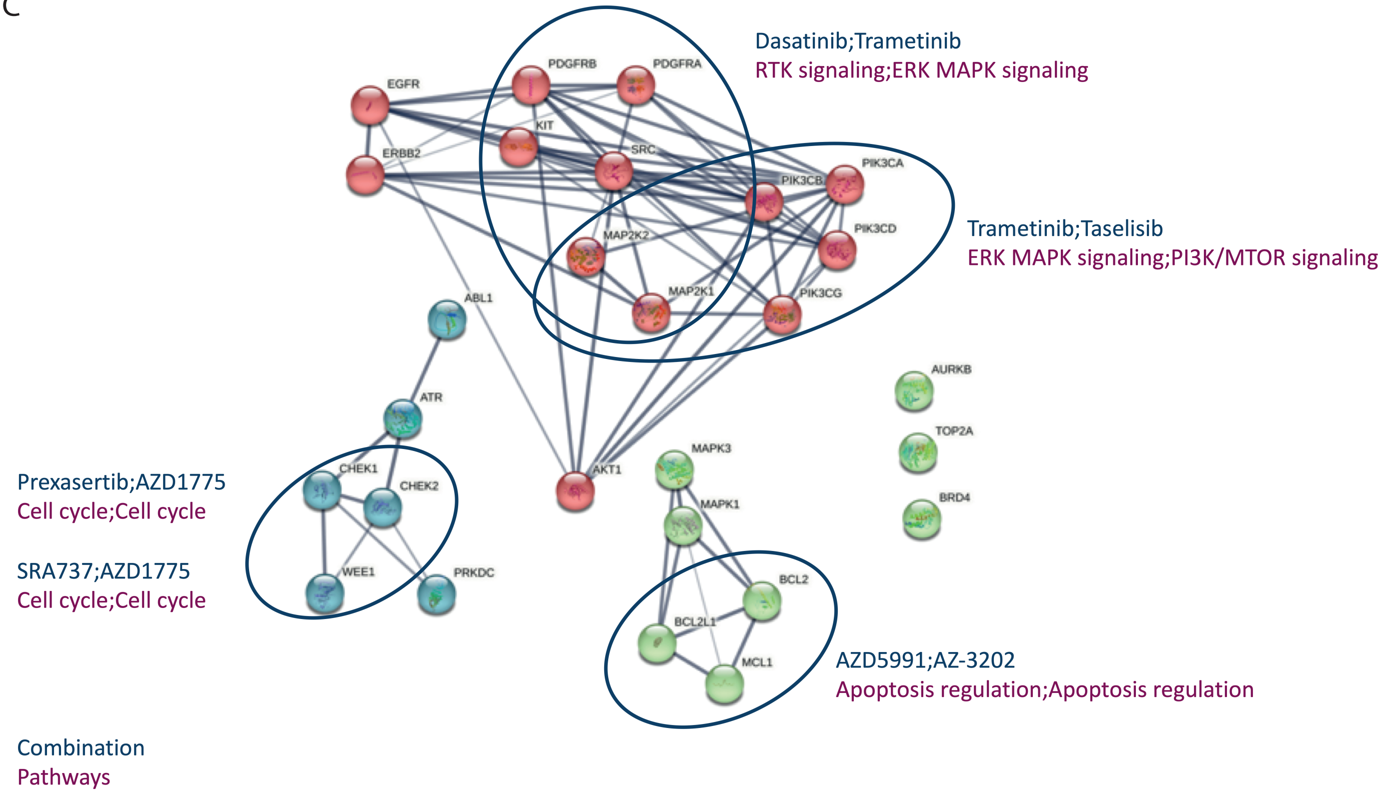

Supplementary Figure 2: Combination benefit in combination-cell line pairs

A: Comparison of classification of 4,790 combination-cell line pairs from 9 combination and 114 cell lines screened in both this study and another study (Jaaks P, et al. Nature 2022). 15.1% of combination-cell line pairs are classified as hits in both studies, and 50.3% of combination-cell line pairs are classified as non-hits in both studies. We observe good concordance despite use of differing study designs and synergy metrics.

B: Scatterplot of drug pathway-combination-cell line pairs enriched for combination benefit. Es/Er is the ratio between proportion of combinations with combination benefit over the proportion of random combinations. A ratio of >1 signifies enrichment.

C: Protein interaction map (derived from STRINGdb) of all broadly synergistic targets and their corresponding combinations. AZD5991 + AZ-3202, prexasertib + AZD1775 and SRA373 + AZD1775 are intra-pathway combinations within the apoptosis regulation and cell cycle pathways. The target genes of combinations AZD5991 + AZ-3203, AZD5153 + selumetinib and trametinib + taselisib have high synthetic lethal confidence scores.

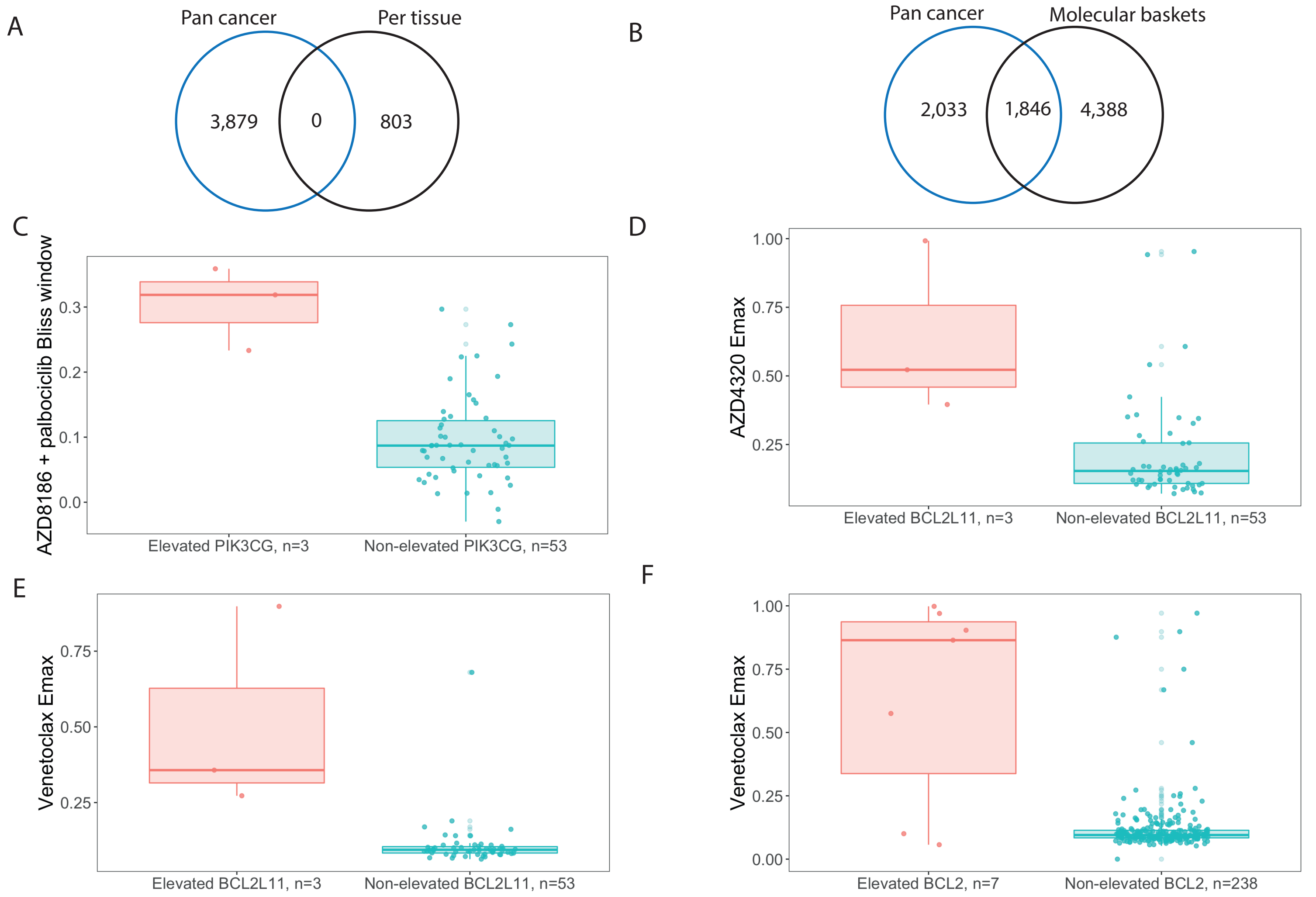

G

Pathway	CD+CD	DDR+DDR	CS+CS	CS+CD	CS+DDR	CD+DDR	CD+chemo/ other	DDR+chemo/ other	CS+chemo/ other
Allograft rejection		**	***	***	***	**	***	**	*
Androgen response		*	**		**	*	*	*	
Angiogenesis				*					
Apical junction		***	***	***	***	***	**	**	
Apical surface			*	**	***		**	**	
Apoptosis	***	***	***	***	***	***	***	***	
Coagulation		*						*	
Complement	*	***	***	***		***	**	***	*
DNA repair	*			*		*			
E2F targets	***	***	***	***	***	***	***	***	
Epithelial mesenchymal transition		***	**	***		**	***		
Estrogen response early	**	***	***	***	***	***	***	**	
Estrogen response late	*	*	***	***	**	**	**	*	
G2-M checkpoint	***	***	***	***	***	***	***	***	*
Glycolysis		**	***	***	***	**	***	**	
Hedgehog signaling		**		**	*		*	*	
Hypoxia	**	***	***	***	***	***	***	***	
IL-2/STAT5 signaling	***	**	***	***	***	***	***	***	
IL-6/JAK/STAT3 signaling	***	**	***	***	**	***	***	***	
Inflammatory response		***	*	***	*	*	*	**	*
Interferon gamma response		**	**			*		*	
KRAS signaling up	*	***	***	***	***	***	***	***	*
Mitotic spindle	***	***	*	***	***	***	**	***	
mTORC1 signaling		*	*	*			*		*
Myc targets V1							*		
Myogenesis		***	*	*	**	***		*	
Notch signaling	**	*			***				
p53 pathway	***	***	***	***	***	***	***		
PI3K/AKT/mTOR signaling	**	***	***	***	***	***	***	**	*
Protein secretion		*						*	
Spermatogenesis					*			*	
TGF-beta signaling	*	***	***	***	***	***	***	***	
TNF-alpha signaling via NF-kB	**	***	***	***	***	***	***	**	*
Unfolded protein response				**					
UV response down	*	**	***	***	***	***	***	***	
UV response up	**	***	***	***	***	***	***	***	
Wnt-beta catenin signaling	**	**	***	**	***	***	***	**	
Xenobiotic metabolism		*							

* $P < 0.05$ * $P < 0.01$ * $P < 0.001$

Supplementary Figure 3: Biomarkers of single agent and combination activity

A: Overlap of significant large effect biomarkers from pan-cancer and per cancer-type analyses. B: Overlap of significant large effect biomarkers from pan-cancer and pan-cancer molecular basket analyses. C - F: Example combination biomarkers that match the target or target pathway of one of the drugs.

G: Enriched pathways for all biomarkers in each drug combination category based on adjusted p-values: * $0.05 < p < 0.01$, ** $0.01 < p < 0.001$, *** $p < 0.001$ (CD = cell death, CS = cell signaling, chemo = chemotherapeutic agents, DDR = DNA damage response).

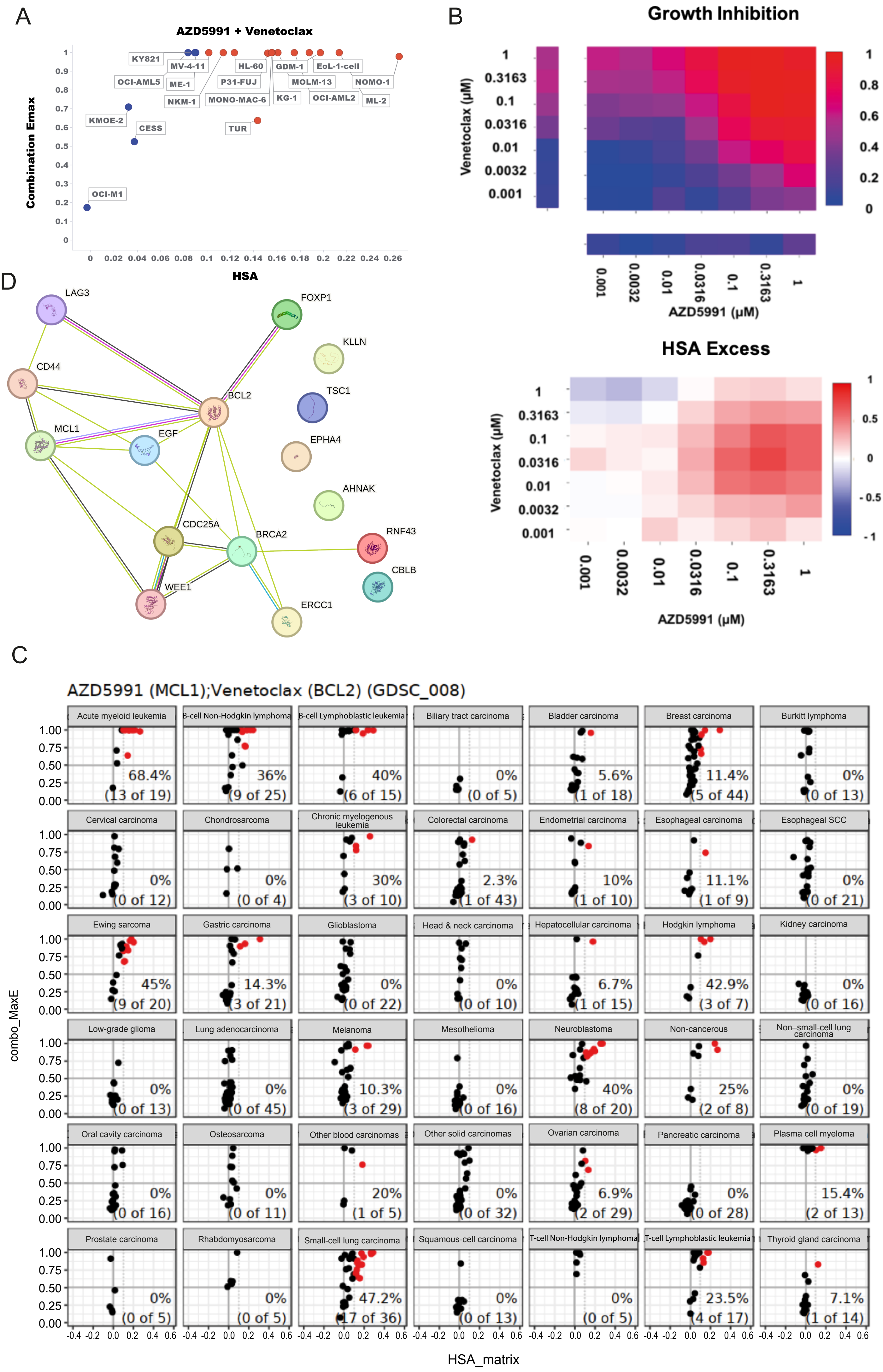
A
AZD5991 + Venetoclax
Combination Emax
HSA
KY821
MV-4-11
OCI-AML5
ME-1
NKM-1
HL-60
P31-FUJ
MONO-MAC-6
GDM-1
KG-1
MOLM-13
EoL-1-cell
OCI-AML2
NOMO-1
ML-2
KMOE-2
CESS
TUR
OCI-M1
B
Growth Inhibition
Venetoclax (μM)
AZD5991 (μM)
HSA Excess
D
LAG3
FOXP1
KLLN
CD44
TSC1
BCL2
MCL1
EGF
EPHA4
AHNAK
CDC25A
BRCA2
RNF43
CBLB
WEE1
ERCC1
C
AZD5991 (MCL1);Venetoclax (BCL2) (GDSC_008)
combo_MaxE
HSA_matrix
Acute myeloid leukemia 68.4% (13 of 19)
B-cell Non-Hodgkin lymphoma 36% (9 of 25)
B-cell Lymphoblastic leukemia 40% (6 of 15)
Biliary tract carcinoma 0% (0 of 5)
Bladder carcinoma 5.6% (1 of 18)
Breast carcinoma 11.4% (5 of 44)
Burkitt lymphoma 0% (0 of 13)
Cervical carcinoma 0% (0 of 12)
Chondrosarcoma 0% (0 of 4)
Chronic myelogenous leukemia 30% (3 of 10)
Colorectal carcinoma 2.3% (1 of 43)
Endometrial carcinoma 10% (1 of 10)
Esophageal carcinoma 11.1% (1 of 9)
Esophageal SCC 0% (0 of 21)
Ewing sarcoma 45% (9 of 20)
Gastric carcinoma 14.3% (3 of 21)
Glioblastoma 0% (0 of 22)
Head & neck carcinoma 0% (0 of 10)
Hepatocellular carcinoma 6.7% (1 of 15)
Hodgkin lymphoma 42.9% (3 of 7)
Kidney carcinoma 0% (0 of 16)
Low-grade glioma 0% (0 of 13)
Lung adenocarcinoma 0% (0 of 45)
Melanoma 10.3% (3 of 29)
Mesothelioma 0% (0 of 16)
Neuroblastoma 40% (8 of 20)
Non-cancerous 25% (2 of 8)
Non–small-cell lung carcinoma 0% (0 of 19)
Oral cavity carcinoma 0% (0 of 16)
Osteosarcoma 0% (0 of 11)
Other blood carcinomas 20% (1 of 5)
Other solid carcinomas 0% (0 of 32)
Ovarian carcinoma 6.9% (2 of 29)
Pancreatic carcinoma 0% (0 of 28)
Plasma cell myeloma 15.4% (2 of 13)
Prostate carcinoma 0% (0 of 5)
Rhabdomyosarcoma 0% (0 of 5)
Small-cell lung carcinoma 47.2% (17 of 36)
Squamous-cell carcinoma 0% (0 of 13)
T-cell Non-Hodgkin lymphoma 0% (0 of 5)
T-cell Lymphoblastic leukemia 23.5% (4 of 17)
Thyroid gland carcinoma 7.1% (1 of 14)

Supplementary Figure 4: Combination activity of venetoclax plus AZD5991 in AML cell lines.

A: Combination Emax versus HSA scores in 19 AML cell lines. Red dots are responder cell lines (combination Emax > 0.5 and HSA > 0.1).

B: Representative screen combination growth inhibition (top) and HSA excess matrix plots in the cell line GDM1.

C: AZD5991 plus venetoclax combination activity in 41 cancer types included in this study. Each dot represents a cell line. Cell lines showing high combination activity (Emax > 0.5 and HSA > 0.1) are in red.

D: STRING analysis of large (effect size >1) and significant (p<0.05, FDR <10) combination biomarkers in all or genetic baskets, highlighting the interactome of targets (MCL1, BCL2)and biomarkers.

A

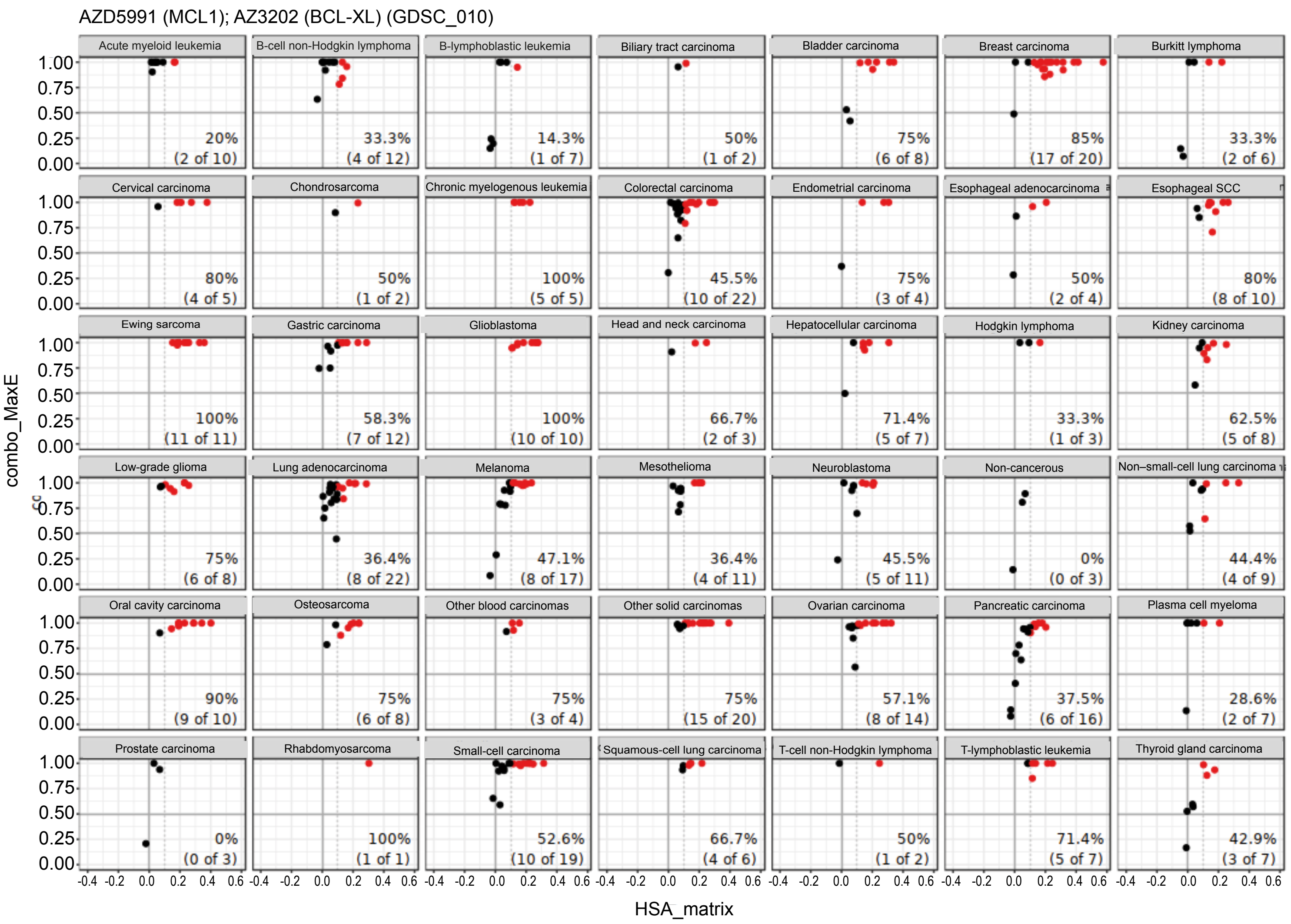

AZD5991 (MCL1); AZ3202 (BCL-XL) (GDSC_010)
combo_MaxE
HSA_matrix
Acute myeloid leukemia
20%
(2 of 10)
B-cell non-Hodgkin lymphoma
33.3%
(4 of 12)
B-lymphoblastic leukemia
14.3%
(1 of 7)
Biliary tract carcinoma
50%
(1 of 2)
Bladder carcinoma
75%
(6 of 8)
Breast carcinoma
85%
(17 of 20)
Burkitt lymphoma
33.3%
(2 of 6)
Cervical carcinoma
80%
(4 of 5)
Chondrosarcoma
50%
(1 of 2)
Chronic myelogenous leukemia
100%
(5 of 5)
Colorectal carcinoma
45.5%
(10 of 22)
Endometrial carcinoma
75%
(3 of 4)
Esophageal adenocarcinoma
50%
(2 of 4)
Esophageal SCC
80%
(8 of 10)
Ewing sarcoma
100%
(11 of 11)
Gastric carcinoma
58.3%
(7 of 12)
Glioblastoma
100%
(10 of 10)
Head and neck carcinoma
66.7%
(2 of 3)
Hepatocellular carcinoma
71.4%
(5 of 7)
Hodgkin lymphoma
33.3%
(1 of 3)
Kidney carcinoma
62.5%
(5 of 8)
Low-grade glioma
75%
(6 of 8)
Lung adenocarcinoma
36.4%
(8 of 22)
Melanoma
47.1%
(8 of 17)
Mesothelioma
36.4%
(4 of 11)
Neuroblastoma
45.5%
(5 of 11)
Non-cancerous
0%
(0 of 3)
Non–small-cell lung carcinoma
44.4%
(4 of 9)
Oral cavity carcinoma
90%
(9 of 10)
Osteosarcoma
75%
(6 of 8)
Other blood carcinomas
75%
(3 of 4)
Other solid carcinomas
75%
(15 of 20)
Ovarian carcinoma
57.1%
(8 of 14)
Pancreatic carcinoma
37.5%
(6 of 16)
Plasma cell myeloma
28.6%
(2 of 7)
Prostate carcinoma
0%
(0 of 3)
Rhabdomyosarcoma
100%
(1 of 1)
Small-cell carcinoma
52.6%
(10 of 19)
Squamous-cell lung carcinoma
66.7%
(4 of 6)
T-cell non-Hodgkin lymphoma
50%
(1 of 2)
T-lymphoblastic leukemia
71.4%
(5 of 7)
Thyroid gland carcinoma
42.9%
(3 of 7)

Supplementary Figure 5: Combination activity by cancer type for AZD5991 + AZ3202

A: AZ3202 plus AZD5991 combination activity in 41 cancer types included in this study. Each dot represents a cell line. Cell lines showing high combination activity (Emax > 0.5 and HSA > 0.1) are in red.

A

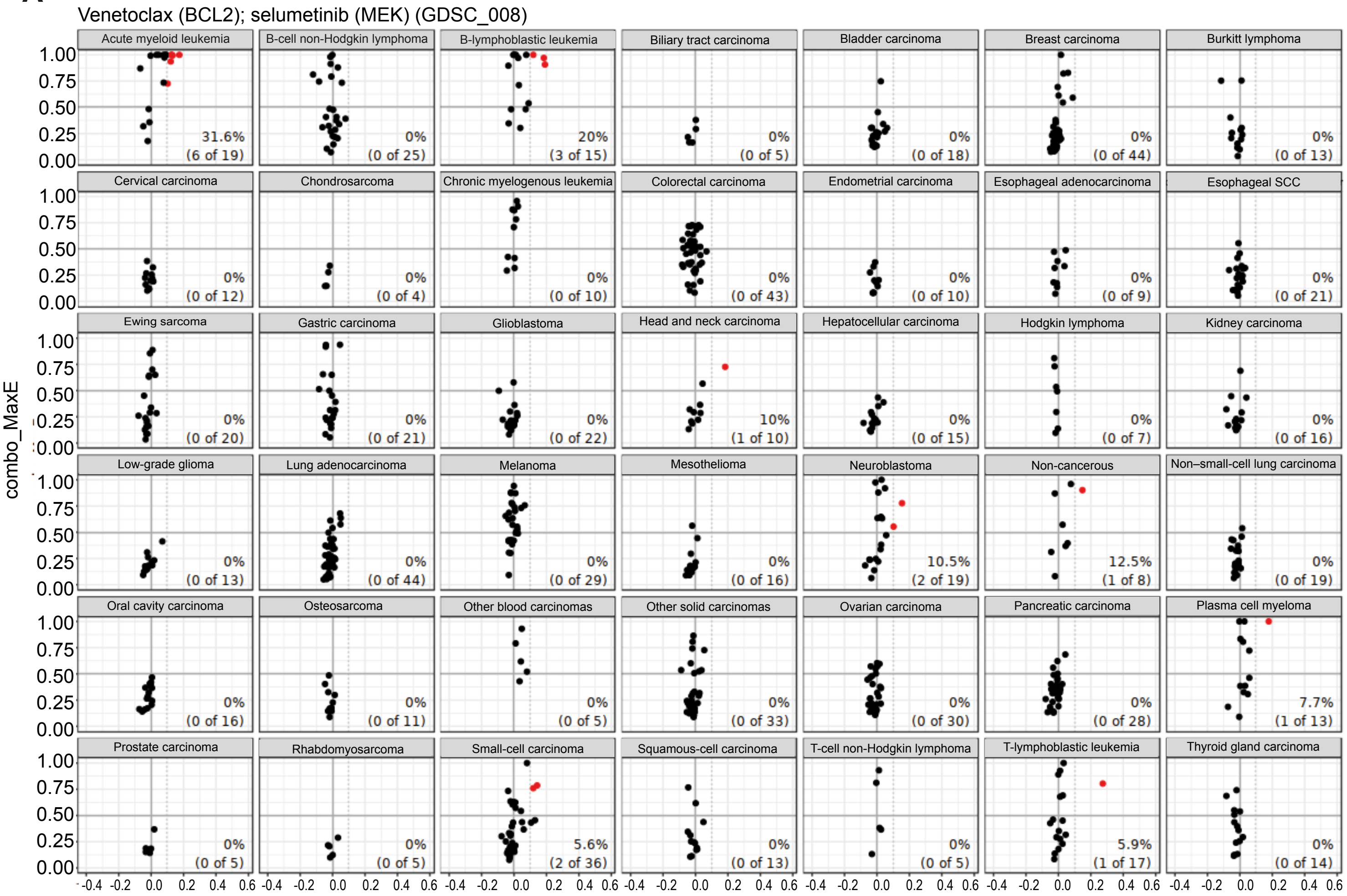

Venetoclax (BCL2); selumetinib (MEK) (GDSC_008)
combo_MaxE
Acute myeloid leukemia 31.6% (6 of 19)
B-cell non-Hodgkin lymphoma 0% (0 of 25)
B-lymphoblastic leukemia 20% (3 of 15)
Biliary tract carcinoma 0% (0 of 5)
Bladder carcinoma 0% (0 of 18)
Breast carcinoma 0% (0 of 44)
Burkitt lymphoma 0% (0 of 13)
Cervical carcinoma 0% (0 of 12)
Chondrosarcoma 0% (0 of 4)
Chronic myelogenous leukemia 0% (0 of 10)
Colorectal carcinoma 0% (0 of 43)
Endometrial carcinoma 0% (0 of 10)
Esophageal adenocarcinoma 0% (0 of 9)
Esophageal SCC 0% (0 of 21)
Ewing sarcoma 0% (0 of 20)
Gastric carcinoma 0% (0 of 21)
Glioblastoma 0% (0 of 22)
Head and neck carcinoma 10% (1 of 10)
Hepatocellular carcinoma 0% (0 of 15)
Hodgkin lymphoma 0% (0 of 7)
Kidney carcinoma 0% (0 of 16)
Low-grade glioma 0% (0 of 13)
Lung adenocarcinoma 0% (0 of 44)
Melanoma 0% (0 of 29)
Mesothelioma 0% (0 of 16)
Neuroblastoma 10.5% (2 of 19)
Non-cancerous 12.5% (1 of 8)
Non–small-cell lung carcinoma 0% (0 of 19)
Oral cavity carcinoma 0% (0 of 16)
Osteosarcoma 0% (0 of 11)
Other blood carcinomas 0% (0 of 5)
Other solid carcinomas 0% (0 of 33)
Ovarian carcinoma 0% (0 of 30)
Pancreatic carcinoma 0% (0 of 28)
Plasma cell myeloma 7.7% (1 of 13)
Prostate carcinoma 0% (0 of 5)
Rhabdomyosarcoma 0% (0 of 5)
Small-cell carcinoma 5.6% (2 of 36)
Squamous-cell carcinoma 0% (0 of 13)
T-cell non-Hodgkin lymphoma 0% (0 of 5)
T-lymphoblastic leukemia 5.9% (1 of 17)
Thyroid gland carcinoma 0% (0 of 14)

B

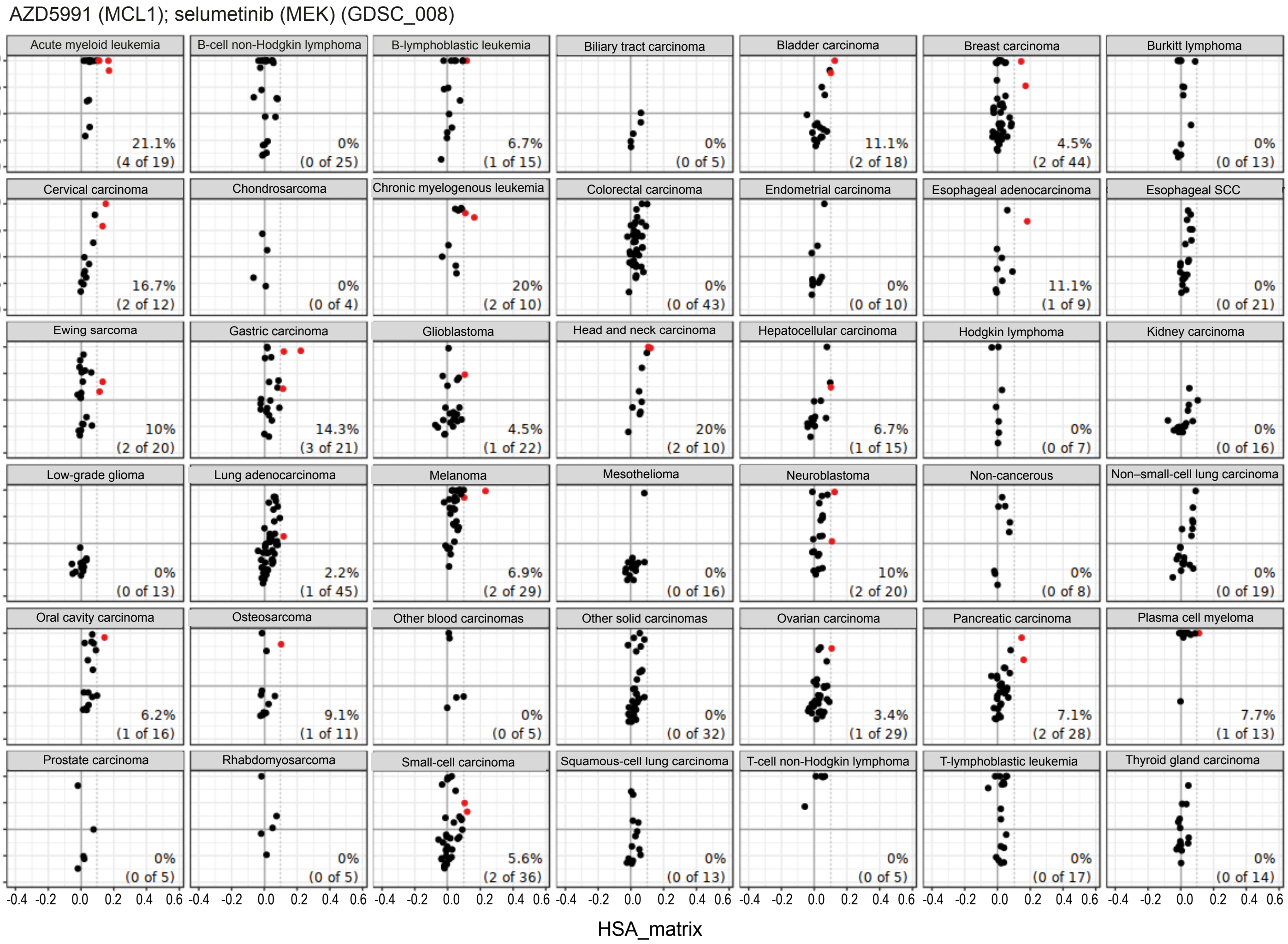

AZD5991 (MCL1); selumetinib (MEK) (GDSC_008)
combo_MaxE
HSA_matrix
Acute myeloid leukemia 21.1% (4 of 19)
B-cell non-Hodgkin lymphoma 0% (0 of 25)
B-lymphoblastic leukemia 6.7% (1 of 15)
Biliary tract carcinoma 0% (0 of 5)
Bladder carcinoma 11.1% (2 of 18)
Breast carcinoma 4.5% (2 of 44)
Burkitt lymphoma 0% (0 of 13)
Cervical carcinoma 16.7% (2 of 12)
Chondrosarcoma 0% (0 of 4)
Chronic myelogenous leukemia 20% (2 of 10)
Colorectal carcinoma 0% (0 of 43)
Endometrial carcinoma 0% (0 of 10)
Esophageal adenocarcinoma 11.1% (1 of 9)
Esophageal SCC 0% (0 of 21)
Ewing sarcoma 10% (2 of 20)
Gastric carcinoma 14.3% (3 of 21)
Glioblastoma 4.5% (1 of 22)
Head and neck carcinoma 20% (2 of 10)
Hepatocellular carcinoma 6.7% (1 of 15)
Hodgkin lymphoma 0% (0 of 7)
Kidney carcinoma 0% (0 of 16)
Low-grade glioma 0% (0 of 13)
Lung adenocarcinoma 2.2% (1 of 45)
Melanoma 6.9% (2 of 29)
Mesothelioma 0% (0 of 16)
Neuroblastoma 10% (2 of 20)
Non-cancerous 0% (0 of 8)
Non–small-cell lung carcinoma 0% (0 of 19)
Oral cavity carcinoma 6.2% (1 of 16)
Osteosarcoma 9.1% (1 of 11)
Other blood carcinomas 0% (0 of 5)
Other solid carcinomas 0% (0 of 32)
Ovarian carcinoma 3.4% (1 of 29)
Pancreatic carcinoma 7.1% (2 of 28)
Plasma cell myeloma 7.7% (1 of 13)
Prostate carcinoma 0% (0 of 5)
Rhabdomyosarcoma 0% (0 of 5)
Small-cell carcinoma 5.6% (2 of 36)
Squamous-cell lung carcinoma 0% (0 of 13)
T-cell non-Hodgkin lymphoma 0% (0 of 5)
T-lymphoblastic leukemia 0% (0 of 17)
Thyroid gland carcinoma 0% (0 of 14)

Supplementary Figure 6: Venetoclax + selumetinib and AZD5991 + selumetinib activity by cancer type.

A: Venetoclax plus selumetinib or (B) AZD5991 plus selumetinib combination activity (combination Emax versus HSA) in 41 cancer types included in this study. Each dot represents a cell line and cell lines showing high combination activity (combination Emax > 0.5 and HSA > 0.1) are in red.

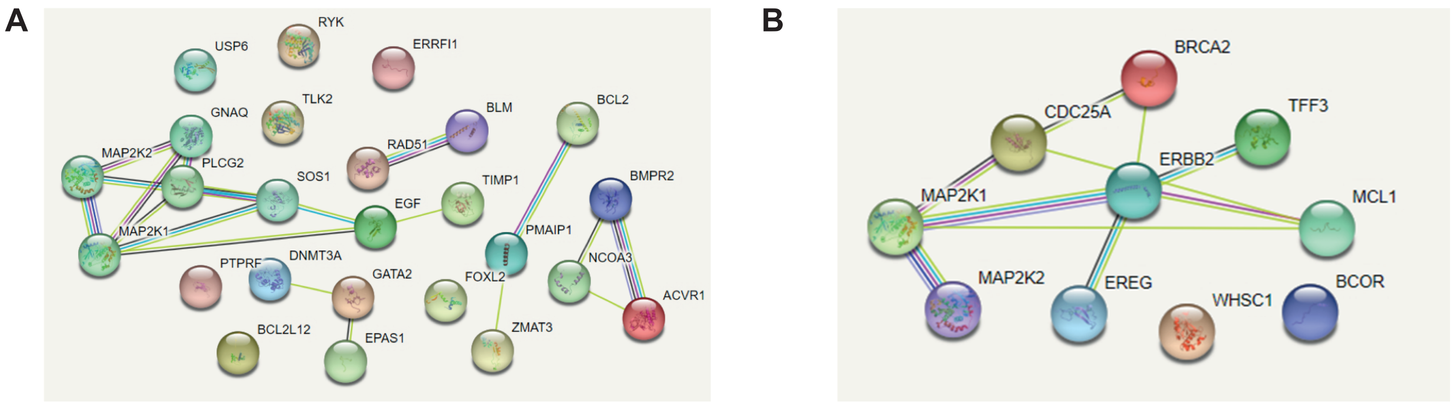
A
RYK
USP6
ERRFI1
TLK2
GNAQ
BLM
BCL2
MAP2K2
RAD51
PLCG2
SOS1
TIMP1
BMPR2
EGF
MAP2K1
PMAIP1
NCOA3
DNMT3A
PTPRF
GATA2
FOXL2
ACVR1
ZMAT3
EPAS1
BCL2L12
B
BRCA2
CDC25A
TFF3
ERBB2
MCL1
MAP2K1
MAP2K2
EREG
WHSC1
BCOR

Supplementary Figure 7: Venetoclax + selumetinib and AZD5991 + selumetinib activity by cancer type.

A: Selumetinib plus venetoclax STRING analysis of large (effect size >1) and significant (p<0.05, FDR <10) combination biomarkers in all or genetic baskets.

B: Selumetinib plus AZD5991 STRING analysis of large (effect size >1) and significant (p<0.05, FDR <10) combination biomarkers in all or genetic baskets.

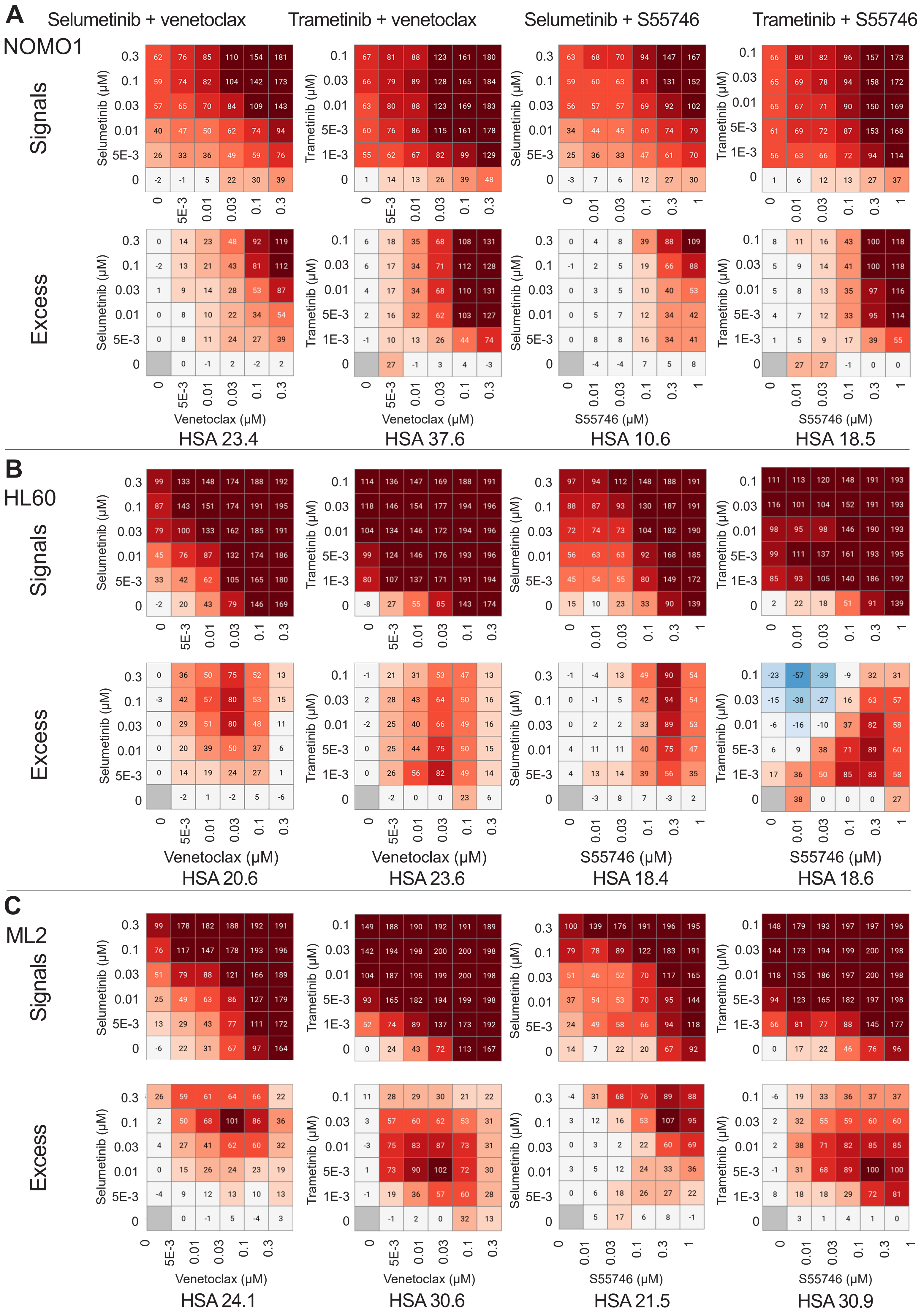
A
NOMO1
Selumetinib + venetoclax
Trametinib + venetoclax
Selumetinib + S55746
Trametinib + S55746
Signals
Excess
Selumetinib (µM)
Trametinib (µM)
Venetoclax (µM)
S55746 (µM)
HSA 23.4
HSA 37.6
HSA 10.6
HSA 18.5
B
HL60
Signals
Excess
Venetoclax (µM)
S55746 (µM)
HSA 20.6
HSA 23.6
HSA 18.4
HSA 18.6
C
ML2
Signals
Excess
Venetoclax (µM)
S55746 (µM)
HSA 24.1
HSA 30.6
HSA 21.5
HSA 30.9

Supplementary Figure 8: Effect of MEK1/2 plus BCL2 inhibition on cell viability in AML cells.

Cell viability and HSA excess following treatment for 3 days with using 6 x 6 drug combination matrix in A) NOMO1 B) HL60 and C) ML2 cells. Cells were treated with MEK1/2 inhibitors selumetinib or trametinib in combinations with BCL2 inhibitor venetoclax or BCL1 inhibitors S55746. In top panels zero represents DMSO control, values 1 - 100 represent % growth inhibition, and values > 100 represent cell death. HSA excess heatmaps indicate dose range where combination benefit was achieved.

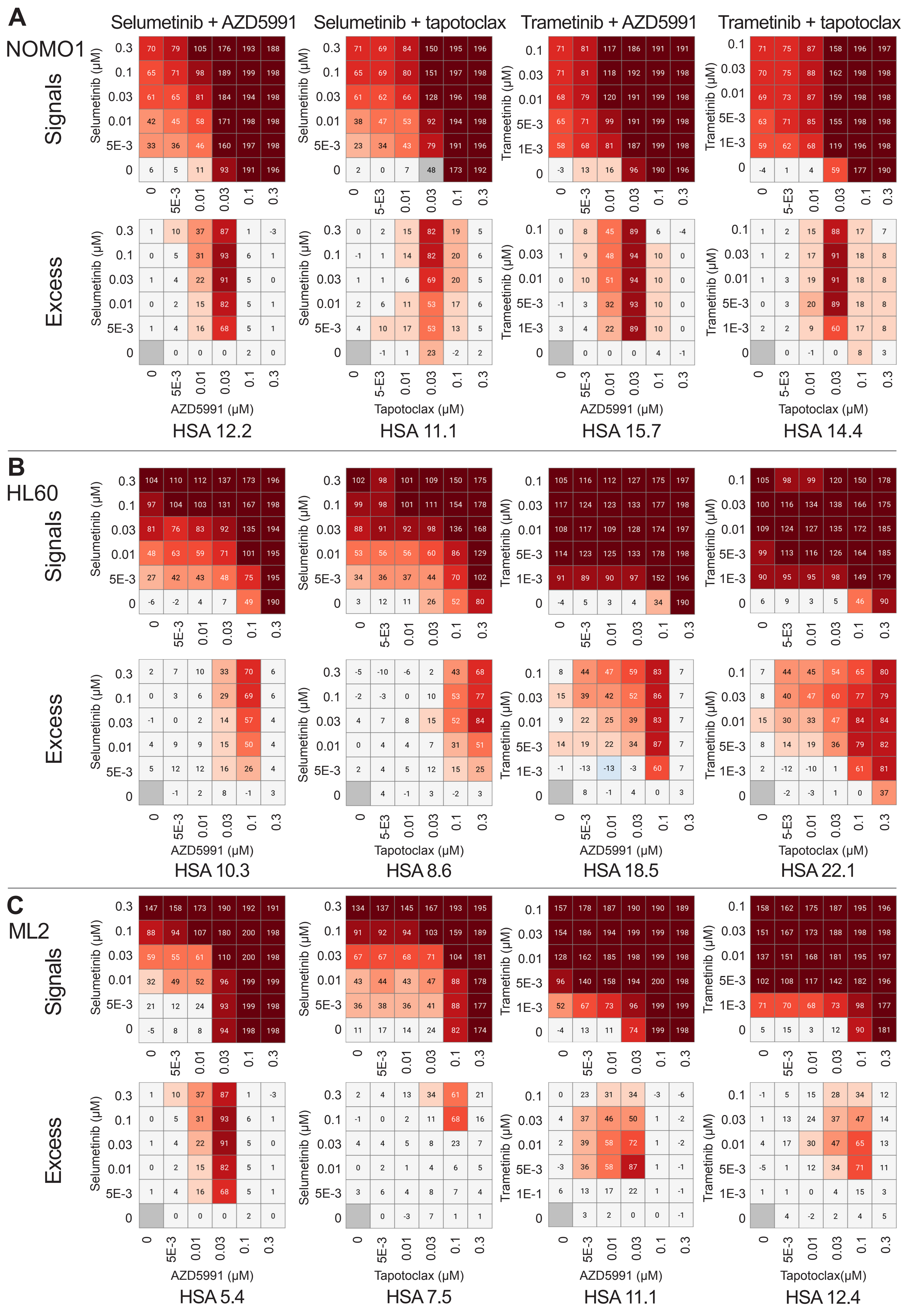
A
NOMO1
B
HL60
C
ML2
Selumetinib + AZD5991
Selumetinib + tapotoclax
Trametinib + AZD5991
Trametinib + tapotoclax
Signals
Excess
Selumetinib (μM)
Trametinib (μM)
AZD5991 (μM)
Tapotoclax (μM)
HSA 12.2
HSA 11.1
HSA 15.7
HSA 14.4
HSA 10.3
HSA 8.6
HSA 18.5
HSA 22.1
HSA 5.4
HSA 7.5
HSA 11.1
HSA 12.4

Supplementary Figure 9: Effect of MEK1/2 plus MCL1 inhibition on cell viability in AML cells.

Cell viability and HSA excess following treatment for 3 days with a 6 x 6 drug combination matrix in A) NOMO1 B) HL60 and C) ML2 cells. Cells were treated with MEK1/2 inhibitors selumetinib or trametinib in combinations with MCL1 inhibitor AZD5991 and tapotoclax. In the top panels, zero represents DMSO control, values 1 - 100 represent % growth inhibition and values > 100 represent cell death. HSA excess heatmaps indicate dose range where combination benefit was achieved

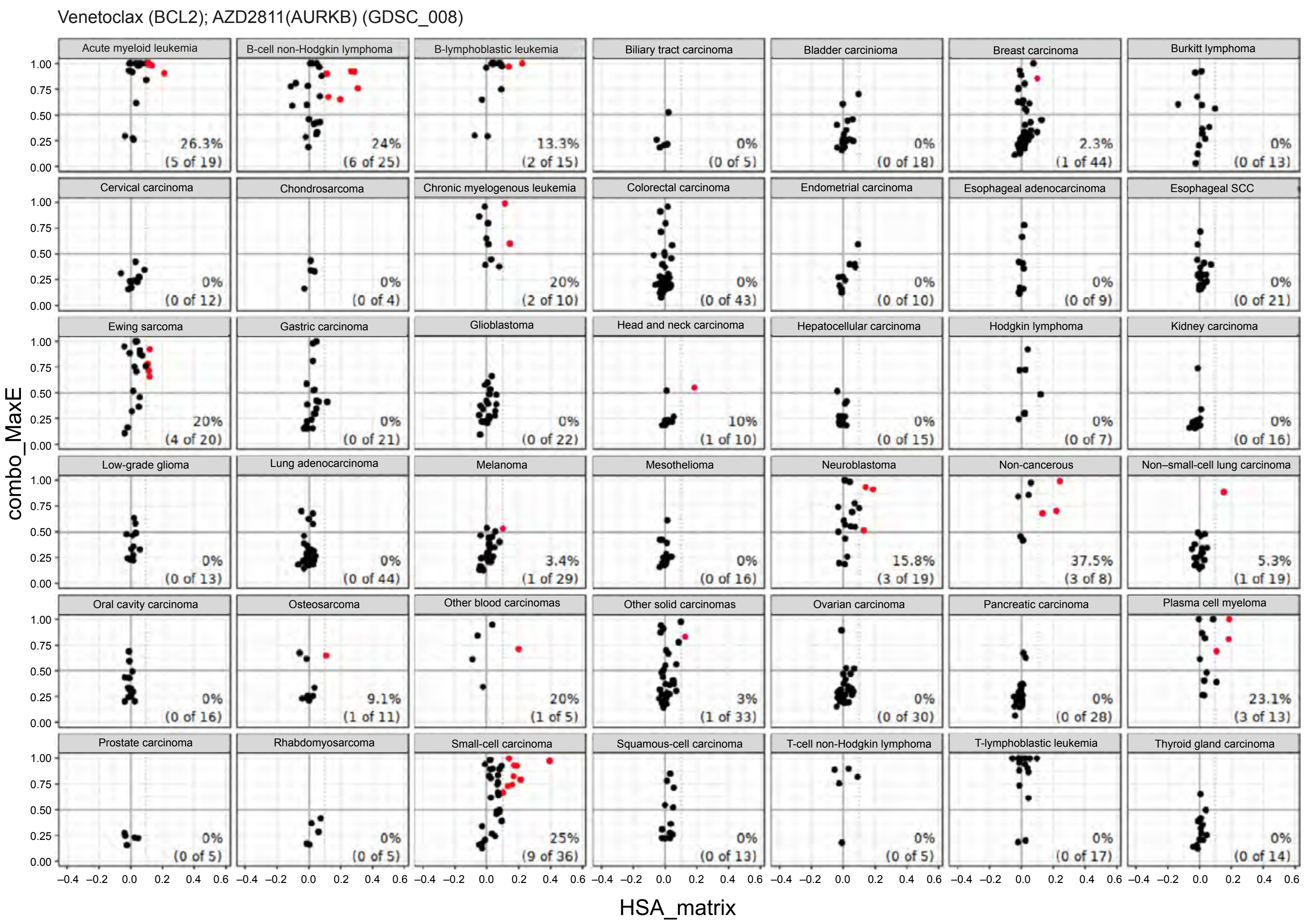
Venetoclax (BCL2); AZD2811(AURKB) (GDSC_008)
combo_MaxE
HSA_matrix
Acute myeloid leukemia 26.3% (5 of 19)
B-cell non-Hodgkin lymphoma 24% (6 of 25)
B-lymphoblastic leukemia 13.3% (2 of 15)
Biliary tract carcinoma 0% (0 of 5)
Bladder carcinioma 0% (0 of 18)
Breast carcinoma 2.3% (1 of 44)
Burkitt lymphoma 0% (0 of 13)
Cervical carcinoma 0% (0 of 12)
Chondrosarcoma 0% (0 of 4)
Chronic myelogenous leukemia 20% (2 of 10)
Colorectal carcinoma 0% (0 of 43)
Endometrial carcinoma 0% (0 of 10)
Esophageal adenocarcinoma 0% (0 of 9)
Esophageal SCC 0% (0 of 21)
Ewing sarcoma 20% (4 of 20)
Gastric carcinoma 0% (0 of 21)
Glioblastoma 0% (0 of 22)
Head and neck carcinoma 10% (1 of 10)
Hepatocellular carcinoma 0% (0 of 15)
Hodgkin lymphoma 0% (0 of 7)
Kidney carcinoma 0% (0 of 16)
Low-grade glioma 0% (0 of 13)
Lung adenocarcinoma 0% (0 of 44)
Melanoma 3.4% (1 of 29)
Mesothelioma 0% (0 of 16)
Neuroblastoma 15.8% (3 of 19)
Non-cancerous 37.5% (3 of 8)
Non–small-cell lung carcinoma 5.3% (1 of 19)
Oral cavity carcinoma 0% (0 of 16)
Osteosarcoma 9.1% (1 of 11)
Other blood carcinomas 20% (1 of 5)
Other solid carcinomas 3% (1 of 33)
Ovarian carcinoma 0% (0 of 30)
Pancreatic carcinoma 0% (0 of 28)
Plasma cell myeloma 23.1% (3 of 13)
Prostate carcinoma 0% (0 of 5)
Rhabdomyosarcoma 0% (0 of 5)
Small-cell carcinoma 25% (9 of 36)
Squamous-cell carcinoma 0% (0 of 13)
T-cell non-Hodgkin lymphoma 0% (0 of 5)
T-lymphoblastic leukemia 0% (0 of 17)
Thyroid gland carcinoma 0% (0 of 14)

Supplementary Figure 10: Cancer type selectivity of venetoclax + AZD2811.
A: Venetoclax plus AZD2811 combination activity (combination Emax versus HSA) in 41 cancer types included in this study. Each dot represents a cell line and cell lines showing high combination activity (combination Emax > 0.5 and HSA > 0.1) are in red.

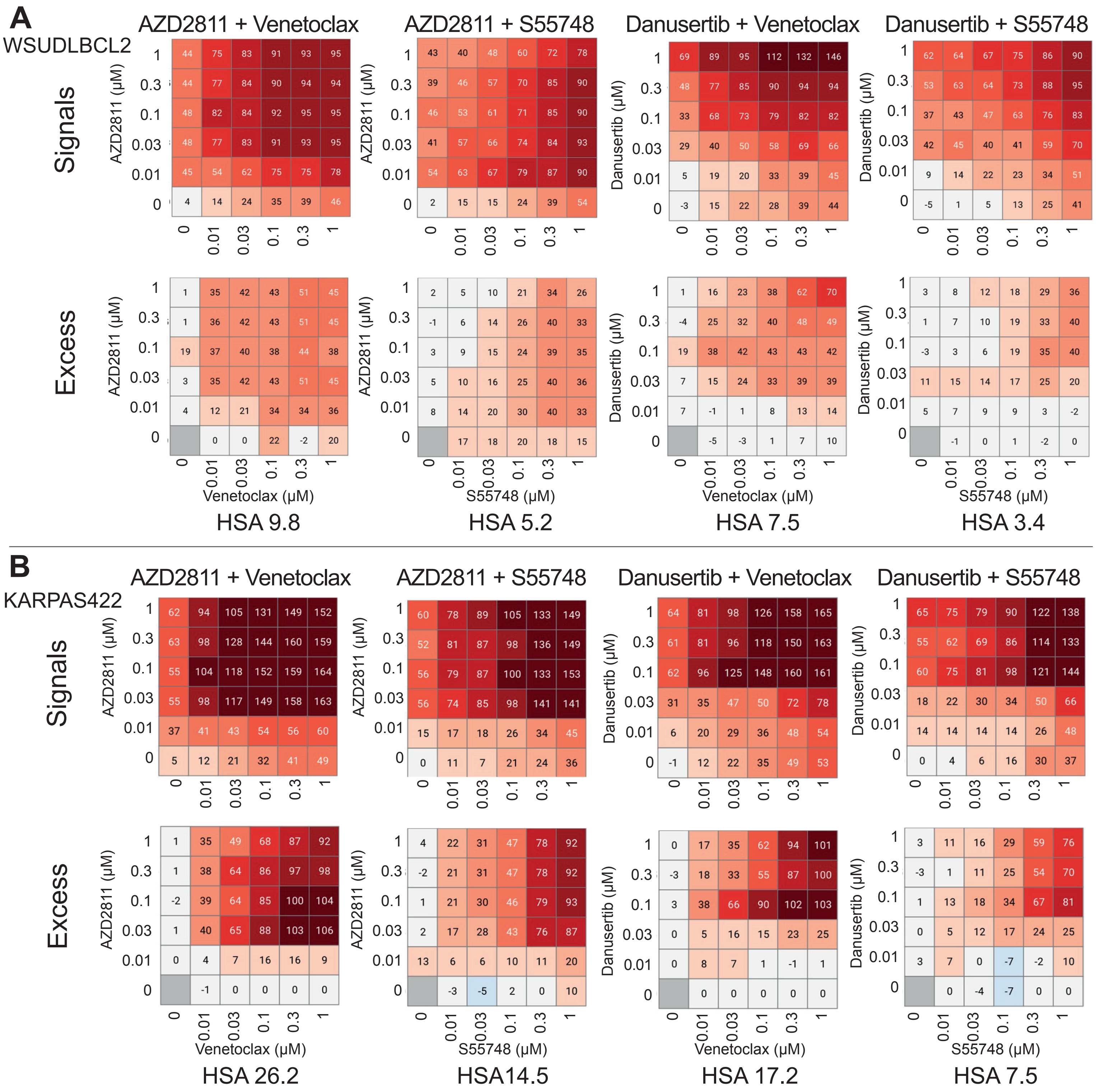
A
WSUDLBCL2
AZD2811 + Venetoclax
AZD2811 + S55748
Danusertib + Venetoclax
Danusertib + S55748
Signals
Excess
AZD2811 (µM)
Danusertib (µM)
Venetoclax (µM)
S55748 (µM)
HSA 9.8
HSA 5.2
HSA 7.5
HSA 3.4
B
KARPAS422
AZD2811 + Venetoclax
AZD2811 + S55748
Danusertib + Venetoclax
Danusertib + S55748
Signals
Excess
AZD2811 (µM)
Danusertib (µM)
Venetoclax (µM)
S55748 (µM)
HSA 26.2
HSA14.5
HSA 17.2
HSA 7.5

Supplementary Figure 11: Effect of Aurora kinase B or pan Aurora kinase plus BCL2 inhibition on cell viability in DLBCL cell lines

Cell viability and HSA excess following treatment for 3 days with a 6 x 6 drug combination matrix in A) WSUDLBCL2 B) KARPAS422 cells. Cells were treated with Aurora kinase B inhibitor AZD2281 and pan-Aurora kinase inhibitor danusertib in combination with BCL2 inhibitors venetoclax and S55748. In the top panels, zero represents DMSO control, values 1 - 100 represent % growth inhibition and values > 100 represent cell death. HSA excess heatmaps indicate dose range where combination benefit was achieved.

A

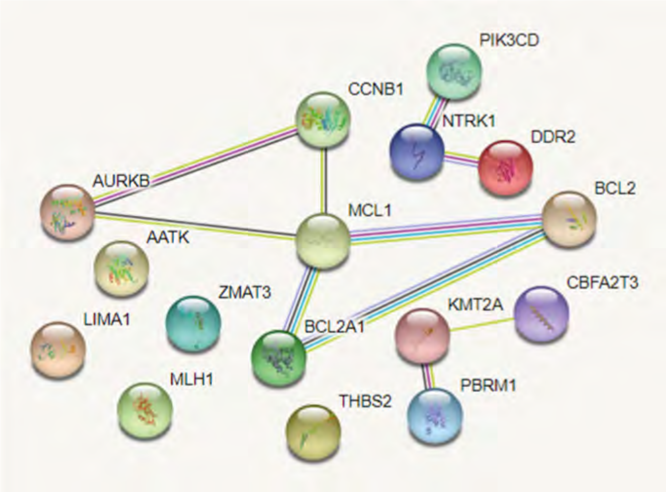

B

AZD5991 (MCL1); AZD5363 (AKT1, AKT2, AKT3) (GDSC_008)

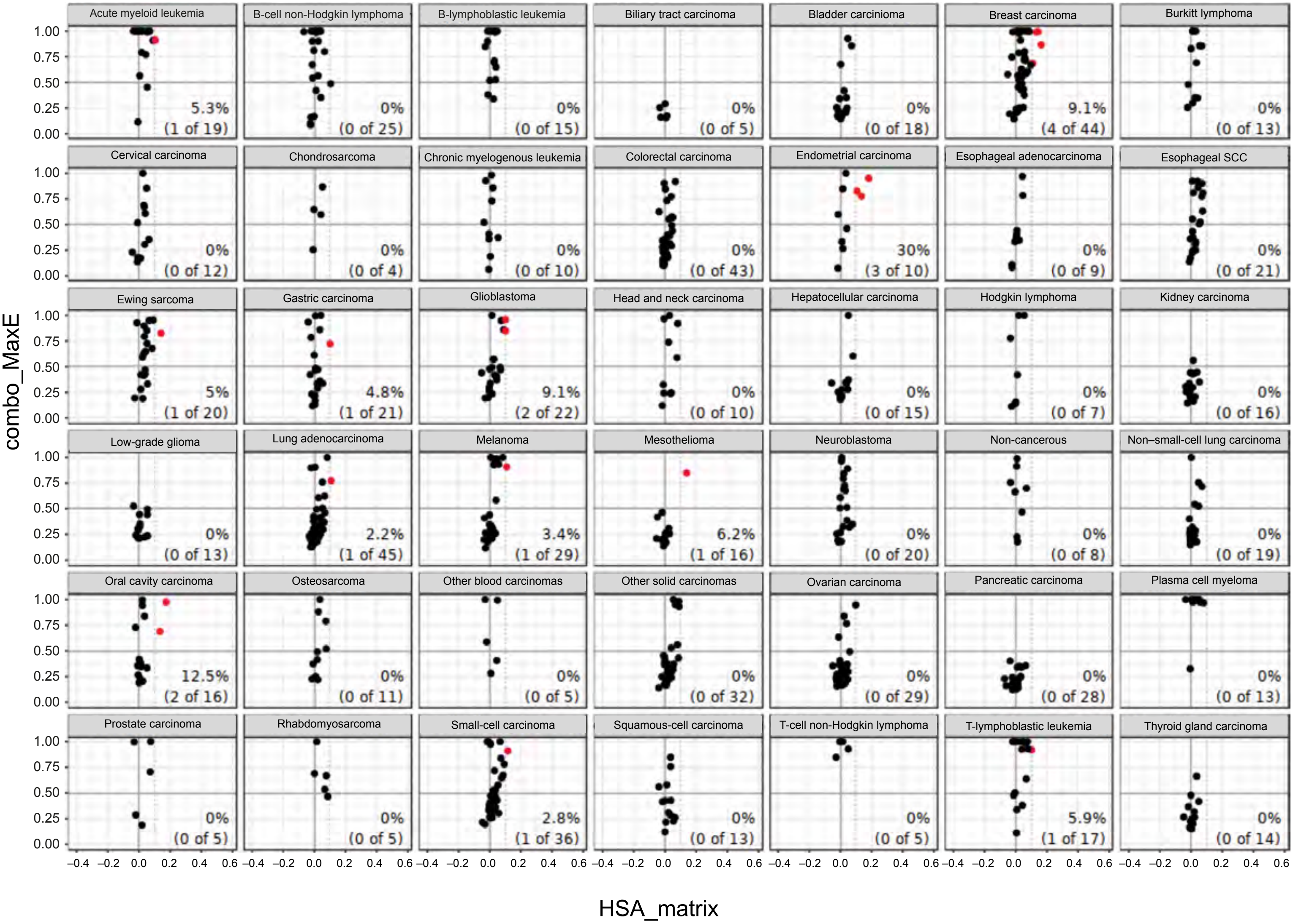

Supplementary Figure 12: network analysis of AZD2811 plus venetoclax biomarkers and targets, and cancer type selectivity of AZD5991 + AZD5363.
A: AZD2811 plus venetoclax STRING analysis of large (effect size >1) and significant ($p<0.05$, FDR <10) combination biomarkers in all or genetic baskets.
B: Capivaerstib plus AZD5991 combination activity (combination Emax versus HSA) in 41 cancer types included in this study. Each dot represents a cell line and cell lines showing high combination activity (combination Emax > 0.5 and HSA > 0.1) are in red.

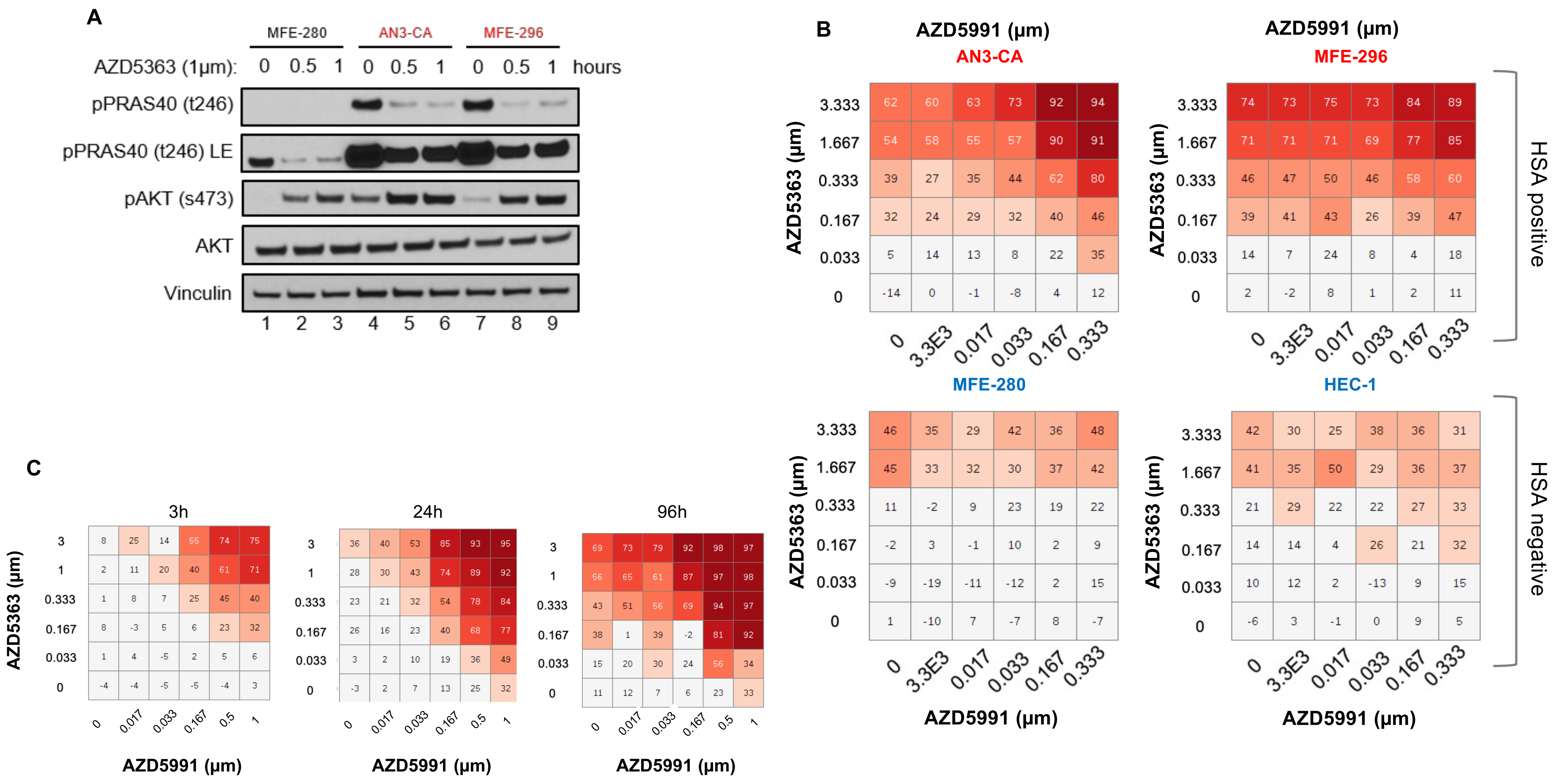
A
MFE-280
AN3-CA
MFE-296
AZD5363 (1μm):
0 0.5 1 0 0.5 1 0 0.5 1 hours
pPRAS40 (t246)
pPRAS40 (t246) LE
pAKT (s473)
AKT
Vinculin
1 2 3 4 5 6 7 8 9
B
AZD5991 (μm)
AN3-CA
MFE-296
MFE-280
HEC-1
AZD5363 (μm)
HSA positive
HSA negative
C
3h
24h
96h
AZD5363 (μm)
AZD5991 (μm)

Supplementary Figure 13: Further analysis of capivasertib (AZD5363) plus AZD5991 in endometrial cell lines

A: Western blot showing elevated phosphorylation of AKT and PRAS40 in AN3-CA, MFE-296 PTEN altered cell lines (in red) compared to PTEN wild-type MFE-280 cells.

B: AZD5991 plus AZD5363 combination activity in HSA positive (AN3-CA, MFE-296) and HSA negative cell lines (MFE-280 and HEC-1).

C: Viability effect after 3, 24 or 96 hour treatment with AZD5363 + AZD5991.

For all experiments, except where indicated otherwise, cell viability was measured after 72 hours using CellTiter-Glo.

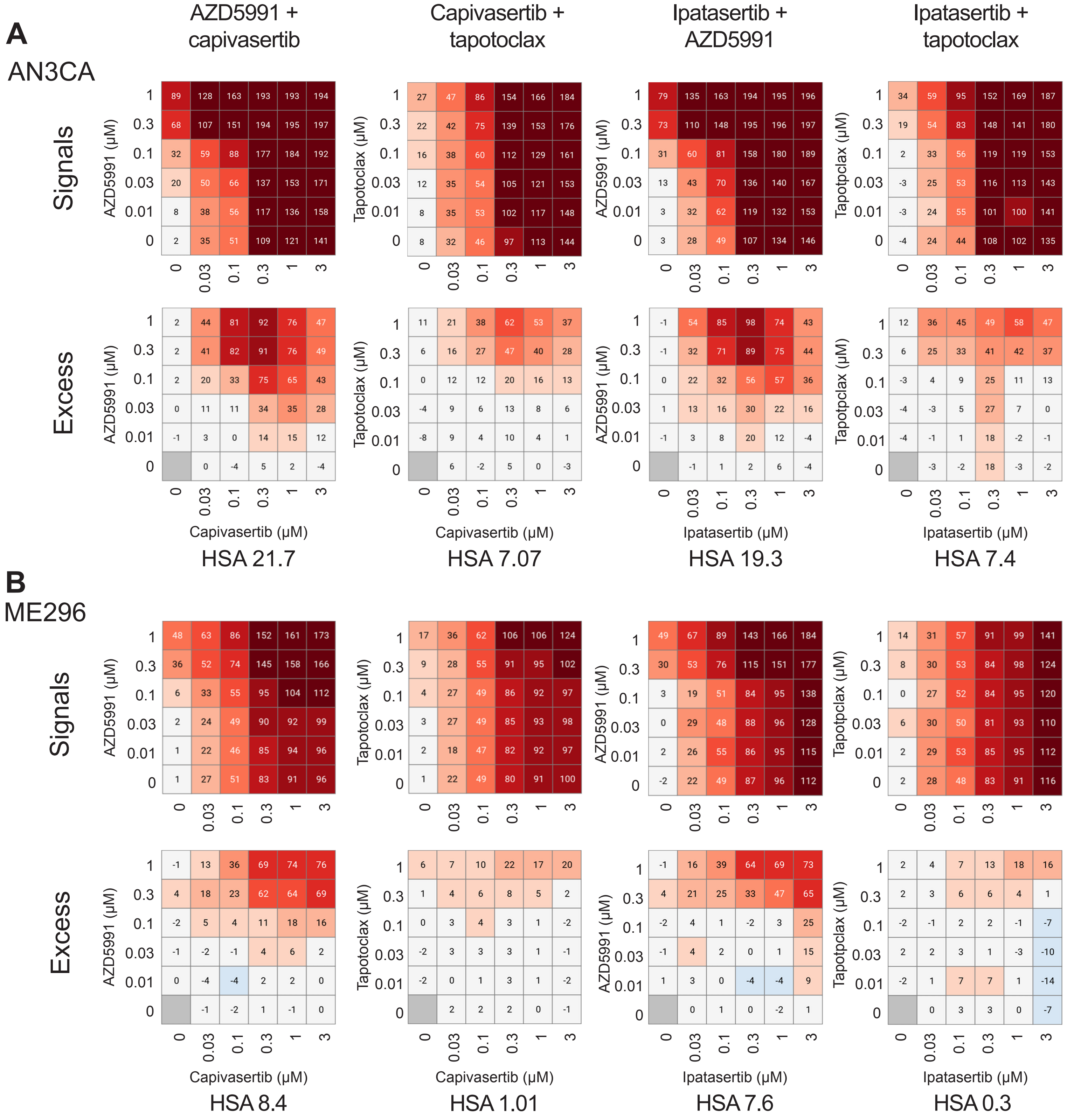
A
AN3CA
AZD5991 + capivasertib
Capivasertib + tapotoclax
Ipatasertib + AZD5991
Ipatasertib + tapotoclax
Signals
Excess
AZD5991 (μM)
Tapotoclax (μM)
Tapotpclax (μM)
Capivasertib (μM)
Ipatasertib (μM)
HSA 21.7
HSA 7.07
HSA 19.3
HSA 7.4
B
ME296
HSA 8.4
HSA 1.01
HSA 7.6
HSA 0.3

Supplementary Figure 14: Effect of pan AKT inhibition plus MCL1 inhibition on cell viability in endometrial cell lines

Cell viability and HSA excess following treatment for 3 days with a 6 x 6 drug combination matrix in (A) AN3CA (B) MFE296 cells. Cell lines were treated with AKT inhibitors capivasertib and ipatasertib in combination with MCL1 inhibitors AZD5991 and tapotoclax. In the top panels, zero represents DMSO control, values 1 - 100 represent % growth inhibition and values > 100 represent cell death. HSA excess heatmaps indicate dose range where combination benefit was achieved.

AKT

MK2206 (μm) \ AZD5991 (μm)	0	0.017	0.033	0.167	0.5	1
3.333	24	32	54	82	84	82
1.667	18	33	40	77	84	82
0.333	25	26	29	49	64	64
0.167	8	7	10	32	32	45
0.033	16	18	16	37	28	30
0	-3	18	8	15	15	21

PI3Kβ/δ

AZD8186 (μm) \ AZD5991 (μm)	0	0.017	0.033	0.167	0.5	1
3.333	28	20	42	73	85	84
1.667	12	12	41	69	68	68
0.333	9	7	23	19	51	44
0.167	22	5	4	14	20	8
0.033	12	19	17	6	8	3
0	1	7	17	9	21	22

PI3Kα

BYL719 (μm) \ AZD5991 (μm)	0	0.017	0.033	0.167	0.5	1
3.333	14	19	32	52	57	59
1.667	4	9	28	33	46	56
0.333	4	1	7	11	19	38
0.167	2	-3	1	7	10	9
0.033	-2	-1	-1	-2	6	-2
0	-2	-2	-5	0	5	3

mTOR1

Rapamycin (μm) \ AZD5991 (μm)	0	0.017	0.033	0.167	0.5	1
3.333	-5	0	16	1	4	17
1.667	-3	-1	20	1	17	15
0.333	-8	-2	-6	-2	16	16
0.167	-5	-1	8	2	10	7
0.033	-12	-5	-4	-3	15	3
0	-1	-4	2	3	2	8

Supplementary Figure 15: Further analysis of capivasertib (AZD5363) plus AZD5991 and other inhibitors in endometrial cell lines

A: Cell viability after exposure to AZD5991 combined with AZD5363 (AKT inhibitor), AZD8186 (PI3Kβ/δ inhibitor), BYL791 (PI3Kα inhibitor) and Rapamycin (mTOR inhibitor). For all experiments, except where indicated otherwise, cell viability was measured after 72 hours using CellTiter-Glo.

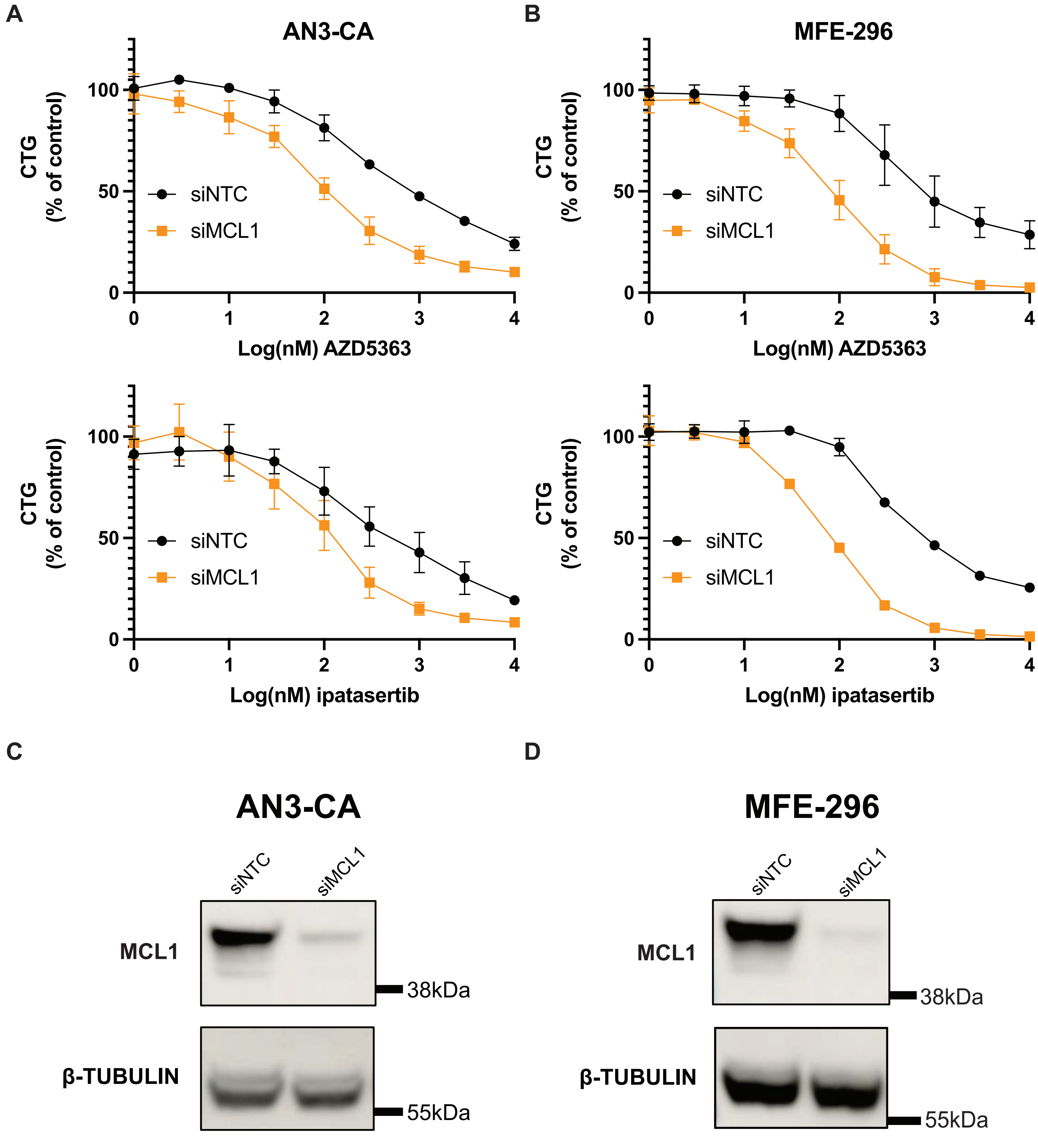
A
AN3-CA
CTG (% of control)
siNTC
siMCL1
Log(nM) AZD5363
B
MFE-296
CTG (% of control)
siNTC
siMCL1
Log(nM) AZD5363
CTG (% of control)
siNTC
siMCL1
Log(nM) ipatasertib
CTG (% of control)
siNTC
siMCL1
Log(nM) ipatasertib
C
AN3-CA
siNTC
siMCL1
MCL1
38kDa
β-TUBULIN
55kDa
D
MFE-296
siNTC
siMCL1
MCL1
38kDa
β-TUBULIN
55kDa

Supplementary Figure 16: Effect of MCL1 genetic knockdown plus pan AKT inhibition on cell viability in endometrial cancer cell lines.

A and B: siRNA knockdown of MCL1 increases sensitivity to AKT inhibitors AZD5363 and ipatasertib in (A) AN3-CA and (B) MFE-296 cells Responses are from the mean of three biological replicas. NTC = non-targeting control siRNA.

C: AN3-CA and (D) MFE-296 cells were reverse transfected with MCL1 or control siRNAs and after 72 hrs knockdown was confirmed by Western blot. Data are representative of two independent experiments.
