# Supplementary material for: Large-scale pan-cancer cell line screening identifies actionable and effective drug combinations
Source: Cancer Discov. Author manuscript; Available in PMC 2024 May 3. (PMC11061612; doi:10.1158/2159-8290.CD-23-0388)
Supplement: Supplementary table legend [file EMS194010-supplement-Supplementary_table_legend.docx]

### Supplementary Tables

1. Supplementary Table 1 lists cell lines used in this study.
2. Supplementary Table 2 lists drugs and drug combinations used.
3. Supplementary Table 3 shows combination-cancer type pairs with 10 percent or more responder cell lines.
4. Supplementary Table 4 shows combination-cancer type pairs with less than 10 percent responder cell lines.
5. Supplementary Table 5 shows combinations with broad activity across tumor types.
6. Supplementary Table 6 shows combination-cancer type pairs with selective activity.
7. Supplementary Table 7 shows the top combination-cancer type pairs in hematological cancers showing activity in at least 10% of tested cell lines in that specific cancer type ranked based on their activity (% responders) and disease selectivity.
8. Supplementary Table 8 shows the top 100 combination-cancer type pairs in solid tumors showing activity in at least 10% of tested cell lines in that specific cancer type ranked based on their activity (% responders) and disease selectivity.
9. Supplementary Table 9 shows the top 50 combinations (all cancer types) using different % response scoring thresholds
10. Supplementary Table 10 shows the number of hits using different % response scoring thresholds
11. Supplementary Table 11 lists combination drug categories.
12. Supplementary Table 12 shows the number of top combinations:cancer type pairs hits in each combination drug category.
13. Supplementary Table 13 shows significant biomarkers and significant emergent biomarkers.
14. Supplementary Table 14 shows the top 100 significant emergent biomarkers.
15. Supplementary Table 15 shows biomarkers associated with top 5 enriched pathways for each drug combination category used in this study (CD = cell death, CS = cell signaling).
16. Supplementary Table 16 shows emergent biomarkers associated with top 5 enriched pathways for each drug combination category used in this study (CD = cell death, CS = cell signaling).
17. Supplementary Table 17 shows large effect size and significant Bliss and combo Emax biomarkers for selected top hits.
18. Supplementary Table 18 shows drug response data for single agents and combination treatments.
